# Proto-oncogenes in a eukaryotic unicellular organism play essential roles in plasmodial growth in host cells

**DOI:** 10.1186/s12864-018-5307-4

**Published:** 2018-12-06

**Authors:** Kai Bi, Tao Chen, Zhangchao He, Zhixiao Gao, Ying Zhao, Yanping Fu, Jiasen Cheng, Jiatao Xie, Daohong Jiang

**Affiliations:** 1 State Key Laboratory of Agriculture Microbiology, Huazhong Agricultural University, Wuhan, 430070 Hubei Province People’s Republic of China; 2 Provincial Key Laboratory of Plant Pathology of Hubei Province, College of Plant Science and Technology, Huazhong Agricultural University, Wuhan, 430070 Hubei Province People’s Republic of China

**Keywords:** *Plasmodiophora brassicae*, Proto-oncogenes, Clubroot, Cancer, Tumor, *Brassica napus*

## Abstract

**Background:**

The eukaryotic unicellular protist *Plasmodiophora brassicae* is an endocellular parasite of cruciferous plants. In host cortical cells, this protist develops a unicellular structure that is termed the plasmodium. The plasmodium is actually a multinucleated cell, which subsequently splits and forms resting spores. The mechanism for the growth of this endocellular parasite in host cell is unclear.

**Results:**

Here, combining de novo genome sequence and transcriptome analysis of strain ZJ-1, we identified top five significant enriched KEGG pathways of differentially expressed genes (DEGs), namely translation, cell growth and death, cell communication, cell motility and cancers. We detected 171 proto-oncogenes from the genome of *P. brassicae* that were implicated in cancer-related pathways, of which 46 were differential expression genes. Three predicted proto-oncogenes (*Pb-Raf1*, *Pb-Raf2*, and *Pb-MYB*), which showed homology to the human proto-oncogenes *Raf* and *MYB*, were specifically activated during the plasmodial growth in host cortical cells, demonstrating their involvement in the multinucleate development stage of the unicellular protist organism. Gene networks involved in the tumorigenic-related signaling transduction pathways and the activation of 12 core genes were identified. Inhibition of phosphoinositol-3-kinase relieved the clubroot symptom and significantly suppressed the development process of plasmodia.

**Conclusions:**

Proto-oncogene-related regulatory mechanisms play an important role in the plasmodial growth of *P. brassicae*.

**Electronic supplementary material:**

The online version of this article (10.1186/s12864-018-5307-4) contains supplementary material, which is available to authorized users.

## Background

*Plasmodiophora brassicae* Woron. is an obligate intracellular plant parasite in the protist subgroup Rhizaria [1]. It is one of the most economically important pathogens of cruciferous plants [2]. *P. brassicae* induces galls on the infected roots of cruciferous plants, such as oilseed rape and cabbage [3]. It severely disrupts the host root functions by inducing the formation of deformed galls, which reduce the uptake of water and nutrients from the soil and the growth of the roots [4]. Clubroot causes huge economic losses to oilseed rape and cruciferous vegetable crops, accounting for up to 10–15% loss of cruciferous crops production globally [5, 6].

When host signals (root exudates) are sensed, the resting spores of *P. brassicae* germinate and release primary zoospores. The zoospores attach to and invade the root hairs. *P. brassicae* forms primary plasmodia and primary zoosporangia in root hairs. This process is termed the asymptomatic root hair infection stage [2, 7]. The secondary zoospores are released from the broken root hairs and directly invade host cortical cells. *P. brassicae* forms galls on host roots or rootlets by the modification of hormone levels [8–11]. In the cortical cells, *P. brassicae* form the secondary plasmodia [12]. In each plasmodium, the nuclei continuously divide, but the plasmodium does not split. Thus, a plasmodium is a single cell with multiple nuclei. After meiotic cleavage, the multinuclear plasmodium returns to the haploid state [13]. Finally, the internal space of plant cells become filled with the mature resting spores [12].

The genome sequences of the single spore isolate Pbe3, Pb3, and Pb6 of *P. brassicae* have been determined. In addition, studies have shown that the significant reduction in intergenic space and low repeat content contribute to the compact genome of *P. brassicae*, and some genes involved in the regulation of the plant growth hormones (cytokinin and auxin) and ancestry of chitin synthases have been identified [3, 14]. These genome data have helped clarify the biological properties of *P. brassicae*. However, the mechanisms involved in special developmental stages of *P. brassicae*, especially the cell division events of multinucleate secondary plasmodia during cortical infection, remain unclear. This prompted us to investigate the molecular regulation mechanisms of the pathogen in response to growth and development characteristics by the combined de novo genome sequencing and transcriptome analysis of cell type-specific stages.

Multicellular organisms have evolved more sophisticated, higher-level functional capability by a division of labor among component cells with complementary behaviors [15]. However, dissolution and death of multicellular individuals due to conditions like cancer occur when the cooperation of component cells in multicellular species breaks down [16, 17]. When cells of multicellular organism fail to regulate their growth within the normal program of development, they face the challenge of cancer, which has often been described as a loss of multicellularity.

Unlike the closely regulated and controlled growth of normal cells, most malignant cells have some common features, including a strong proliferative activity, self-sufficiency in growth signals, limitless regulation of replicative potential (in which antigrowth signals are ignored and replication continues in the presence of a growth signal), and evasion of cell death by a variety of pathways [18]. Cancer occurs in almost all metazoans in which adult cells proliferate, which suggests that the regulatory mechanism of tumorigenic cells is deep-rooted in the evolutionary history of metazoans [19]. The *c-myc* proto-oncogene encodes a transcription factor (Myc) with oncogenic potential. Genetic studies of an ancestral *myc* proto-oncogene from Hydra have dated the human oncogene *myc* back at least 600 million years [20, 21]. It has been argued that oncogenes are ancient and highly conserved, and that cancer cells are not newly evolved types of cell, but rather are heirs to a basic mode of survival that is deeply embedded in multicellular life. In this scenario, cancer is an atavistic state of multicellular life [19].

The possibility of the existence of cancer in other multicellular organisms or even in unicellular protozoa is contentious [22–24]. Despite the demonstration that differentiated plant cells have the unique potential of reverting to a pluripotent state, proliferating, and transdifferentiating, the current knowledge suggests that plant cells are highly resistant to oncogenic transformation and strikingly tolerant to altered levels of cell-cycle regulators and to hyperplasia [25]. So far, it is still unclear whether the regulatory mechanism of tumorigenic cells is involved in the growth and development of unicellular protozoa.

The current study is an initial multi-omics analysis aimed at providing novel insights into the regulatory pattern of the critical multinucleate cell division lifestyle of *P. brassicae*. The data suggest that mechanisms of the proto-oncogenes are highly conserved and deep-rooted in evolutionary history, and play important roles in the specific developmental stage of the examined eukaryotic unicellular organism.

## Results

### Cell type-specific transcriptome analysis of *P. brassicae*

Adopting a whole-genome shotgun sequencing strategy, a combined de novo genome sequence data and cell type-specific transcriptome analysis of *P. brassicae*, including multinucleate secondary plasmodia stage, we constructed the final 24.1 Mb genome assembly with high-quality clean reads.

**Fig. 1:**
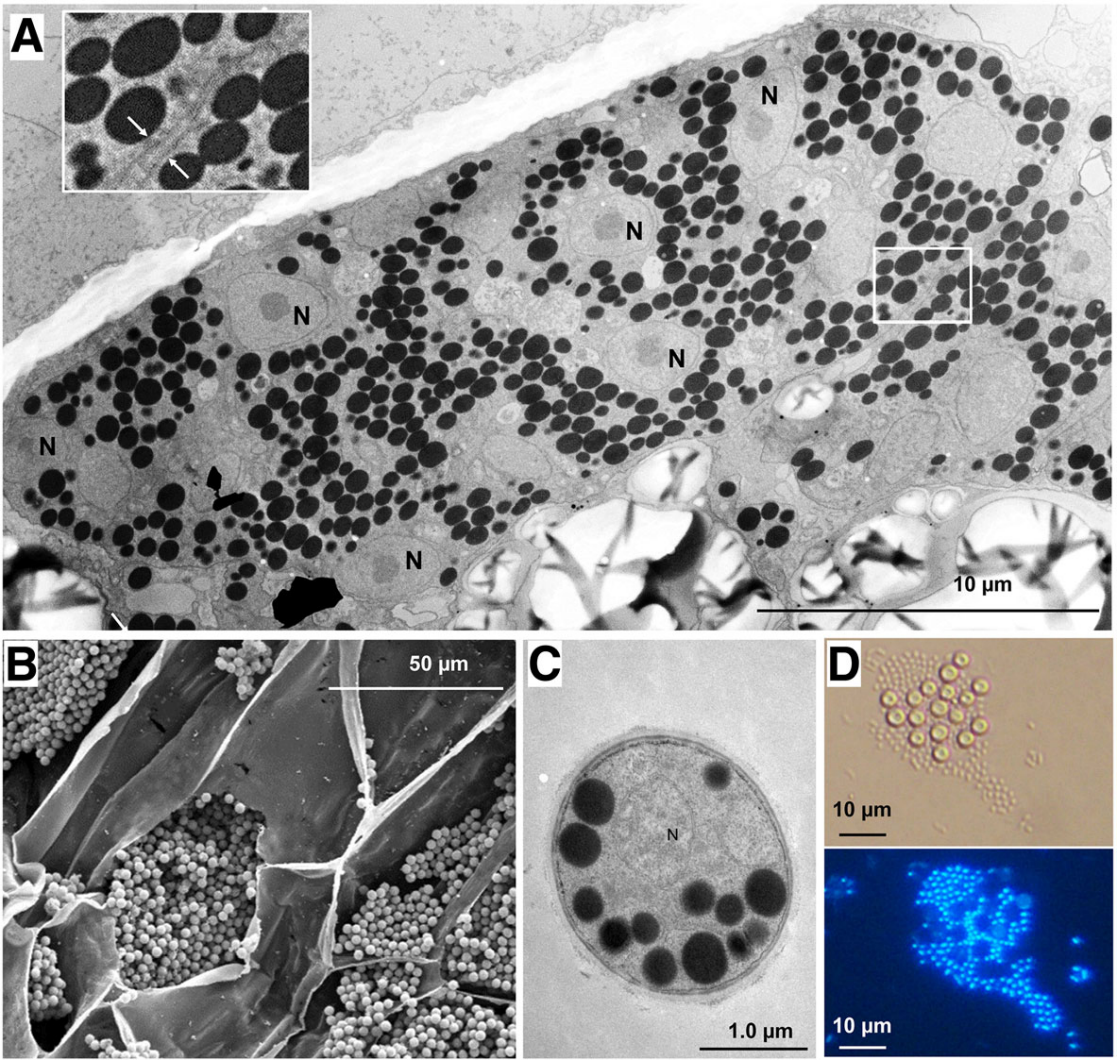
Plasmodia, resting spores, and germination of resting spores (zoospores) of *P. brassicae.* **a** Multinucleate plasmodia in a root cortical cell of *B. napus*; the left upper corner shows an enlarged portion from the white square frame showed membranes of two plasmodia (white arrows); N, nucleus. **b** Mature resting spores in cortical cells of roots of *B. napus*. **c** Resting spores observed by transmission electron microscopy. **d** Germinated resting spores (large) and zoospores (small) stained with 4′,6-diamidino-2-phenylindole. The resting spores are empty, with no nuclei present

During the cortical stage of its life cycle, *P. brassicae* can be multinucleate in a cell termed the plasmodium (Fig. 1a). The transition from the unicellular state to the multinucleate secondary plasmodium with a rapidly proliferating cell type is an important course of cell developmental. It leads to the formation of galls containing mononucleate resting spores that occupy most of the cell [12, 26] (Fig. 1b, c). To investigate the transition from unicellular cells to multinucleate secondary plasmodia, we conducted transcriptome analysis of *P. brassicae* at three time points-the unicellular resting spore (RS) stage, unicellular germinating resting spore (GS) stage (Fig. 1d), and multinucleate early cortical infection (IN) stage. Compared to the RS stage, in the IN stage 508 genes were up-regulated and 89 genes were down-regulated.

**Fig. 2:**
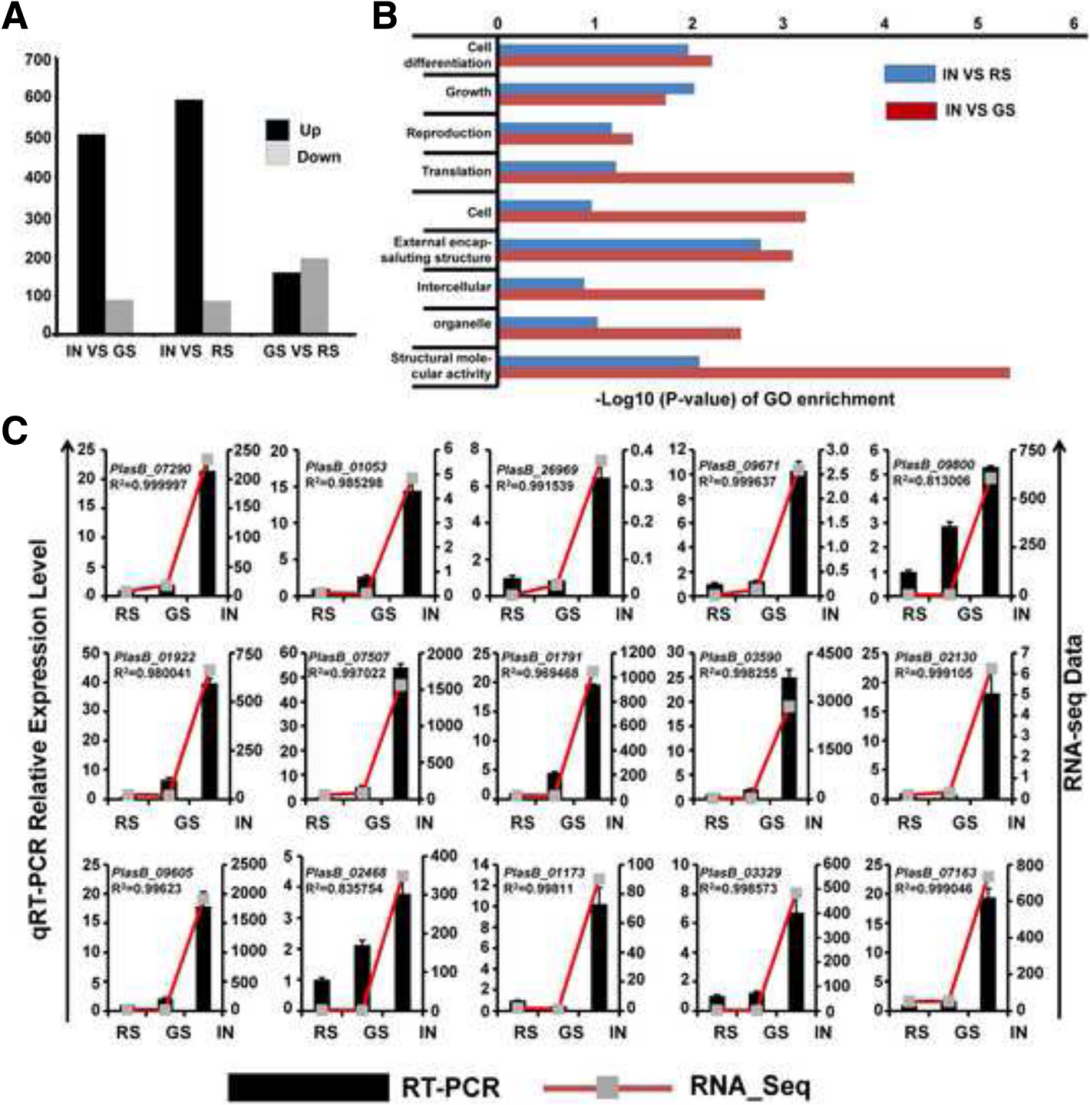
Transcriptome analysis of the entire infection process of *P. brassicae.* **a** DEGs during the infection stages. The early cortical infection stage (IN) was compared with the RS (resting spores) stage and GS (germinating resting spores) stage. **b** Significantly enriched Gene Ontology (GO) terms (*P* < 0.05) of DEGs from the comparison of IN-VS-RS and IN-VS-GS. GO terms belong to biological processes (GOBP), cellular components (GOCC), and molecular functions (GOMF) were shown, respectively. GO terms were sorted based on *P*-values. **c** Validation of GO enrichment of DEGs by qRT-PCR. Fifteen DEGs from significant GO Classification Enrichment were chosen randomly for qRT-PCR validation. The relative expression level of each gene was expressed as the fold-change between three different samples (RS, GS, IN) in the RNA-Seq data (red line chart) and qRT-PCR data (black histogram). Data from qRT-PCR represent the means and standard deviations (three replications). The actin gene was used as an internal control to normalize the expression data. Pearson’s correlation coefficient (*R*-value) was used to measure the consistency of the RNA-seq data and qRT-PCR. DEGs from Biological Process: PlasB_07290, PlasB_01053, PlasB_26969, PlasB_09671; DEGs from Cellular Component: PlasB_02130, PlasB_09605, PlasB_02486, PlasB_01173, PlasB_03329, PlasB_07163; DEGs from Molecular Function: PlasB_09800, PlasB_01922, PlasB_07507, PlasB_01791, PlasB_03590. See Additional file 4: Table S2 for putative functions of these genes. See Additional file 5: Table S3 for genes information

Furthermore, compared with the GS stage, the germination of the resting spores and release of primary zoospores in the IN stage were associated with the up-regulation of 594 genes and the down-regulation of 86 genes (Fig. 2a). The majority of the differentially expressed genes (DEGs) were up-regulated during the IN stage with multinucleate secondary plasmodia (Fig. 2a and Additional file 1: Figure S1), suggesting that the transition from unicellular to the multinucleate state is critical for the whole cell developmental biology of *P. brassicae*.

To confirm the RNA-Seq profiles, qRT-PCR was conducted on 12 randomly selected DEGs. The data between RNA-Seq and qRT-PCR of the genes displayed a high correlation, indicating the basic consistency between the two approaches (Additional file 2: Figure S2 and and Additional file 3: Table S1). To further understand the function of these DEGs, gene ontology (GO) term enrichment analysis was performed. The GO categories were ranked based on *P*-values. The significantly enriched classes (*P* ≤ 0.05) are presented. For these DEGs during multinucleate secondary plasmodia stage, the most significantly enriched GO terms were GO: 0030154 cell differentiation, GO: 0040007 growth, GO: 0000003 reproduction, GO: 0006412 translation in biological processes level, GO: 0005623 cell, GO: 0030312 external encapsulating structure, GO: 0005622 intracellular, GO: 0043226 organelle, and GO: 0005198 structural molecule activity in molecular functions level (Fig. 2B). Furthermore, qRT-PCR verified that 15 randomly selected genes from the significant GO Classification Enrichment were dramatically activated during the multinucleate secondary plasmodia stage (Fig. 2c and Additional file 3: Table S1). Cell type-specific transcriptome analysis revealed the conversion from unicellular to multinucleate status during *P. brassicae* cell developmental.

### Proto-oncogene mechanism involved in the specific developmental stage of multinucleate plasmodium

Infected plant roots transform into galls, with multinucleate secondary plasmodia cell division taking place rapidly. In the process, the secondary zoospores first become myxamoebae and then invade internal root tissues, where they become multinucleate secondary plasmodia [26]. The spherical or subspherical young plasmodia divide into several small plasmodia and the small multinucleate plasmodia form a cluster by repeated cell division. The plasmodia fuse with each other, which is followed by the development of vegetative plasmodia. At the end of cortical infection stage, the mature resting spores form [27] (Fig. 1b).

**Fig. 3:**
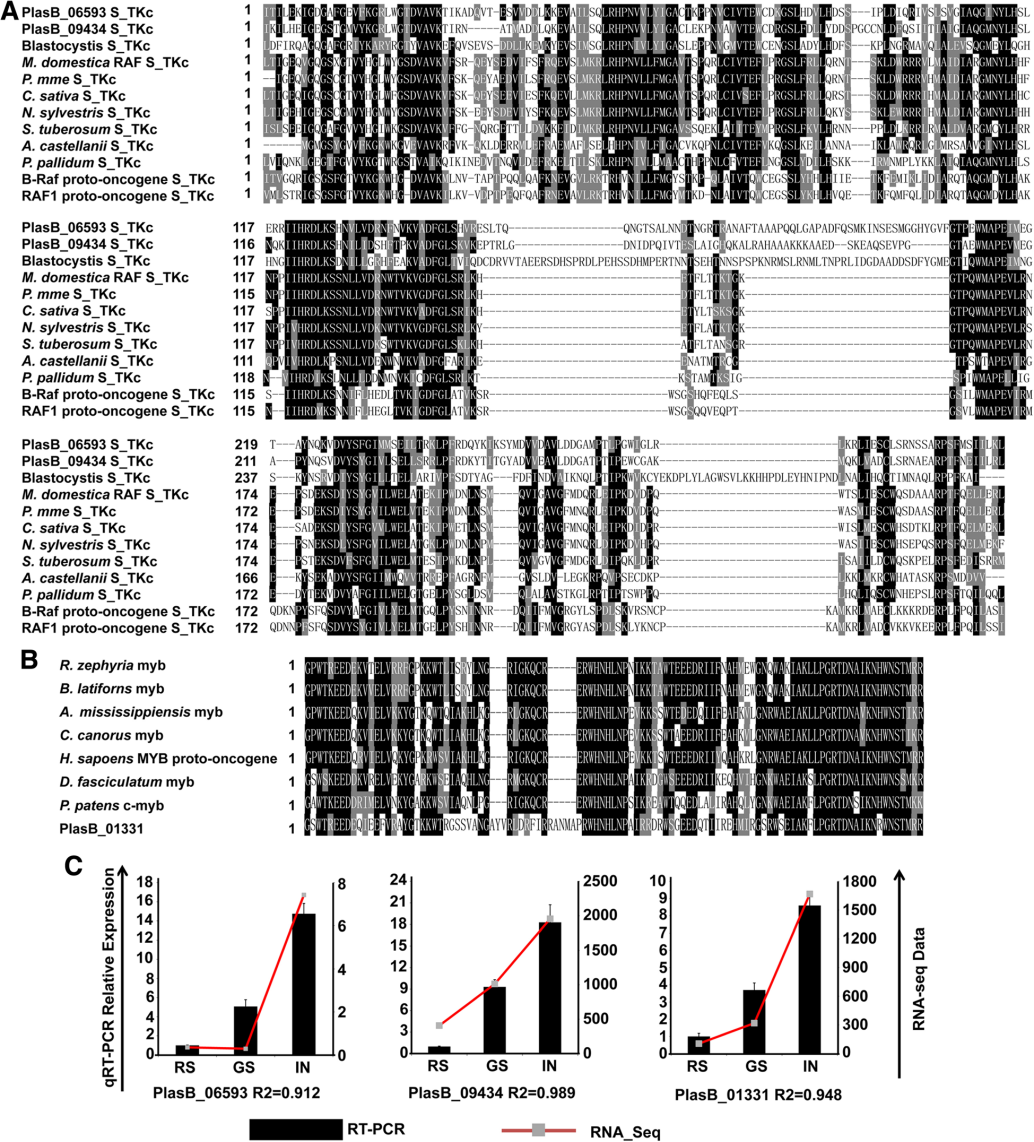
Multiple alignment of conserved domains of proto-oncogenes proteins and cell type-specific expression patterns of these proto-oncogenes. **a** Alignment of S_TKc domain of *P. brassicae* Raf proto-oncogenes with selected homologs. (GenBank accession nos.: *Blastocystis* sp., OAO16337.1; *Malus domestica*, XP_008390530.1; *Prunus mume*, XP_008223365.1; *Camelina sativa*, XP_019092831.1; *Malus domestica*, XP_008390530.1; *Nicotiana sylvestris*, XP_009791599.1; *Solanum tuberosum*, XP_015169630.1; *Acanthamoeba castellanii*, XP_004334709.1; *Polysphondylium pallidum*, EFA76341.1; *Homo sapiens* B-raf proto-oncogene protein, AAA96495.1; *Homo sapiens* RAF1 protein, AAA60247.1). **b** Alignment of myb domain of *P. brassicae* proto-oncogene protein Pbmyb with its homologs. (GenBank accession nos.: *Rhagoletis zephyria*, XP_017478845.1; *Bactrocera latifrons*, XP_018796786.1; *Alligator mississippiensis*, XP_019340180.1; *Camelina sativa*, XP_019092831.1; *Homo sapiens* MYB proto-oncogene protein, AAA52031.1; *Dictyostelium fasciculatum*, XP_004355527.1; *Physcomitrella patens*, AAF78887.1). Identical residues are shaded in black and gray, and gaps are indicated by dashed lines. Alignments were generated by using the ClustalW algorithm with additional manual adjustments. **c** The cell type-specific expression patterns of proto-oncogenes from *P. brassicae* were measured by RNA-seq data (red line chart) and qRT-PCR data (black histogram). The *P. brassicae* actin gene was used as an internal control to normalize the expression data. The histograms and error bars represent means and standard deviations of qRT-PCR, respectively. Pearson’s correlation coefficient (*R*-value) was used to measure the consistency of the RNA-seq data and qRT-PCR. See Additional file 3: Table S1 and Additional file 6: Table S4 for detail of genes

Based on Kyoto Encyclopedia of Genes and Genomes (KEGG) pathway analysis, we identified 171 proto-oncogenes that were implicated in the “Cancers” (Human Diseases) related pathways from the *P. brassicae* ZJ-1 genome, of which 46 genes were DEGs (Additional file 4: Table S2). Of these 171 proto-oncogenes, three predicted proto-oncogene proteins from *P. brassicae*-PbRaf-1 (PlasB_06593), PbRaf-2 (PlasB_09434) and Pbmyb (PlasB_01331)-were found to contain conserved functional domains (S_TKc and myb) homologous with Raf and myb proto-oncogenes proteins from *Homo sapiens*, respectively (Fig. 3a, b). Raf and myb proto-oncogenes proteins regulate fundamental cellular processes such as growth, proliferation, differentiation, metabolism, and apoptosis, and the deregulation of *Raf* and *myb* proto-oncogenes is frequently observed in tumorigenesis [28–30]. Presently, these three predicted proto-oncogenes from *P. brassicae* were specifically activated with the rapidly proliferating multinucleate plasmodium cell type (Fig. 3c). This indicated that the proto-oncogene-related cancer cell development pathway may be highly conserved and deeply embedded in multicellular life and in unicellular protists.

**Fig. 4:**
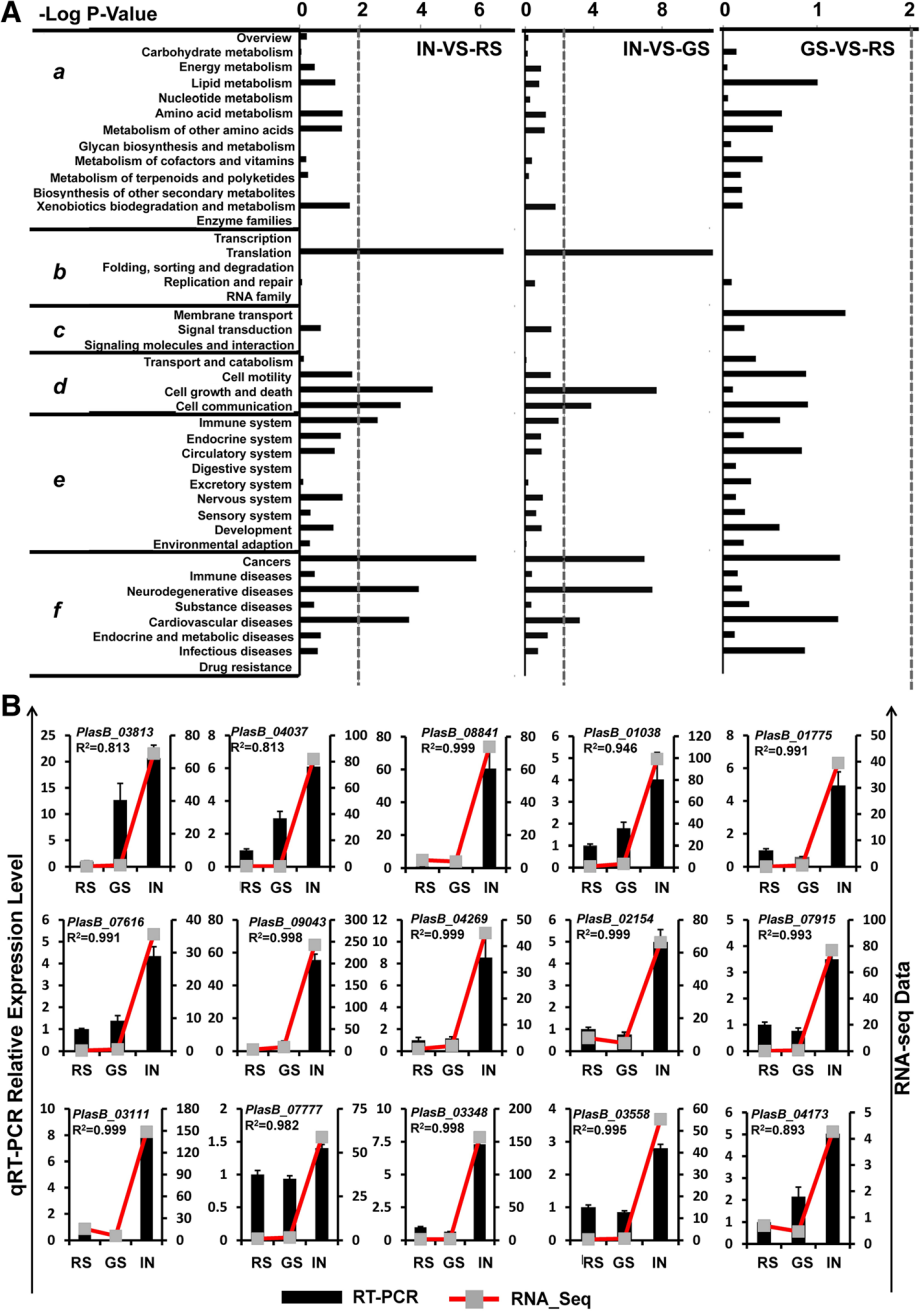
KEGG pathway classification enrichment of DGEs of *P. brassicae* in three developmental stages. **a** Enriched pathways. X-axis, the significance (−Log *P*-value) of KEGG Pathway Enrichment was calculated by the method of hypergeometric distribution; Y-axis, the category of KEGG Pathway (a, Metabolism; b, Genetic Information Processing; c, Environmental Information Processing; d, Cellular Processes; e, Organismal Systems; f, Human Diseases). Vertical dotted lines: *P* = 0.01. **b** Eighteen DEGs from significant KEGG Pathway Classification Enrichment were randomly selected for qRT-PCR validation. The relative expression level of each gene was expressed as the fold change between three different samples (RS, GS, IN) in the RNA-Seq data (red line chart) and qRT-PCR data (black histogram). The *P. brassicae* actin gene was used as an internal control to normalize the expression. Data from qRT-PCR represent the means and standard deviations. Pearson’s correlation coefficient (*R*-value) was used to measure the consistency of the RNA-seq data and qRT-PCR. Up, middle and down panes, DEGs from Translation and DNA replication KEGG Pathway, Cell growth and death KEGG Pathway and Cancers KEGG Pathway, respectively. See Additional file 4: Table S2 for the information of the putative genes

We conducted KEGG pathway classification enrichment analysis on significantly enriched DEGs (*P* ≤ 0.05). During the IN stage, the top five significantly enriched DEGs identified in the KEGG pathway classifications were “Translation” (Genetic information processing), “Cell growth and death”, “Cell communication”, “Cell motility” (Cellular Processes), and “Cancers” (Human Diseases). However, the tendency of this significant enrichment of pathways was no longer evident at the GS stage (Fig. 4a). Furthermore, qRT-PCR analysis verified 18 randomly chosen genes from those significantly enriched in the KEGG pathway. “Translation” and “DNA replication” (Genetic information processing), “Cell growth and death”, “Cell communication”, “Cell motility” (Cellular processes), and “Cancer” (Human diseases) were dramatically activated during the multinucleate plasmodium developmental stage (Fig. 4b and Additional file 3: Table S1). Our results suggest that the “Translation” and “DNA replication”, “Cell growth and death”, “Cell communication”, “Cell motility” and “Cancer” KEGG pathways are distinctively activated and are important in the transition from the unicellular to multinucleate plasmodium states with the rapidly proliferating cell type during cortical infection. The findings reveal the sophisticated mechanism of the proto-oncogene-related pathways by which the pathogen can switch from the normal unicellular state to the continuously dividing tumorigenic state.

### Phosphoinositol-3-kinase (PI3K) inhibitor treatment relieves the severity of clubroot symptom

**Fig. 5:**
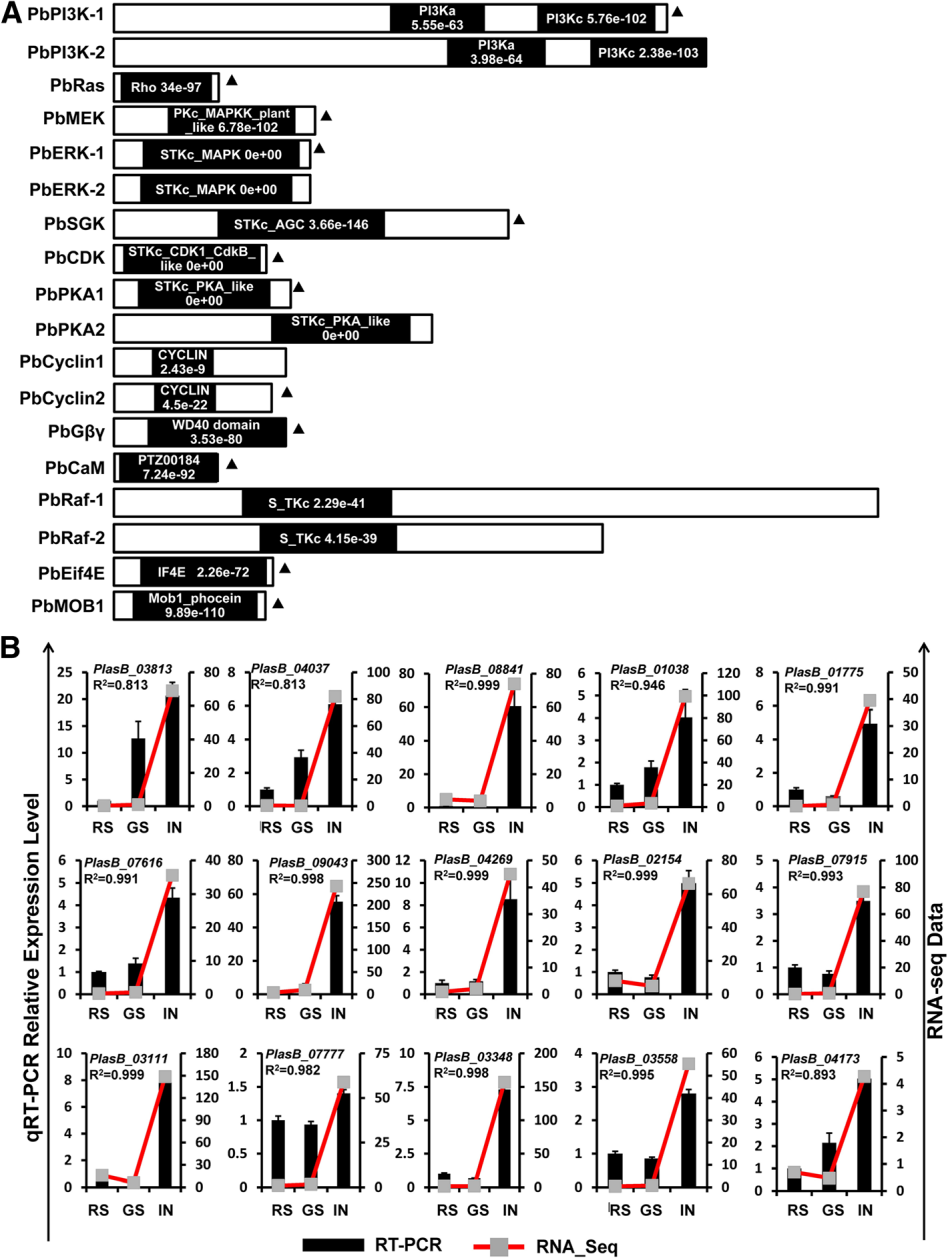
Proteins involved in cancer-related signaling pathways in *P. brassicae* and qRT-PCR validation of the expression pattern. **a** Schematic diagram of proteins encoded by genes of cancer-related signaling pathways in *P. brassicae*. The black frames represented conserved domains in the genes encoded proteins. The information of conserved domain, e-value, and length was obtained from NCBI database. **b** Twelve core genes of cancer-related signaling pathways (marked with black solid triangle in (**a**) were chosen for qRT-PCR validation. Expression levels of these 12 genes from the three different samples (RS, GS and IN) were measured by RNA-seq data (Red line chart) and qRT-PCR data (black histogram). The actin gene of *P. brassicae* was used as an internal control to normalize the expression level. Data from qRT-PCR represent the means and standard deviations (three replications). *R*-value of Pearson’s correlation coefficient was used to measure the consistency of the RNA-seq data and qRT-PCR. See Additional file 3: Table S1 and Additional file 4: Table S2 for genes information

Extensive research over the past decade has revealed the critical role for the signaling transduction pathways in the regulation of the dynamic process of tumorigenesis. The dysregulation of these pathways, for example the Ras/PI3K-Akt and mammalian target of rapamycin (mTOR) signaling pathways, lead to massive overgrowth of tissue [31–33]. The present organization analysis identified 17 gene-encoded proteins containing conserved domains, which were predicted to be involved in cancer-related signaling pathways in the genome of *P. brassicae* (Fig. 5a, Additional file 5: Table S3, and Additional file 6: Table S4). qRT-PCR confirmed the expression pattern of the selected core components of the Ras/PI3K-Akt and mTOR signaling pathways during the developmental stage of the multinucleate plasmodium. The findings indicate the specific activation of the Ras/PI3K-Akt and mTOR signaling pathways during the cortical infection. Compared with other developmental stages, the core genes involved in the Ras/PI3K-Akt signaling pathway (Regulation of actin cytoskeleton), Ras/MAPK signaling pathway (Cell proliferation), PI3K-Akt signaling pathway (Survival signal, Growth and proliferation, Cell cycle progression, Cell survival, Protein synthesis), and mTOR signaling pathway (Translation, Cell growth) were significantly activated in the rapidly proliferating cell stage (Fig. 5b and Additional file 3: Table S1). The transcriptome analysis of *P. brassicae* genes and gene networks involved in tumorigenic-related signaling transduction pathways documented the activation of the core components of the Ras/PI3K-Akt and mTOR signaling pathways, providing insights into the mechanisms by which the parasite can change the normal growth and development into a tumorigenic life-style.

**Fig. 6:**
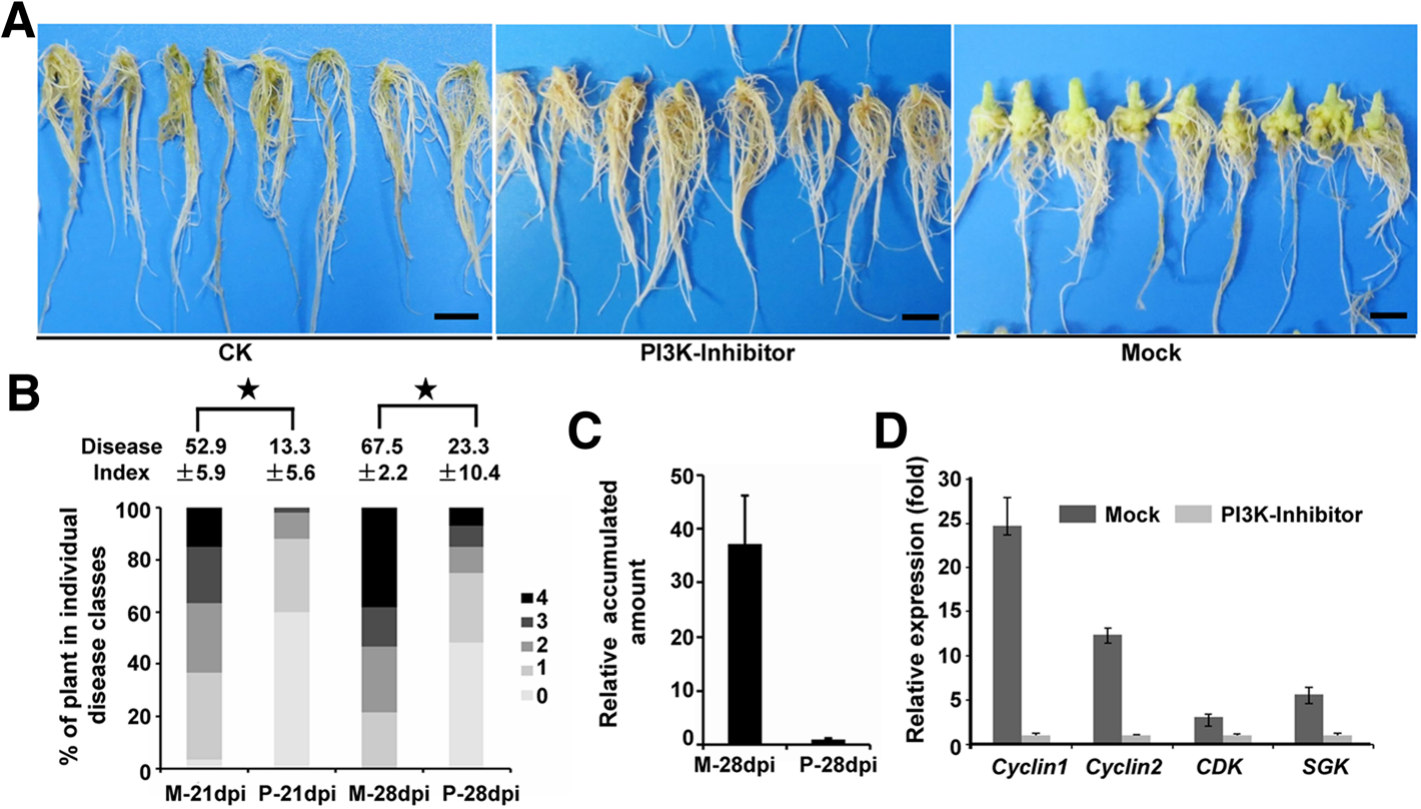
Effect of PI3K inhibitor treatment on the clubroot symptom of *B. napus.* **a** PI3K-treated and *P. brassicae*-inoculated plants showed less symptom and rich rootlets; non-inoculated plants are shown as control (CK), and plants inoculated *P. brassicae* showed heavy symptoms (mock). Photographs were taken at 28 dpi. Bar denotes 1 cm. **b** Clubroot symptoms of infected roots from three biological replicates were evaluated using the percentage of plants in individual disease classes and disease index (DI) at 21 dpi and 28 dpi. Asterisks indicate statistically significant differences at the level of *P* = 0.05. M: Mock-treated P: PI3K inhibitor-treated. **c** At 28 dpi, the infected roots of mock-treated plants and inhibitor-treated plants were harvested. The relative accumulated amount of pathogen DNA was quantified by qPCR. The actin gene of *B. napus* was set as the control to normalize the accumulation amount of the pathogen. M, mock treated; P, PI3K inhibitor-treated. **d** The expression levels of genes from PI3K-related pathways between mock-treated and inhibitor-treated groups. The *P. brassicae* actin gene was used as an internal control to normalize the expression level. The expression level of inhibitor-treated groups was set as 1.0. Data represent the means and standard deviations

Due to the feature of obligate biotrophy, there is still no successful and stable genetic transformation system for *P. brassicae*. Thus, we conducted inhibitor treatment experiments to explore the requirement of tumorigenesis-related signaling transduction pathways during the tumorigenic proliferating process of the multinucleate plasmodium stage. GDC-0032 is a potent, next-generation β isoform-sparing PI3K inhibitor targeting PI3Kα/δ/γ [34, 35]. GDC-0032 treatment affects the normal growth and root formation and plant development of oilseed rape plants (*Brassica napus*). The expression of *Cyclin* (XM_013809141.1) from *B. napus* between the mock-treated and GDC-0032-treated groups did not differ significantly (Additional file 7: Figure S3 a, b). Host plants were inoculated with GDC-0032-pretreated resting spores and then grown in nutrient solution that contained the inhibitor. GDC-0032 had no effect on the germination rate of the resting spores or the root hair infection rate of *P. brassicae* (Additional file 7: Figure S3 c, d). Clubroot symptom was then quantified at 21 and 28 days post-infection (dpi). Clubroot symptom was less frequent in the inhibitor-treated plants with a lower Disease Index (DI) than the mock-treated group (Fig. 6a, b). qPCR was used to evaluate the relative accumulated amount of pathogen in the root tissues of host plants. *P. brassicae* was 37-fold more prevalent in the infected roots of mock-treated group than the inhibitor-treated group (Fig. 6c). Furthermore, the expression levels of the components from PI3K-related signaling transduction pathways were significantly reduced in the inhibitor-treated group (Fig. 6d). These results indicated that GDC-0032 treatment could relieve clubroot symptom of infected roots by inhibiting the core components of PI3K signaling transduction.

## Discussion

### Conversion from unicellular to multinucleate state is the key stage of *P. brassicae* cell development

The conversion from the unicellular state to multinucleate secondary plasmodium is critical in *P. brassicae* cell developmental biology, as the cell division of secondary plasmodia provokes the rapidly cleavage into resting spores, which enable survival during harsh environmental conditions and the initial infection source of the next cycle [12]. The rapidly proliferating cell types of multinucleate plasmodia are part of this conversion. The formation of secondary plasmodia is accompanied by hypertrophy and hyperplasia of the infected host cells, leading to the development of galls that obstruct nutrient and water transport, with resting spores released back into the soil when the galls decompose [14]. Although the transition from the unicellular to the multinucleate state has been the focus of *P. brassicae* infection biology research for a long time [12, 26], there is still a poor understanding of this phase change, in particular, the details of the transcriptional regulatory network of multinucleate secondary plasmodium. Understanding the mechanisms involved in cell division of multinucleate secondary plasmodia could help reveal the universal regulation pattern of cell division and development of multinucleate cells in this type of protist.

Presently, GO enrichment analysis of cell type-specific transcriptome suggested that the activation of cell differentiation, growth, reproduction, and translation related biological processes are required in multinucleate cell division. Based on the functional study of the significantly enriched KEGG pathways, we speculate that many pathways, including “Cancer” (Human Diseases) maybe vital for the conversion from a normal unicellular state to the rapidly proliferating multinucleate cell state.

### Proto-oncogenes of *P. brassicae* are specifically activated during the multinucleate cell division stage

The 171 proto-oncogenes implicated in the “Cancer” (Human Disease) pathway were mined from the genome of strain ZJ-1 by in silico analysis. Proto-oncogenes may be ancient and highly conserved [36]. Such conservation indicates that proto-oncogenes are normal cellular genes homologous to oncogenes, but they have served vital and indispensable functions in normal cellular and organismic physiology, and that their role in carcinogenesis represents only an unusual and aberrant diversion from their usual functions [19, 36]. For example, the deregulation of the *c-myc* proto-oncogene leads to tumorigenesis and is a hallmark of approximately 30% of all human cancers [37, 38]. The ancestral forms of *myc* and *max* genes have been identified and extensively characterized in the early diploblastic cnidarians, *Hydra magnipapillata* [20]. The *myc* gene was observed to be specifically activated in all rapidly proliferating cells, such as the interstitial stem cell system and gland cells. However, the expression of *myc* is not detectable in terminally differentiated nerve cells, nematocytes, or epithelial cells. The results reveal that the stem cell-specific activation of the ancestral *myc* protooncogene is indispensable for the regenerative ability of the early metazoan *Hydra*, which confirms that the principal functions of the Myc master regulator arose very early in metazoan evolutionary history [20].

The present findings corroborate the requirement of 171 proto-oncogenes in the multinucleate cell division stage to regulate proliferation and self-renewal and to perturb or inhibit terminal differentiation, as has been proposed in metazoans. For the S_TKc and myb conserved functional domains, three predicted proto-oncogenes proteins from *P. brassicae* displayed homology with Raf and myb from *H. sapiens*, respectively. These proto-oncogenes were specifically activated during the cell type-specific multinucleate cell division course of *P. brassicae*. Considering the indispensable role of proto-oncogenes from the protist subgroup Rhizaria in cell developmental biology, similar to the rapid cell proliferation of multinucleate plasmodia, we suggest that the proto-oncogenes are a group of genes that have been strongly functionally conserved in metazoans and protists.

### PI3K-related signaling transduction pathways have key roles during multinucleate cell division stage

PI3K-Akt signaling, including downstream signaling pathways, such as the mitogen-activated protein kinase kinase (MEK), extracellular signal-regulated kinase (ERK), mitogen-activated protein kinase (MAPK) pathways and the mTOR pathways, are activated by many types of cellular stimuli or toxic insults. This activation regulates the fundamental cellular functions of transcription, translation, proliferation, growth, and survival [32, 39]. The development and progression of cancer are the result of a disturbance in the balance between cell proliferation and apoptosis [40]. PI3K-Akt signaling is associated with cell proliferation and apoptosis. Notably, many studies have demonstrated that the constitutive activation of the PI3K-Akt pathway is frequently associated with human cancers [32, 41, 42]. These findings indicate that the PI3K-Akt pathway plays a pivotal role in tumor progression. Genes associated with tumorigenic-related signaling transduction pathways were identified from *P. brassicae*. Our transcriptome analysis also revealed the significant up-regulation of core compounds from the Ras/PI3K-Akt and mTOR signaling pathways during the cell division course of multinucleate secondary plasmodia.

The recent studies show that the PI3K pathway and the downstream pathways are often deregulated in human cancer cells. As the key component of these signaling cascades, PI3K an important target for therapeutic interventions [32, 41]. To date, several compounds that directly inhibit PI3K-Akt activity have been developed. In preclinical, phase I or II clinical trials, they have shown good anti-tumor efficacy, such as inducing cell cycle arrest or apoptosis in human cancer cells in vitro and in vivo [43–45]. GDC-0032 is a potent and selective inhibitor of Class I PI3Kα, δ, and γ isoforms. Preclinical data show that the combination of GDC-0032 enhances the activity of other inhibitor medication resulting in tumor regression and tumor growth delay [34, 35]. Intriguingly, the present results show that GDC-0032 can block cell proliferation course of multinucleate secondary plasmodia and relieve the clubroot symptom of the host plants. Our findings indicate that the PI3K related signaling transduction pathways were specially required by *P. brassicae* during the multinucleate plasmodium stage of cortical infection, but not in the root hair infection stage. This is consistent with the distinct activation of proto-oncogenes and related pathways during the development of multinucleate plasmodium with a rapidly proliferating cell type.

Our research highlights the highly concordant mechanism of cell development regulated by the PI3K-Akt signaling pathway between protists and metazoans, and provides insight into the convergent evolution of this regulatory mechanism. Thus, PI3K-Akt-mediated regulation of the transition from normal cell development to rapid proliferation (such as the cell division of multinucleate secondary plasmodia of *P. brassicae* and tumorigenesis of mammalian cells) shares a common developmental mechanism. In the future, the plasmodiophorid material of *P. brassicae* could be a potential model system for studies of the PI3K-Akt signaling pathway mechanisms involved in mammalian cancer.

## Conclusions

Using a multi-omics analysis strategy, we provide an important comprehensive insight into the critical multinucleate cell division life-style of the obligate uncultivable protist pathogen, *P. brassicae*. Our finding that the special activation of proto-oncogenes is important in the development of *P. brassicae* will undoubtedly inform future research on novel regulation mechanisms involved in the growth and development of unicellular organisms.

## Methods

### Collection of field populations and isolation of single spores

*P. brassicae* strain ZJ-1 resting spores were maintained at − 20 °C in sterilized double-distilled water containing 50 μg.mL^− 1^ cefotaxime sodium. The details of the strain and the inoculation methods have been previously published [46].

### Developmental stage-specific sample preparation

To prepare resting spore stage samples, the resting spores were extracted from single spore isolated from plant root clubroot as previously described [46]. For the collection of germinating resting spore stage samples, the purified resting spores were thawed at 4 °C for 48 h followed by 24 h at room temperature in a root exudate solution that allow germination in a dark environment. The root tissues of the prepared seeds were immersed in 2.0 mL Eppendorf tubes with the treated germinating spores (10^7^ spores.mL^− 1^). Samples were harvested after 24 h when microscopy examination revealed appreciable aggregation and adsorption in root tissues of the germinating resting spores. To prepare the IN root samples for RNA extraction, seeds of oilseed rape (*Brassica napus*) were surface sterilized in 1% NaClO for 5 min, washed with distilled water, and germinated on moistened filter paper for 6 days in a growth chamber maintained at 22 °C/20 °C (day/night) with a 14-h photoperiod and 80% relative humidity. Some of the seeds were transplanted to autoclaved potting mix in 60 cell (4 cm × 3.5 cm × 6 cm) plastic pot trays in a controlled environment growth chamber (HP300GS-C; Ruihua Instrument and Equipment, Wuhan, China) at a constant 20 °C with a 14-h photoperiod and 80% relative humidity. Samples were watered once daily. Nineteen days after sowing, the plants were inoculated with resting spores derived from single spore ZJ-1 strain (10^7^ spores.mL^− 1^). After inoculation, the plants were grown in the glasshouse conditions described above. Root masses were collected from at least 50 plants at 21 dpi, when signs of secondary infection were visible. Roots were extensively washed in tap water and inspected microscopically to ensure the absence of successful infection of pathogens. Fine root tissues were trimmed away.

### DNA and RNA extraction

DNA was extracted from resting spores of the strain ZJ-1 using a modified CTAB method [47] and stored at − 20 °C. Total RNA was extracted with RNAiso Plus (Takara, Dalian, China) according to the manufacturer’s protocols from the developmental stage-specific samples described above. The pellet was resuspended in 20–30 μL of diethyl pyrocarbonate-treated, RNase-free water and homogenized by pipetting 20–30 times. Each sample was stored at − 80 °C. RNA quantity and quality was assessed using a spectrophotometer (NanoDrop Technologies, Inc. Wilmington, DE USA) and by 1.5% (*w*/*v*) agarose gel electrophoresis.

### Transcriptome expression analysis

We collected samples from three specific infection stages: resting spore stage, primary zoospore stage, and early cortical infection stage. Total RNA samples of three biological replicates for each stage were prepared. According to the manufacturer’s instructions (Illumina, San Diego, CA), an RNA-seq library was prepared, followed by sequencing on an Illumina NextSeq 500 platform for paired-end 2 × 150 bp sequencing, which was performed at Shanghai Personal Biotechnology Co., Ltd. (Shanghai, China). The RNA-Seq raw reads were processed to obtain high quality reads by removing the adapter sequences and low-quality bases at the 3′ end and trimming low-quality bases (Q < 20) from the 3′ to 5′ ends of the remaining reads. Reads containing ‘N’ and greater than 50 bp were filtered out. The resulting reads were considered for analysis. The filtered reads were mapped to the *P. brassicae* genome using Tophat v2.0.9 (http://tophat.cbcb.umd.edu/) [48]. The analysis of transcriptome differential expression was conducted with HTSeq (http://www-huber.embl.de/users/anders/HTSeq) [49] and DESeq (http://www-huber.embl.de/users/anders/DESeq) [50]. DESeq based on the theory of negative binomial distribution was used to identify the DEGs and their corresponding *P*-values [51]. The DEGs were selected with the standard: P-value≤0.05 and the absolute value of log_2_ Fold Change≥1. GO term enrichment analysis first mapped all DEGs to GO terms in the database (http://www.geneontology.org/), calculating gene numbers for every term, then used the hypergeometric test to identify significantly enriched GO terms in DEGs compared to the genome background. The KEGG pathway enrichment analysis identified significantly enriched pathways of DEGs compared with the entire genome background. To analyze gene expression data and genes with similar functions, we exploited hierarchical clustering methods based on transcriptome expression data. To compare the expression pattern of each gene between samples, the abundance of each transcript was normalized by reads per kilobase of transcript per million mapped reads (RPKM) [52]. The heat map of the clustered genes and samples was generated by the MultiExperiment Viewer v4.9 software package [53]. An average-linkage hierarchical clustering method was used, and Pearson’s correlation coefficient (the distance metric is the default) was employed to measure the similarity of the expressed genes.

### Quantitative RT-PCR validation

qRT-PCR primers were designed to generate amplicons to validate the RNA-seq data (Additional file 3: Table S1). For RT-PCR and qRT-PCR, 5 μg of total RNA was reverse transcribed into first-strand cDNA using the oligo (dT) primer and M-MLV Reverse Transcriptase according to the manufacturer’s instructions (TransScript, Beijing, China). All qRT-PCR experiments were run using SYBR Green Real-Time PCR Master Mix (Bio-Rad, Hercules, CA, USA) in 20 μL reactions with the CFX96™ real-time PCR detection system (Bio-Rad). The *P. brassicae* actin gene was used as an internal control to normalize the expression data. Data was acquired and analyzed using the Bio-Rad CFX Manager™ Software (version 2.0). The relative expression level of each target gene was quantified by the comparative CT method (2 ^-△△Ct^) [54]. The relative expression levels of each gene were validated for the RNA-seq data.

### Symptom quantification during treatment with PI3K antagonist

At 28 days after PI3K inhibitor treatment, growth and development status of oilseed rape plants were checked. A group treated with dimethylsulfoxide (DMSO) was the control. The root tissues of treated plants were harvested and the expression level of the PI3K gene (XM_013869861.1) from *B. napus* was quantified by qRT-PCR as described above. The effect of the PI3K inhibitor on resting spore germination rate and root hair infection rate were analyzed as previously described [55, 56]. At 10 days after germination, the prepared plants were inoculated with the pretreatment resting spores diluted to 10^7^ spores.mL^− 1^ with modified 1/2 Hoagland nutrient solution and inhibitor solution (GDC-0032: 100 nM, MedChemExpress) in 10 mL EP tubes. As a parallel control, 1/2 Hoagland nutrient solution and equivalent DMSO treatment was set as the mock-treated group under the same condition. The CK group (non-inoculated) was added to EP tubes contained equivalent volumes of 1/2 Hoagland nutrient solution and inhibitor solution. Solutions in the EP tubes were refreshed every 7 days. The disease index (DI) was calculated by categorizing the individual roots at 21 and 28 dpi into five classes: 0 (no symptoms); 1 (very small clubs, mainly on lateral roots that do not impair the main root); 2 (small clubs covering the main root and few lateral roots); 3 (medium sized to bigger clubs, also including the main root and hypocotyl, fine roots are partly unaffected, plant growth may be impaired); 4 (severe clubs in lateral, main root, hypocotyls or rosette, fine roots completely destroyed, plant growth is affected) [57], using the following formula:

$$ \mathrm{DI}=\left(1{\mathrm{n}}_1+2{\mathrm{n}}_2+3{\mathrm{n}}_3+4{\mathrm{n}}_4\right)100/4{\mathrm{N}}_{\mathrm{t}} $$

where n_1_–n_4_ are the numbers of plant in the indicated classes and N_t_ is the total number of plant tested. At 28 days after infection, all seedlings were harvested and the clubroot symptoms were investigated by phytopathological analysis. The plants were cut at the top of the hypocotyl into shoots and roots. For quantitative estimation, genomic DNA was prepared from the pool of at least 30 root samples. The abundance of the actin gene of *P. brassicae*, which represents the accumulation amount of the pathogen in the root was normalized to the actin gene of *B. napus*. A similar result was derived from three independent biological experiments. For each biological experiment, at least 30 plants were analyzed.

## Additional files

Additional file 1: **Figure S1**. Heatmap of DEGs of *P. brassicae* at three stages. IN, multinucleate secondary plasmodia stage in plant cortical cells; GS, germinating resting spores stage when the resting spores germinating and releasing primary zoospores; and RS, resting spores stage. (TIF 1974 kb)

Additional file 2: **Figure S2**. Validation of RNA-seq results of *P. brassicae* by qRT-PCR. Comparison of expression levels for the randomly selected 12 genes from the three different samples (RS, GS and IN) were measured by RNA-seq data (gray line chart) and qRT-PCR data (black histogram). The *P. brassicae* actin gene was used as an internal control to normalize the expression level. Data from qRT-PCR represent the means and standard deviations. Pearson’s correlation coefficient (*R*-value) was used to measure the consistency of the RNA-seq data and qRT-PCR. See Additional file 3: Table S1 for primer information. (TIF 736 kb)

Additional file 3: **Table S1**. Genes examined and primer pairs used in this study. (DOCX 38 kb)

Additional file 4: **Table S2**. The genes involve in Cancers related pathways on the genome of *P. brassicae* and corresponding expression pattern. (DOCX 32 kb)

Additional file 5: **Table S3**. The amino acid sequences encoded by genes involved in cancer-related signaling pathways in *P. brassicae*. (DOCX 26 kb)

Additional file 6: **Table S4**. Alignments of *P. brasicae* proteins associated with cancer-related signaling pathways with their homologs. (DOCX 105 kb)

Additional file 7: **Figure S3**. Effect of PI3K inhibitor treatment on the growth, development of oilseed rape plants, resting spores germination rate and root hair infection rate of *P. brassicae*. **a** Growth and development status of oilseed rape plants treated with PI3K inhibitor (right). MOCK (DMSO) treatment served as control (left). The pictures of plants were taken at 28 day after treatment. Bar = 1.5 cm. **b** At 28 day after treatment, the roots of MOCK treated plants and inhibitor treated plants were harvested. The expression level of Cyclin gene (XM_013809141.1, downstream gene of PI3K signaling pathway) in *B. napus* with MOCK and inhibitor treatment was quantified by qPCR. The actin gene of *B. napus* was used as control to normalize the expression level. Data represent the means and standard deviations. The expression level of MOCK treated group was set as 1.0. Statistically significant difference of data between MOCK and inhibitor treated groups was compared, same letter in the graph indicates no significant differences at the level of *P* = 0.05. **c**-**d** Resting spores germination rate and root hair infection rate of *P. brassicae* were compared between H_2_O, MOCK and PI3K-Inhibitor treated groups. At 6 day, the treated spores were stained with orcein (Sigma-Aldrich Canada). The germination rate of spores was counted under microscope. At 7 dpi, the roots of oilseed rape plants were stained with Trypan Blue, then the root hair infection rate was counted with microscopic examination. The graphic data represent the means and standard deviations from three biological replicates. At the level of *P* = 0.05, statistically significant differences of data between H_2_O, MOCK and inhibitor treated groups were compared, same letters in the graph indicate no significant differences. (TIF 642 kb)
