# Correction to: Proto-oncogenes in a eukaryotic unicellular organism play essential roles in plasmodial growth in host cells

**DOI:** 10.1186/s12864-019-5739-5

**Published:** 2019-05-08

**Authors:** Kai Bi, Tao Chen, Zhangchao He, Zhixiao Gao, Ying Zhao, Yanping Fu, Jiasen Cheng, Jiatao Xie, Daohong Jiang

**Affiliations:** 1 State Key Laboratory of Agriculture Microbiology, Huazhong Agricultural University, Wuhan, 430070 Hubei Province People’s Republic of China; 2 Provincial Key Laboratory of Plant Pathology of Hubei Province, College of Plant Science and Technology, Huazhong Agricultural University, Wuhan, 430070 Hubei Province People’s Republic of China

**Correction to: BMC Genomics**

**https://doi.org/10.1186/s12864-018-5307-4**

Following the publication of this article [1], the authors noted the following errors:

1. In the Results section the sentence “Furthermore, qRT-PCR analysis verified 18 randomly chosen genes from those significantly enriched in the KEGG pathway” should be “Furthermore, qRT-PCR analysis verified **15** randomly chosen genes from those significantly enriched in the KEGG pathway.”
2. In Fig. 4, caption (b) “Eighteen DEGs from significant KEGG Pathway Classification Enrichment were randomly selected for qRT-PCR validation” should be “b **Fifteen** DEGs from significant KEGG Pathway Classification Enrichment were randomly selected for qRT-PCR validation.”
3. Fig. 4b was duplicated as Fig. 5b. The correct Fig. 5 is provided in this Correction.

**Fig. 5:**
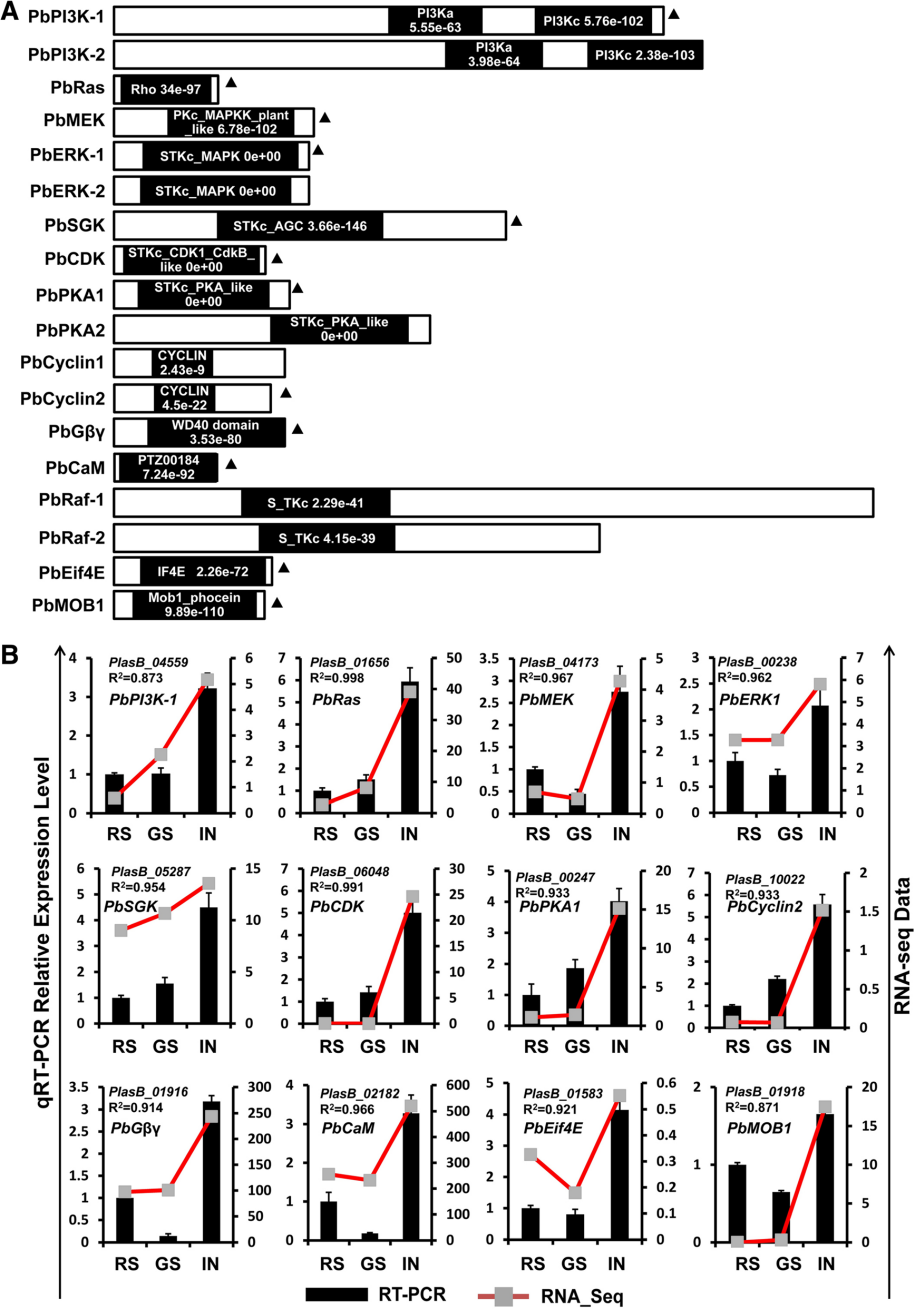
Proteins involved in cancer-related signaling pathways in *P. brassicae* and qRT-PCR validation of the expression pattern. **a** Schematic diagram of proteins encoded by genes of cancer-related signaling pathways in *P. brassicae*. The black frames represented conserved domains in the genes encoded proteins. The information of conserved domain, e-value, and length was obtained from NCBI database. **b** Twelve core genes of cancer-related signaling pathways (marked with black solid triangle in (**a**) were chosen for qRT-PCR validation. Expression levels of these 12 genes from the three different samples (RS, GS and IN) were measured by RNA-seq data (Red line chart) and qRT-PCR data (black histogram). The actin gene of *P. brassicae* was used as an internal control to normalize the expression level. Data from qRT-PCR represent the means and standard deviations (three replications). R-value of Pearson’s correlation coefficient was used to measure the consistency of the RNA-seq data and qRT-PCR. See Additional file 3: Table S1 and Additional file 4: Table S2 for genes information
