# Supplementary figures and images for: Proto-oncogenes in a eukaryotic unicellular organism play essential roles in plasmodial growth in host cells
Source: BMC Genomics. 2018 Dec 6;19:881. doi: 10.1186/s12864-018-5307-4 (PMC6282348; doi:10.1186/s12864-018-5307-4)

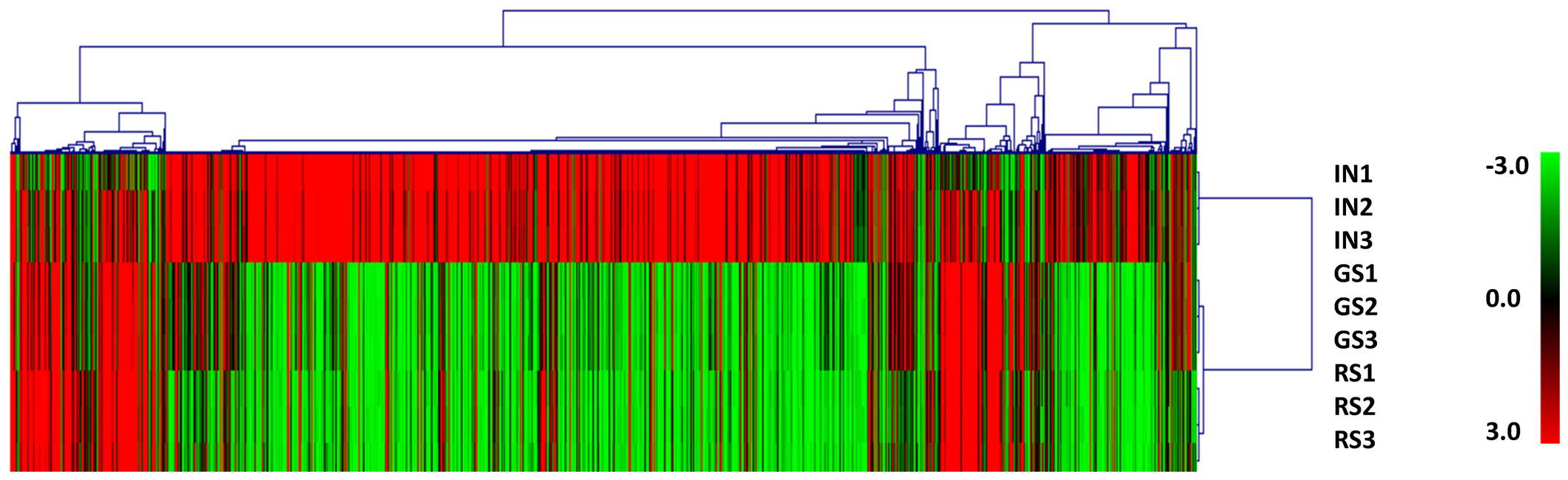

Supplement: Supplementary file 1 — Figure S1. Heatmap of DEGs of P. brassicae at three stages. IN, multinucleate secondary plasmodia stage in plant cortical cells; GS, germinating resting spores stage when the resting spores germinating and releasing primary zoospores; and RS, resting spores stage. (TIF 1974 kb) [file 12864_2018_5307_MOESM1_ESM.tif]

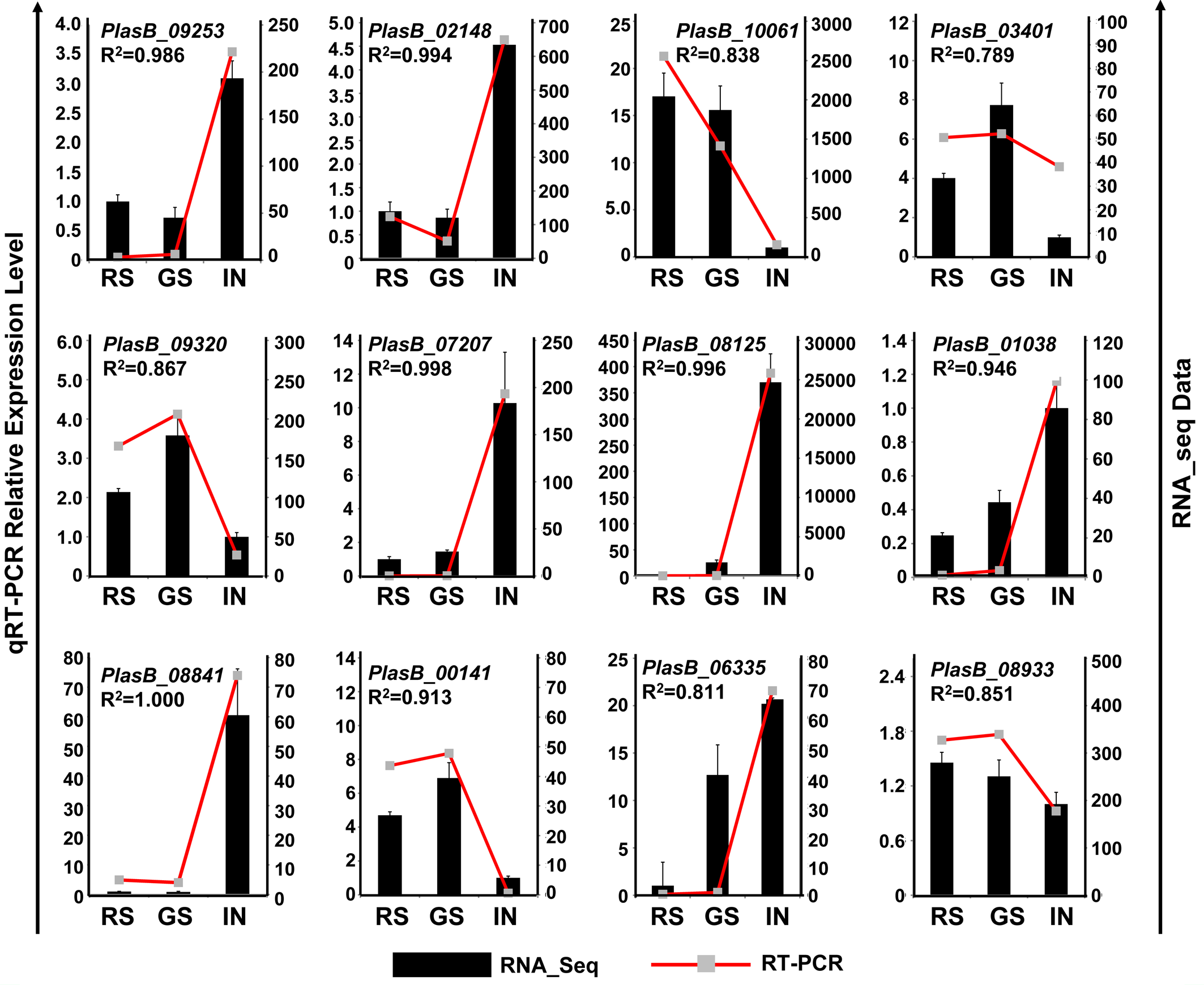

Supplement: Supplementary file 2 — Figure S2. Validation of RNA-seq results of P. brassicae by qRT-PCR. Comparison of expression levels for the randomly selected 12 genes from the three different samples (RS, GS and IN) were measured by RNA-seq data (gray line chart) and qRT-PCR data (black histogram). The P. brassicae actin gene was used as an internal control to normalize the expression level. Data from qRT-PCR represent the means and standard deviations. Pearson’s correlation coefficient (R-value) was used to measure the consistency of the RNA-seq data and qRT-PCR. See Additional file 3: Table S1 for primer information. (TIF 736 kb) [file 12864_2018_5307_MOESM2_ESM.tif]

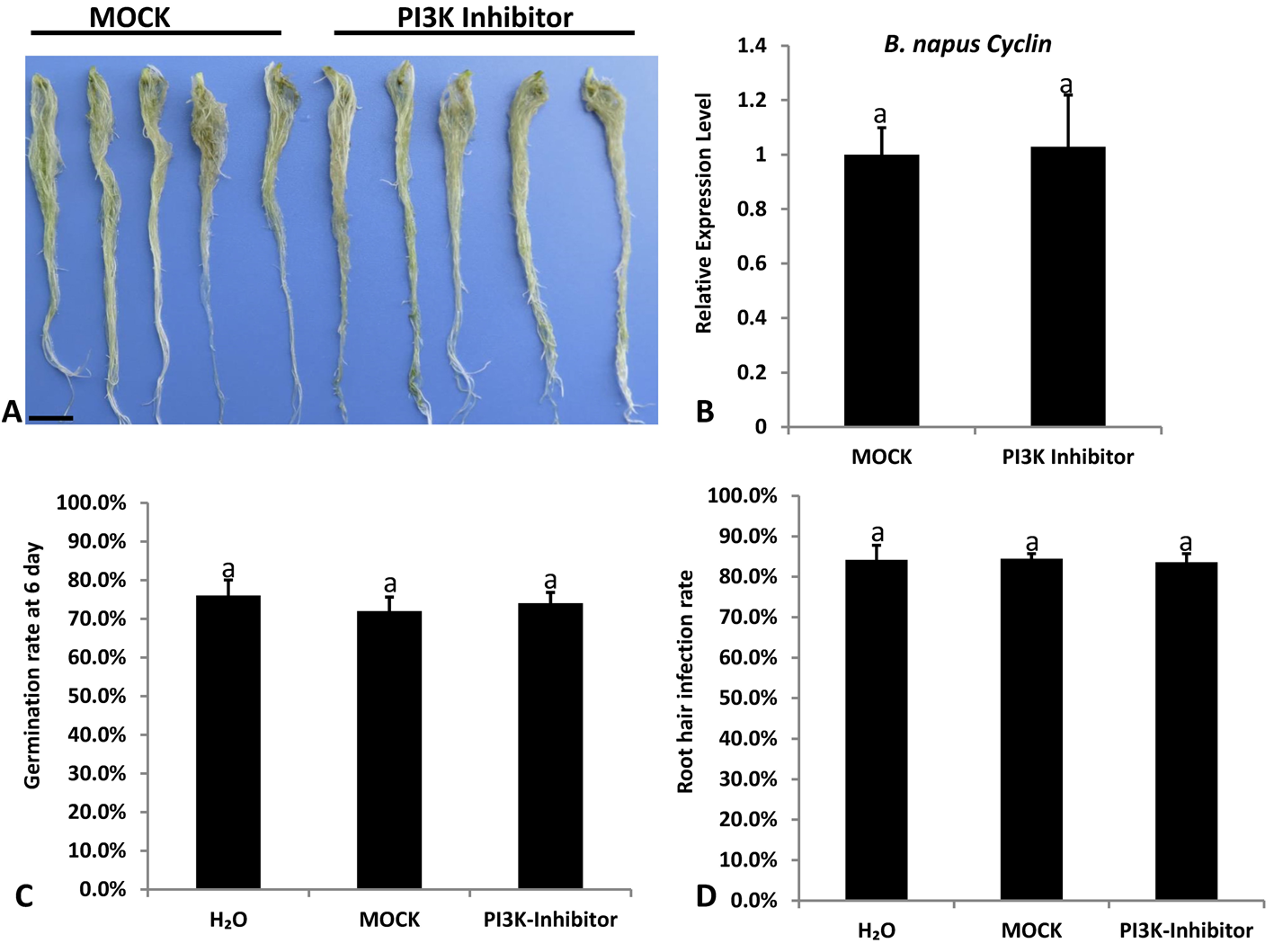

Supplement: Supplementary file 7 — Figure S3. Effect of PI3K inhibitor treatment on the growth, development of oilseed rape plants, resting spores germination rate and root hair infection rate of P. brassicae. a Growth and development status of oilseed rape plants treated with PI3K inhibitor (right). MOCK (DMSO) treatment served as control (left). The pictures of plants were taken at 28 day after treatment. Bar = 1.5 cm. b At 28 day after treatment, the roots of MOCK treated plants and inhibitor treated plants were harvested. The expression level of Cyclin gene (XM_013809141.1, downstream gene of PI3K signaling pathway) in B. napus with MOCK and inhibitor treatment was quantified by qPCR. The actin gene of B. napus was used as control to normalize the expression level. Data represent the means and standard deviations. The expression level of MOCK treated group was set as 1.0. Statistically significant difference of data between MOCK and inhibitor treated groups was compared, same letter in the graph indicates no significant differences at the level of P = 0.05. c-d Resting spores germination rate and root hair infection rate of P. brassicae were compared between H2O, MOCK and PI3K-Inhibitor treated groups. At 6 day, the treated spores were stained with orcein (Sigma-Aldrich Canada). The germination rate of spores was counted under microscope. At 7 dpi, the roots of oilseed rape plants were stained with Trypan Blue, then the root hair infection rate was counted with microscopic examination. The graphic data represent the means and standard deviations from three biological replicates. At the level of P = 0.05, statistically significant differences of data between H2O, MOCK and inhibitor treated groups were compared, same letters in the graph indicate no significant differences. (TIF 642 kb) [file 12864_2018_5307_MOESM7_ESM.tif]
