# Supplementary material for: Proto-oncogenes in a eukaryotic unicellular organism play essential roles in plasmodial growth in host cells
Source: BMC Genomics. 2018 Dec 6;19:881. doi: 10.1186/s12864-018-5307-4 (PMC6282348; doi:10.1186/s12864-018-5307-4)
Supplement: Supplementary file 3 — Table S1. Genes examined and primer pairs used in this study. (DOCX 38 kb) [file 12864_2018_5307_MOESM3_ESM.docx]

**Additional file 3: Table S1** Genes examined and primer pairs used in this study

| **1. Prime pairs for qRT-PCR verification of RNA_seq data and corresponding expression patterns extracted from RNA_Seq** | | | | | | | | | | | | | |  |
| --- | --- | --- | --- | --- | --- | --- | --- | --- | --- | --- | --- | --- | --- | --- |
| Gene ID | | Primers sequences (5’–3’) | | | | | | Expression patterns extracted by RNA-Seq | | | | | |  |
|  |  |  |  |  |  |  |  | RS | | GS | | | IN |  |
| PlasB_09253 | | F | | CGACCTGACACACGATGAG | | | | 3.336341 | | 6.36628215 | | | 221.3985225 |  |
|  |  | R | | GTCTTGGCGGATTGCTTCT | | | |  |  |  |  |  |  |  |
| PlasB_02148 | | F | | GAACGACATTGACCAGAT | | | | 123.6666 | | 51.1435861 | | | 649.1667885 |  |
|  |  | R | | CATGCAGTAGAGGAACAG | | | |  |  |  |  |  |  |  |
| PlasB_10061 | | F | | GACACGGACGAGGACGAC | | | | 2555.326 | | 1412.98197 | | | 155.8953204 |  |
|  |  | R | | TTACGACGACGCACGACTT | | | |  |  |  |  |  |  |  |
| PlasB_03401 | | F | | AACGGCTCTCGGACAATA | | | | 50.65843 | | 52.2881955 | | | 38.24677393 |  |
|  |  | R | | CAGTCGGCGAATCTCTTC | | | |  |  |  |  |  |  |  |
| PlasB_09320 | | F | | GCATGTTCTGTGGTCTTACG | | | | 145.4766 | | 186.124134 | | | 6.833455568 |  |
|  |  | R | | AGCCTGTCTGAATGGTCTC | | | |  |  |  |  |  |  |  |
| PlasB_07207 | | F | | GTTCGCCTCCAGTATCAG | | | | 0.131193 | | 0.31024981 | | | 193.4415115 |  |
|  |  | R | | TGTCCAATCGTTCCAATGT | | | |  |  |  |  |  |  |  |
| PlasB_08125 | | F | | TGGAGGACTCGGATTGGA | | | | 32.51888 | | 58.0791292 | | | 25836.70695 |  |
|  |  | R | | AACGCATTCGCCATATTCC | | | |  |  |  |  |  |  |  |
| PlasB_01038 | | F | | GAGAACATTAGCCTACAATCCA | | | | 1.163385 | | 3.4808193 | | | 99.30486778 |  |
|  |  | R | | TGACGAACTGCGATACAC | | | |  |  |  |  |  |  |  |
| PlasB_08841 | | F | | AACAATTCAGGAAGGATAT | | | | 4.912173 | | 4.01112898 | | | 73.9403328 |  |
|  |  | R | | CATCGTAATCGGTAAGAT | | | |  |  |  |  |  |  |  |
| PlasB_00141 | | F | | CAACAACAGTGGCGTCTTC | | | | 43.6038 | | 47.7679039 | | | 0.55219843 |  |
|  |  | R | | TACTTCATCTCGTGCGTGTAG | | | |  |  |  |  |  |  |  |
| PlasB_06335 | | F | | ACCTATCTGATGACATTG | | | | 0.338617 | | 0.97964249 | | | 69.02589729 |  |
|  |  | R | | CGATTAACATCTCCTTCA | | | |  |  |  |  |  |  |  |
| PlasB_08933 | | F | | CGTCTCCGTCCTCTTCAC | | | | 277.2001 | | 289.424552 | | | 127.6958869 |  |
|  |  | R | | AAGCCCGACATCCACTTC | | | |  |  |  |  |  |  |  |
| **2. Prime pairs used for qRT-PCR validation of KEGG pathway classification enriched DGEs and corresponding expression patterns extracted from RNA_Seq** | | | | | | | | | | | | | |  |
| Gene ID | Putative function | | Pathway | | Primers sequences (5’–3’) | | | | Expression patterns extracted by RNA-Seq | | | | |  |
|  |  |  |  |  |  |  |  |  | RS | | GS | IN | |  |
| PlasB_04037 | NOP4, RBM28; nucleolar protein 4 | | Translation (ko03008) | | F | CTGGCTGATGGTCTGACA | | | 0.538073611 | | 0.38314417 | 82.05566017 | |  |
|  |  |  |  |  | R | GGCTTGGAATCTGGACAATG | | |  |  |  |  |  |  |
| PlasB_03813 | NUG1, GNL3; nuclear GTP-binding protein | | Translation (ko03008) | | F | CGGATGACGCCACAATCA | | | 0.338617345 | | 0.979642488 | 69.02589729 | |  |
|  |  |  |  |  | R | GCCAGACAGCCAGATACG | | |  |  |  |  |  |  |
| PlasB_08841 | PRI1; DNA primase small subunit [EC:2.7.7.-] | | Replication and repair (ko03030) | | F | AACAATTCAGGAAGGATAT | | | 4.912173193 | | 4.011128983 | 73.9403328 | |  |
|  |  |  |  |  | R | CATCGTAATCGGTAAGAT | | |  |  |  |  |  |  |
| PlasB_01038 | MCM5, CDC46; DNA replication licensing factor MCM5 [EC:3.6.4.12] | | DNA replication, ko03030; Cell cycle, ko04110 | | F | GAGAACATTAGCCTACAATCCA | | | 1.163385259 | | 3.480819301 | 99.30486778 | |  |
|  |  |  |  |  | R | TGACGAACTGCGATACAC | | |  |  |  |  |  |  |
| PlasB_01775 | STX1B_2_3; syntaxin 1B/2/3 | | Folding, sorting and degradation (ko04130) | | F | GAGAAGCACGCCAGGAAT | | | 0.059086157 | | 0.571204546 | 39.51777473 | |  |
|  |  |  |  |  | R | GATGACCGAACAGACGAGTA | | |  |  |  |  |  |  |
| PlasB_07616 | ASAP; Arf-GAP with SH3 domain, ANK repeat and PH domain-containing protein | | Transport and catabolism (ko04144; ko04666) | | F | GACGCACATCAGCAAAGT | | | 0.226007579 | | 0.576901872 | 35.63417809 | |  |
|  |  |  |  |  | R | GCCGACAGCCTTAATGAAC | | |  |  |  |  |  |  |
| PlasB_09043 | PRDX5; peroxiredoxin 5, atypical 2-Cys peroxiredoxin [EC:1.11.1.15] | | Transport and catabolism (ko04146) | | F | TACAGCATGTTCGTCAAT | | | 3.991189713 | | 9.686603017 | 243.0036002 | |  |
|  |  |  |  |  | R | GTCAGGTACTTCAGCATC | | |  |  |  |  |  |  |
| PlasB_04269 | YCS4, CNAP1, CAPD2; condensin complex subunit 1 | | Cell growth and death (ko04111) | | F | TCCTTGACGATGTGATGG | | | 0.88648475 | | 2.040896321 | 45.03023545 | |  |
|  |  |  |  |  | R | GCGAATGGATTGTGTTGAA | | |  |  |  |  |  |  |
| PlasB_02154 | ORC6; origin recognition complex subunit 6 | | Cell growth and death (ko04110) | | F | GAGATTGAAGAAGTATGT | | | 8.083200138 | | 4.991045042 | 66.39580436 | |  |
|  |  |  |  |  | R | TATATTGTTGTCTGGAAG | | |  |  |  |  |  |  |
| PlasB_07915 | BRRN1, BRN1, CAPH; condensin complex subunit 2 | | Cell growth and death (ko04111) | | F | TCCTTCTGACGACGATTATGA | | | 0.203741769 | | 0.743101741 | 76.93424402 | |  |
|  |  |  |  |  | R | AACCTTCTCCGCCATTCTA | | |  |  |  |  |  |  |
| PlasB_07777 | cyclin E | | Cancers (ko05200; ko05203) | | F | CAAGGCTCTATGTCAAGT | | | 0.964308016 | | 1.674814292 | 58.98551031 | |  |
|  |  |  |  |  | R | CGTCGAACACATGGAATA | | |  |  |  |  |  |  |
| PlasB_03111 | RAD51; DNA repair protein RAD51 | | Cancers (ko05200; ko05212) | | F | TTACATTGACACGGAAGG | | | 0.013723241 | | 0.01600189 | 0.078769516 | |  |
|  |  |  |  |  | R | GAGCACATCGGAAGAATC | | |  |  |  |  |  |  |
| PlasB_03348 | glsA, GLS; glutaminase [EC:3.5.1.2] | | Cancers (ko05206) | | F | CGATTACAGTGGCGAATGG | | | 1.673772706 | | 1.992854507 | 157.0588685 | |  |
|  |  |  |  |  | R | CAGGTTAGGAATGACGAGATAGA | | |  |  |  |  |  |  |
| PlasB_03558 | KIF23; kinesin family member 23 | | Cancers (ko05206) | | F | AGATACCGATGGAATAATC | | | 0.463811642 | | 0.816864865 | 55.34879349 | |  |
|  |  |  |  |  | R | ACGACTTGTTGATGTTAT | | |  |  |  |  |  |  |
| PlasB_04173 | mitogen-activated protein kinase kinase 1 | | Cancers (ko04368) | | F | GCCACCAAATACGCGAGCGA | | | 0.696590395 | | 0.475914853 | 4.27547788 | |  |
|  |  |  |  |  | R | GGTTCATTCCACAGGCGAATT | | |  |  |  |  |  |  |
| **3. Primer pairs used for qRT-PCR validation of cancer-related signaling pathways** | | | | | | | | | | | | | |  |
| Gene ID | Putative function | | Pathway | | Primers sequences (5’–3’) | | | | Expression patterns extracted by RNA-Seq | | | | |  |
|  |  |  |  |  |  |  |  |  | RS | | GS | IN | |  |
| PlasB_04559 | phosphatidylinositol-4,5-bisphosphate 3-kinase (PI3K) | | Regulation of actin cytoskeleton K00922 | | F | CGGCAAGAATTACAAGAA | | | 0.579355326 | | 2.265106731 | 5.172347804 | |  |
|  |  |  |  |  | R | TGAACAGATTGATGAACAG | | |  |  |  |  |  |  |
| PlasB_01656 | Ras homolog gene family, member A (Rho) | | Regulation of actin cytoskeleton K04513 | | F | TGGTCCTGATCGTATTCTC | | | 2.622983908 | | 8.098670945 | 39.08684899 | |  |
|  |  |  |  |  | R | AGTGGTGTTGGATCTCTG | | |  |  |  |  |  |  |
| PlasB_04173 | mitogen-activated protein kinase kinase 1 (MEK) | | Cell proliferation K04368 | | F | TGTCAGTGGCAAGGTATC | | | 0.696590395 | | 0.475914853 | 4.27547788 | |  |
|  |  |  |  |  | R | CGCATTCCATTAAGGTGAT | | |  |  |  |  |  |  |
| PlasB_00238 | mitogen-activated protein kinase 1/3 (ERK) | | Cell proliferation K04371 | | F | GGCGACAACTACATTCAC | | | 3.278214027 | | 3.281775547 | 5.807515242 | |  |
|  |  |  |  |  | R | GCTTCTTCTTCAGGTTCTTAA | | |  |  |  |  |  |  |
| PlasB_05287 | serum/glucocorticoid-regulated kinase 1 (SGK) | | Survival signal, Growth and proliferation K13302 | | F | CTAAGCAACTGTTCGCATT | | | 9.013803864 | | 10.66204041 | 13.58039689 | |  |
|  |  |  |  |  | R | GCCGTATGTACTCCAAGAT | | |  |  |  |  |  |  |
| PlasB_06048 | cyclin-dependent kinase 2 (CDK) | | Cell cycle progression K02206 | | F | AAGATGCTCCGCTATGAAC | | | 0.0603498 | | 0.031213401 | 24.64345486 | |  |
|  |  |  |  |  | R | AGTCGTACAGGTCGTCAA | | |  |  |  |  |  |  |
| PlasB_10022 | cyclin D2 | | Cell cycle progression K10151 | | F | AACCTGATCGACGAGTTC | | | 0.074174078 | | 0.06793432 | 1.518545682 | |  |
|  |  |  |  |  | R | GTACGAGAACTGCCTGTA | | |  |  |  |  |  |  |
| PlasB_01583 | translation initiation factor 4E | | Protein synthesis K03259 | | F | CATAAAGGACAAGGTCGC | | | 0.326567376 | | 0.179457298 | 0.55219843 | |  |
|  |  |  |  |  | R | GAACAATGTTGCTGGAAT | | |  |  |  |  |  |  |
| PlasB_01918 | MOB kinase activator 1 | | Protein synthesis K06685 | | F | ACCTCAACACCTGCTTCA | | | 0 | | 0.306263262 | 17.49546163 | |  |
|  |  |  |  |  | R | GCTCCTGCTCCTTCTTGT | | |  |  |  |  |  |  |
| PlasB_00247 | protein kinase A (PKA1) | | The downstream regulator of GPCRs involved in cancer related signaling pathway K04345 | | F | GTTGAAGATGCTGAAGAAGAC | | | 79.24259328 | | 112.6833627 | 177.5263531 | |  |
|  |  |  |  |  | R | CGCCGATGACATATTCCA | | |  |  |  |  |  |  |
| PlasB_01916 | guanine nucleotide-binding protein G(I)/G(S)/G(T) subunit beta-1 (Gβγ) | | The downstream regulator of GPCRs involved in cancer related signaling pathway K04536 | | F | TGCTGTCGCTTCATCAAC | | | 97.5200582 | | 100.9154585 | 243.8887669 | |  |
|  |  |  |  |  | R | GCTGCTTCTGCTCAATGT | | |  |  |  |  |  |  |
| PlasB_02182 | calmodulin | | The downstream regulator of GPCRs involved in cancer related signaling pathway K02183 | | F | GAGTTCCTGACGATGATG | | | 255.8407324 | | 232.3118166 | 520.8442327 | |  |
|  |  |  |  |  | R | CTTGTCGAACACCTTGAA | | |  |  |  |  |  |  |
| **4. Primer pairs used for qRT-PCR validation of GO enrichment of DGEs and corresponding expression patterns extracted from RNA_Seq data** | | | | | | | | | | | | | |  |
| Gene ID | Putative function | | Pathway | | Primers sequences (5’–3’) | | | | Expression patterns extracted by RNA-Seq | | | | |  |
|  |  |  |  |  |  |  |  |  | RS | | GS | IN | |  |
| PlasB_07290 | F: ATP binding; C: cytoplasm; C: cytoskeleton; F: nucleotide binding | | cell differentiation, growth, reproduction, translation (BP) | | F | TTGAGACCAAAGTTGAAGTTT | | | 8.116317 | | 18.49665 | 234.0456 | |  |
|  |  |  |  |  | R | AATCGTCGCACAATCTGA | | |  |  |  |  |  |  |
| PlasB_01053 | F: microtubule motor activity; P: microtubule-based movement; C: microtubule | |  |  | F | GTAACAATCAGCAGGAAGT | | | 0.14038 | | 0.098827 | 4.842041 | |  |
|  |  |  |  |  | R | TTCATAGGTCGGGTTCAT | | |  |  |  |  |  |  |
| PlasB_02696 | C: myosin complex; F: motor activity; F: protein binding; F: ATP binding | |  | | F | GACTATATCTATACCAACATC | | | 0.002268 | | 0.031158 | 0.371051 | |  |
|  |  |  |  | | R | TTTATACTTGCGAATGAC | | |  |  |  |  |  |  |
| PlasB_09671 | C: membrane; F: protein binding | |  | | F | ACGAGGTCCAACTTATACA | | | 0.015691 | | 0.136814 | 2.590696 | | |
|  |  |  |  | | R | TAGTAGGCGGTCTTGAAG | | |  |  |  |  |  |  |
| PlasB_09800 | C: ribosome; F: structural constituent of ribosome; P: translation; P: ribosome biogenesis | | structural molecule activity (MF) | | F | GAAGAAGGTCGGCATCAC | | | 7.119788 | | 7.520348 | 605.5045 | | |
|  |  |  |  |  | R | TCGATCTTCTTGATCTGCTT | | |  |  |  |  |  |  |
| PlasB_01922 | C: ribosome; F: structural constituent of ribosome; P: translation; P: ribosome biogenesis | |  |  | F | CGCTTCTCTACAATATGC | | | 23.42664 | | 19.15345 | 662.8945 | | |
|  |  |  |  |  | R | CTTAACAACCTTGACCTT | | |  |  |  |  |  |  |
| PlasB_07507 | C:ribosome; F:structural constituent of ribosome; P:translation; P:ribosome biogenesis | |  | | F | GTCCTCAAACGCACCAAG | | | 58.53769 | | 85.99085 | 1563.921 | | |
|  |  |  |  | | R | TTAGAAGACAACATCACCCTT | | |  |  |  |  |  |  |
| PlasB_01791 | C: ribosome; F: structural constituent of ribosome; P: translation; P: ribosome biogenesis | |  | | F | AAGTTGAAGCGTGTGAAG | | | 29.97068 | | 28.8938 | 1047.877 | | |
|  |  |  |  | | R | CAGTTCTTGACCGTGTTC | | |  |  |  |  |  |  |
| PlasB_03590 | C: ribosome; F: RNA binding; F: structural constituent of ribosome; P: translation; P: ribosome biogenesis | |  | | F | ATTCTGCGTATCCTGAACA | | | 40.58769 | | 32.09557 | 2860.731 | | |
|  |  |  |  | | R | GACACCCTTGATCTTCGT | | |  |  |  |  |  |  |
| PlasB_02130 | F: hydrolase activity, hydrolyzing O-glycosyl compounds; P: carbohydrate metabolic process | | external encapsulating structure (CC) | | F | GCGTTTCTTGCTTTATGG | | | 0.212092 | | 0.329755 | 6.259007 | | |
|  |  |  |  |  | R | ATCTCGTCCTCATTGTTG | | |  |  |  |  |  |  |
| PlasB_09605 | C: ribosome; F: structural constituent of ribosome; P: translation; P: ribosome biogenesis | | cell (CC) | | F | TATGGCGTGTCGTTTAAG | | | 24.58261 | | 19.19904 | 1913.764 | | |
|  |  |  |  |  | R | TCTTCATCATCTGCTTGG | | |  |  |  |  |  |  |
| PlasB_02486 | F: protein binding | |  |  | F | CTGGCTGTTGCTGAATAC | | | 2.100519 | | 1.379209 | 348.6228 | | |
|  |  |  |  |  | R | TAAAGCGGAAGATGTTGTC | | |  |  |  |  |  |  |
| PlasB_01173 | C: nucleosome; F: DNA binding; F: protein heterodimerization activity | | intracellular (CC) | | F | ATGGAGATTCGCAAGTTC | | | 2.056452 | | 1.491597 | 90.22453 | | |
|  |  |  |  |  | R | AAGTGTTCCTGTGCTATT | | |  |  |  |  |  |  |
| PlasB_03329 | F: ATP binding; C: cytoplasm; C: cytoskeleton; F :nucleotide binding; C: plasmodesma; C: mitochondrion; C: chloroplast envelope; C: chloroplast stroma; C: plasma membrane | | organelle (CC) | | F | CGAGAAGATGACGCAGAT | | | 3.026283 | | 2.231275 | 483.0682 | | |
|  |  |  |  |  | R | GACAGAACGGCCTGAATG | | |  |  |  |  |  |  |
| PlasB_07163 | C: ribosome; F: structural constituent of ribosome; P: translation; P: ribosome biogenesis | |  | | F | TGTCGTCAAGTCGGTCAA | | | 51.6168 | | 54.13476 | 734.6556 | | |
|  |  |  |  | | R | ATGTTCTTGTGTCGCTTCTC | | |  |  |  |  |  |  |
| **5. Other primer pairs** | | | | | | | | | | | | | | |
| Gene | Targets | | Sense-primer (5’–3’) | | | | Antisense-primer (5’–3’) | | | | | | | |
| *PB-Actin* | *Actin* of *Plasmodiophora brassicae* | | CACCGACTACCTGATGAA | | | | CAGCTTCTCCTTGATGTC | | | | | | | |
| *Bn-Actin* | *Actin* of *Brassicae napus* | | AACTCAAACCTCTCTACC | | | | TAGTTGTTCTTCACCATC | | | | | | | |
| *Bn-Cyclin* | *Cyclin* of *B. napus* | | CGATTTACCGACGCGCCAACGGCTA | | | | TTCTCTACAACTCGATTGCTTTTCG | | | | | | | |
