# Supplementary material for: Proto-oncogenes in a eukaryotic unicellular organism play essential roles in plasmodial growth in host cells
Source: BMC Genomics. 2018 Dec 6;19:881. doi: 10.1186/s12864-018-5307-4 (PMC6282348; doi:10.1186/s12864-018-5307-4)
Supplement: Supplementary file 4 — Table S2. The genes involve in Cancers related pathways on the genome of P. brassicae and corresponding expression pattern. (DOCX 32 kb) [file 12864_2018_5307_MOESM4_ESM.docx]

**Additional file 4: Table S2** The genes involve in Cancers related pathways on the genome of *P. brassicae* and corresponding expression pattern

| **NO** | **SeqID** | **KO** | **Expression pattern extracted by RNA-Seq** | | |
| --- | --- | --- | --- | --- | --- |
|  |  |  | **RS** | **GS** | **IN** |
| 1 | PlasB_08792 | K03083 | 53.25536 | 53.25536 | 47.93906 |
| 2 | PlasB_01956 | K07532 | 21.90203 | 21.90203 | 11.67965 |
| 3 | PlasB_01656 | K04513 | 2.622984 | 2.622984 | 39.08685 |
| 4 | PlasB_00247 | K04345 | 1.098882 | 1.098882 | 15.14415 |
| 5 | PlasB_07097 | K04345 | 79.24259 | 79.24259 | 177.5264 |
| 6 | PlasB_01916 | K04536 | 97.52006 | 97.52006 | 243.8888 |
| 7 | PlasB_06301 | K05637 | 24.12601 | 24.12601 | 24.63602 |
| 8 | PlasB_08057 | K06240 | 0.500178 | 0.500178 | 31.38412 |
| 9 | PlasB_04559 | K00922 | 0.579355 | 0.579355 | 5.172348 |
| 10 | PlasB_04589 | K00922 | 9.93288 | 9.93288 | 17.45487 |
| 11 | PlasB_05276 | K01110 | 2.664306 | 2.664306 | 46.82094 |
| 12 | PlasB_09097 | K01110 | 48.91157 | 48.91157 | 50.17219 |
| 13 | PlasB_03025 | K01110 | 8.288052 | 8.288052 | 11.20362 |
| 14 | PlasB_04446 | K07203 | 21.253 | 21.253 | 20.60089 |
| 15 | PlasB_02239 | K12362 | 2.682299 | 2.682299 | 8.408564 |
| 16 | PlasB_04195 | K03099 | 22.52758 | 22.52758 | 46.9838 |
| 17 | PlasB_06593 | K04365 | 0.353293 | 0.353293 | 7.430616 |
| 18 | PlasB_07093 | K04365 | 1.622239 | 1.622239 | 0.444364 |
| 19 | PlasB_09434 | K04366 | 407.4392 | 407.4392 | 1955.896 |
| 20 | PlasB_04173 | K04368 | 0.69659 | 0.69659 | 4.275478 |
| 21 | PlasB_00238 | K04371 | 3.278214 | 3.278214 | 5.807515 |
| 22 | PlasB_02211 | K04371 | 0.76384 | 0.76384 | 0.172562 |
| 23 | PlasB_08852 | K09291 | 149.5412 | 149.5412 | 81.45826 |
| 24 | PlasB_05498 | K04392 | 188.1425 | 188.1425 | 112.6861 |
| 25 | PlasB_05503 | K04392 | 142.7082 | 142.7082 | 245.5336 |
| 26 | PlasB_07898 | K06620 | 75.70414 | 75.70414 | 31.16454 |
| 27 | PlasB_10887 | K06620 | 1.069884 | 1.069884 | 1.637147 |
| 28 | PlasB_01371 | K02219 | 17.28348 | 17.28348 | 233.6962 |
| 29 | PlasB_03214 | K02206 | 52.44463 | 52.44463 | 208.9915 |
| 30 | PlasB_07777 | K06626 | 0.964308 | 0.964308 | 58.98551 |
| 31 | PlasB_00802 | K06067 | 199.4193 | 199.4193 | 80.69225 |
| 32 | PlasB_00888 | K06067 | 276.0198 | 276.0198 | 153.3181 |
| 33 | PlasB_09252 | K08734 | 109.1984 | 109.1984 | 76.91343 |
| 34 | PlasB_00357 | K08735 | 5.588566 | 5.588566 | 33.56085 |
| 35 | PlasB_06234 | K08737 | 25.51749 | 25.51749 | 50.18812 |
| 36 | PlasB_04779 | K04482 | 0.246203 | 0.246203 | 0.124623 |
| 37 | PlasB_10828 | K04482 | 0.032077 | 0.032077 | 0.299783 |
| 38 | PlasB_03111 | K04482 | 15.95562 | 15.95562 | 148.8111 |
| 39 | PlasB_10375 | K08738 | 318.1768 | 318.1768 | 23.29002 |
| 40 | PlasB_03162 | K08738 | 0.031749 | 0.031749 | 0.047248 |
| 41 | PlasB_03164 | K08738 | 16.54528 | 16.54528 | 62.64791 |
| 42 | PlasB_05830 | K03872 | 108.4126 | 108.4126 | 42.95698 |
| 43 | PlasB_10370 | K03872 | 6.637435 | 6.637435 | 3.520265 |
| 44 | PlasB_05973 | K03868 | 192.1374 | 192.1374 | 129.6596 |
| 45 | PlasB_03989 | K03868 | 22.21037 | 22.21037 | 13.0802 |
| 46 | PlasB_07547 | K09592 | 0.337233 | 0.337233 | 6.453819 |
| 47 | PlasB_09054 | K09592 | 26.50244 | 26.50244 | 62.47331 |
| 48 | PlasB_01001 | K01679 | 28.47027 | 28.47027 | 54.79422 |
| 49 | PlasB_09108 | K06228 | 19.68986 | 19.68986 | 24.63032 |
| 50 | PlasB_01119 | K04079 | 5619.024 | 5619.024 | 2974.053 |
| 51 | PlasB_09853 | K04079 | 249.5969 | 249.5969 | 665.0629 |
| 52 | PlasB_00176 | K00873 | 226.2204 | 226.2204 | 180.6233 |
| 53 | PlasB_06730 | K00873 | 122.7941 | 122.7941 | 190.7554 |
| 54 | PlasB_03906 | K00161 | 368.3108 | 368.3108 | 293.5687 |
| 55 | PlasB_01635 | K00162 | 39.35622 | 39.35622 | 48.215 |
| 56 | PlasB_04314 | K11416 | 60.7019 | 60.7019 | 45.4881 |
| 57 | PlasB_03348 | K01425 | 1.673773 | 1.673773 | 157.0589 |
| 58 | PlasB_03941 | K00036 | 147.2978 | 147.2978 | 93.80516 |
| 59 | PlasB_04519 | K00850 | 68.60774 | 68.60774 | 114.1725 |
| 60 | PlasB_02019 | K00850 | 50.47537 | 50.47537 | 64.14432 |
| 61 | PlasB_01286 | K07207 | 28.2287 | 28.2287 | 30.074 |
| 62 | PlasB_05289 | K07208 | 51.88327 | 51.88327 | 57.30064 |
| 63 | PlasB_00676 | K04688 | 33.90371 | 33.90371 | 24.6664 |
| 64 | PlasB_04163 | K00889 | 29.76926 | 29.76926 | 25.78629 |
| 65 | PlasB_00759 | K00889 | 51.53183 | 51.53183 | 37.60162 |
| 66 | PlasB_02955 | K01115 | 45.51804 | 45.51804 | 50.33317 |
| 67 | PlasB_00793 | K15377 | 13.70308 | 13.70308 | 14.4347 |
| 68 | PlasB_08560 | K14156 | 25.1385 | 25.1385 | 14.97188 |
| 69 | PlasB_08629 | K00968 | 602.1318 | 602.1318 | 235.2626 |
| 70 | PlasB_01378 | K00994 | 49.33069 | 49.33069 | 51.23286 |
| 71 | PlasB_05668 | K18695 | 26.14933 | 26.14933 | 35.74393 |
| 72 | PlasB_00135 | K11644 | 151.9517 | 151.9517 | 69.3764 |
| 73 | PlasB_05988 | K04650 | 0.627241 | 0.627241 | 30.85409 |
| 74 | PlasB_00362 | K15188 | 27.09517 | 27.09517 | 43.78437 |
| 75 | PlasB_08910 | K15188 | 80.70869 | 80.70869 | 52.48541 |
| 76 | PlasB_01313 | K00069 | 104.9674 | 318.7164 | 1667.187 |
| 77 | PlasB_10022 | K10151 | 49.38709 | 49.38709 | 7.780731 |
| 78 | PlasB_04274 | K11424 | 125.4968 | 125.4968 | 96.44377 |
| 79 | PlasB_05469 | K11253 | 8.143503 | 8.143503 | 56.15463 |
| 80 | PlasB_09232 | K11253 | 6466.489 | 6466.489 | 1978.465 |
| 81 | PlasB_06556 | K12823 | 157.928 | 157.928 | 561.3115 |
| 82 | PlasB_06886 | K12823 | 1744.935 | 1744.935 | 683.0198 |
| 83 | PlasB_04501 | K13209 | 124.734 | 124.734 | 194.7556 |
| 84 | PlasB_03409 | K04728 | 13.75847 | 13.75847 | 14.19241 |
| 85 | PlasB_03169 | K05866 | 2.228814 | 2.228814 | 20.80622 |
| 86 | PlasB_05610 | K07204 | 73.62612 | 73.62612 | 54.93189 |
| 87 | PlasB_07294 | K02599 | 10.35064 | 10.35064 | 34.82895 |
| 88 | PlasB_09717 | K10577 | 1125.688 | 1125.688 | 213.478 |
| 89 | PlasB_02759 | K06252 | 0.76136 | 0.76136 | 6.97667 |
| 90 | PlasB_06658 | K05658 | 40.94918 | 40.94918 | 37.84504 |
| 91 | PlasB_01710 | K05658 | 1.817302 | 1.817302 | 14.23231 |
| 92 | PlasB_09366 | K05658 | 0.494179 | 0.494179 | 17.69801 |
| 93 | PlasB_09614 | K05658 | 15.11819 | 15.11819 | 12.03967 |
| 94 | PlasB_09945 | K05665 | 63.53828 | 63.53828 | 23.82079 |
| 95 | PlasB_07575 | K10605 | 1.406892 | 1.406892 | 2.450381 |
| 96 | PlasB_10736 | K10605 | 718.5526 | 718.5526 | 91.49238 |
| 97 | PlasB_08359 | K12035 | 0.020693 | 0.020693 | 0.588975 |
| 98 | PlasB_09670 | K12035 | 0 | 0 | 0.066807 |
| 99 | PlasB_09669 | K12035 | 0.013723 | 0.013723 | 0.07877 |
| 100 | PlasB_04757 | K11430 | 76.22568 | 76.22568 | 73.78064 |
| 101 | PlasB_01442 | K11411 | 25.50169 | 25.50169 | 21.63066 |
| 102 | PlasB_03558 | K17387 | 0.463812 | 0.463812 | 55.34879 |
| 103 | PlasB_04390 | K05692 | 470.3079 | 470.3079 | 2144.093 |
| 104 | PlasB_07001 | K05692 | 0.06808 | 0.06808 | 0.031591 |
| 105 | PlasB_07005 | K05692 | 235.318 | 235.318 | 31.47531 |
| 106 | PlasB_02084 | K05692 | 5.523257 | 5.523257 | 66.01915 |
| 107 | PlasB_02796 | K05692 | 1.366416 | 1.366416 | 89.90671 |
| 108 | PlasB_03329 | K05692 | 3.026283 | 3.026283 | 483.0682 |
| 109 | PlasB_05161 | K10380 | 5.733423 | 5.733423 | 6.396879 |
| 110 | PlasB_05764 | K10380 | 0.111072 | 0.111072 | 1.310925 |
| 111 | PlasB_02992 | K06269 | 579.5518 | 579.5518 | 283.3722 |
| 112 | PlasB_04902 | K03685 | 4.45456 | 4.45456 | 24.1891 |
| 113 | PlasB_03258 | K03258 | 100.9981 | 100.9981 | 113.5474 |
| 114 | PlasB_02679 | K02991 | 0.042942 | 0.042942 | 0.034512 |
| 115 | PlasB_08139 | K05760 | 3.94845 | 3.94845 | 2.40096 |
| 116 | PlasB_05170 | K01365 | 0.170886 | 0.170886 | 3.56768 |
| 117 | PlasB_09253 | K01365 | 3.336341 | 3.336341 | 221.3985 |
| 118 | PlasB_06583 | K17691 | 58.40122 | 58.40122 | 37.48497 |
| 119 | PlasB_01865 | K01253 | 0.222743 | 0.222743 | 5.597919 |
| 120 | PlasB_08001 | K00799 | 1.551734 | 1.551734 | 462.9701 |
| 121 | PlasB_01793 | K00799 | 0.822357 | 0.822357 | 42.6002 |
| 122 | PlasB_02148 | K00799 | 123.6666 | 123.6666 | 649.1668 |
| 123 | PlasB_03699 | K00799 | 0.949675 | 0.949675 | 18.22725 |
| 124 | PlasB_03586 | K00799 | 111.7948 | 111.7948 | 74.42709 |
| 125 | PlasB_03858 | K00799 | 2.261216 | 2.261216 | 2.998871 |
| 126 | PlasB_05872 | K13299 | 0.521473 | 0.521473 | 3.018955 |
| 127 | PlasB_06682 | K00121 | 144.5902 | 144.5902 | 183.9466 |
| 128 | PlasB_09283 | K00816 | 55.26244 | 55.26244 | 19.10302 |
| 129 | PlasB_06724 | K10610 | 38.71709 | 38.71709 | 58.64642 |
| 130 | PlasB_01784 | K06630 | 1608.886 | 1608.886 | 878.7672 |
| 131 | PlasB_09706 | K06630 | 616.605 | 616.605 | 859.1001 |
| 132 | PlasB_06044 | K11594 | 97.75045 | 97.75045 | 171.0174 |
| 133 | PlasB_02393 | K11594 | 314.9261 | 314.9261 | 269.2657 |
| 134 | PlasB_10047 | K16195 | 8.59346 | 8.59346 | 139.9492 |
| 135 | PlasB_04639 | K11252 | 214.5676 | 214.5676 | 237.5714 |
| 136 | PlasB_06445 | K11252 | 57.17863 | 57.17863 | 171.8825 |
| 137 | PlasB_05470 | K11254 | 1.423441 | 1.423441 | 29.4172 |
| 138 | PlasB_07900 | K11254 | 173.7114 | 173.7114 | 62.89104 |
| 139 | PlasB_02821 | K11254 | 55.24088 | 55.24088 | 33.07578 |
| 140 | PlasB_02998 | K11254 | 339.6944 | 339.6944 | 206.8305 |
| 141 | PlasB_01666 | K03120 | 75.23673 | 75.23673 | 76.81177 |
| 142 | PlasB_04324 | K11838 | 167.6366 | 167.6366 | 68.31062 |
| 143 | PlasB_02475 | K06063 | 136.5446 | 136.5446 | 81.93983 |
| 144 | PlasB_04478 | K03141 | 30.61085 | 30.61085 | 82.25847 |
| 145 | PlasB_08164 | K03142 | 56.62323 | 56.62323 | 68.99567 |
| 146 | PlasB_03778 | K03143 | 117.5441 | 117.5441 | 81.05659 |
| 147 | PlasB_04246 | K03144 | 75.40133 | 75.40133 | 64.92823 |
| 148 | PlasB_07328 | K03136 | 545.8407 | 545.8407 | 467.9525 |
| 149 | PlasB_03150 | K03137 | 300.4825 | 300.4825 | 107.8799 |
| 150 | PlasB_08558 | K15979 | 266.5235 | 266.5235 | 332.4196 |
| 151 | PlasB_04248 | K03124 | 141.3505 | 141.3505 | 114.691 |
| 152 | PlasB_09736 | K06627 | 35.57054 | 35.57054 | 116.0868 |
| 153 | PlasB_08130 | K02146 | 24.14282 | 24.14282 | 50.69302 |
| 154 | PlasB_07611 | K10587 | 34.95483 | 34.95483 | 41.68271 |
| 155 | PlasB_06513 | K03062 | 407.7586 | 407.7586 | 133.3531 |
| 156 | PlasB_06674 | K10691 | 44.71538 | 44.71538 | 35.37696 |
| 157 | PlasB_00691 | K11407 | 113.0148 | 113.0148 | 37.87399 |
| 158 | PlasB_07853 | K11407 | 16.31197 | 16.31197 | 67.22839 |
| 159 | PlasB_04456 | K06062 | 35.87364 | 35.87364 | 57.13006 |
| 160 | PlasB_08148 | K06062 | 76.09136 | 76.09136 | 60.79313 |
| 161 | PlasB_00572 | K03122 | 571.3593 | 571.3593 | 142.9223 |
| 162 | PlasB_02632 | K03123 | 107.4808 | 107.4808 | 84.22131 |
| 163 | PlasB_06811 | K05768 | 109.1006 | 109.1006 | 82.75347 |
| 164 | PlasB_06879 | K05699 | 0.040559 | 0.040559 | 29.52265 |
| 165 | PlasB_00218 | K03363 | 0.588308 | 0.588308 | 10.86391 |
| 166 | PlasB_08212 | K03363 | 119.5943 | 119.5943 | 87.82822 |
| 167 | PlasB_05452 | K15305 | 24.38406 | 24.38406 | 30.34531 |
| 168 | PlasB_08132 | K15306 | 427.9016 | 427.9016 | 321.609 |
| 169 | PlasB_02182 | K02183 | 255.8407 | 255.8407 | 520.8442 |
| 170 | PlasB_05611 | K04353 | 228.304 | 228.304 | 184.7823 |
| 171 | PlasB_02805 | K01522 | 7.498504 | 7.498504 | 7.340732 |
