# Supplementary material for: Proto-oncogenes in a eukaryotic unicellular organism play essential roles in plasmodial growth in host cells
Source: BMC Genomics. 2018 Dec 6;19:881. doi: 10.1186/s12864-018-5307-4 (PMC6282348; doi:10.1186/s12864-018-5307-4)
Supplement: Supplementary file 5 — Table S3. The amino acid sequences encoded by genes involved in cancer-related signaling pathways in P. brassicae. (DOCX 26 kb) [file 12864_2018_5307_MOESM5_ESM.docx]

**Additional file 5: Table S3** The amino acid sequences encoded by genes involved in cancer-related signaling pathways in *P. brassicae*

| **Gene ID** | **Amino acid Sequence** |
| --- | --- |
| **>PlasB_04559 (PbPI3K-1)** | MSVKMGDADADDQDAWWENELQAYHETSKDRGDNIFDAHPSVTAAPLSMADSVDGGMKTGGSNSSAESDDDDDEMKSLSNLVDDDNTNNQGDGGTESRRLQRHISIEETVEMIQTPEHVLAALNRMIEEVTRMSNQAFVSDEARHGLAEAKEALEKVATILEDDDRKDAKTDVDVLLNFDHLPWYFDNPGVAAYRARAYQFPHVQYQQQDSHIPRYLTAGVLPKNTPDSLRCNVVVMQNKRPQTLQMTISSTIWGTADSWKTSDACTGHDTAATLMVKIYDQFETRAEAIRILGDPSQNMLKFVGFDEYIHGTETLYCYETIRASLRNRASVDLCLIQRPIPRVLTESAARHPEIYKERVDPAVPILTIDDFTETRATSTMQWDAMTYFPLRSLQQEFRYRVRGIEGVSESVLPRLDSTHTHLRVRVFLFHGTEIIKHSDMTTTDAPRCQSPRWSRWLTPPDGVPHILASNIPREARISILLYGKRSYTKDEDLLSWVNVPLVDEFGHVRSGMMHLKMWTIDTNIKQGHLDALTFVFRGTTVENLTRGANHQPVSVCIEFEQRALSVTAPIVEVYREPDVAKIGEKQLWKKLSKEVRTNLDILIKTDPLYVLTPKDKEIFWTTRHHLLPYPLALPKFLQSVDWTSVDHRNEAHRLLSNWTPPLNPVSALELLDARYADYKVREYAVNCLRVLPDDELQLFLLQLAQSIKNEPYHDSPLTRFLVERALRNPHSIGHSLFWHLKAELHSPFHCERYAMILEEYLFHSGWHAAELRKQYEIVQRLQRISGKVVARKHAKDMTDDEIQAEYLAALHKFNENYLSTNESTQIPLNPKWLVTTLVVEKCRFMSSKMAPLWLVFKNADPLGDNIIIMFKSGDDLRQDILTLQLLKVMDKIWVSEDLDMRLIPYNCVSTGINAQGKGVGLIEIVLNSDTTSGIQLKYGGGALGALKLDPLHKFIADHNKEPDQYESAKDNFIKSCAGYCVATYVLGIGDRHNGNIMLTKDGRLFHIDFGHFLGNFKSKFGIKRERAAFVLTPEMAFVMGGKNYKKAKEFRVFTDLSAQAFTILRNHASLFINLFSLMVSAQMPELMFQEDIHYLRDKFFLQDSEKGAVERLRKEIQKSLNTTYRQFDNLIHNIKHK* |
| **>PlasB_04589 (PbPI3K-2)** | MGDQIADDAATAASPWIEAVNEDGHVYWYNVETQMSTWDNPFVDETVADELESDSLSVFGQFDAHDVSRARGLSDSISGETEFLVDSGTFSAFVTQPVRRRGSVKAPSDGVPVDDSAGCTSVHSVQNGVHSGGADVGSMSSHSERYHRHDYIAIPENANERRTRMSLTTDPWVECREVVSGRTLFYHRVTKEVVFDVSPSAIDSAAIKTANAIDDAAIESGSGAFPSYFRPAVPSSVPPSDAPVSSNAASVPHKYGPSSMMTMKGDVFNEPVGSYTPLIPDALSQLHSIPIVGELLETQSLPWNFSNPMAQHYRAVALAHPLNEHVPPPQAMMYPRYFSTIPIPAQRHIVCTVKLPESGVKSSMSYTKIHCSATDSANDMVWKALKKCDGRNFEPEKCILKAIGTEEYIYGTASVLDFEYIRVCINSHVEIRLCLLYIDDIQARVESVNDEMRVDEESDRYPADDAFDQVAIYNDGDYQEACNTDAVPVERLRVLPIKALTIPLRFQVCDLENIQLCPRFEPLIYAVCVEIKLMLGSVTLPNTTVRTRFRRPREGLLFLQDITLPNIDLSTLPSGTVIVFAAYGVRLEQKEVLLSYVKQPVVEFNGCLVAGRKCLPMWIVPNDDKGALDDINFARATCRPNLSVVDAPTLNVRFDSYLCPVLCPVAQPPIPLDTQLGGPVSLNTMNAKIRNRLEGIVSRDALYEFDSEEKEILWMARYNYMNDASVLPKLMRCVDWLSPQQAAEAYHLLYAWAKPEHPLHALALLDVHYADYRVREYAVSILEELSDSDLQQVLLQLVQVLKFELNHDSPLARFLLRRGIKNPYQIGHFLFWHLKAEIHNPDVCERFALLLENYLVNIKRHARELLSQDYVIKQLQAVAEQVQFQKTKELMADVECKELLRENLGKLNRLFPERWQIPLNPKWQVRALKPQFCKYMSSKKVPLWLCFANSDPDGGDIRVIFKTGDDLRQDLLTLQLFRIMDRTWLRQGKDMALKPYTCVATGVTRENEGVGMIEVVGDSATISDIQIEEGGGALGAFKNKTLVKWLQKHNRGTPEFNAAVDNFMRSCAGYCVATFCLGIGDRHNDNIMLTKDGHLFHIDFGHFLGNFKSKYGFKRERFPFVFTPEMAQVMGGLKSHEYKEFVALCCSAFNILRKEASLFLNLFSLMIPAGMPELSCTEDIHYLRDQLLLTLSDDAASKAFKKEIKKTISDFYRRVDNSIHILVKH* |
| **>PlasB_01656 (PbRas)** | MDAAKDGKPGSPALPVKIVVVGDGAVGKTTLLLRYVENRFPETYVPTVFENYYRDVVVEGIAVNMGLWDTAGQEDFDRLRSLSYNDTDLVLIVFSIDAPTSLANVSSKWVPEIQHHCEGVPFLLVGTKSDLRNDEQTLEKLRARNQKVVELSDANAVGKEIGAQAILECSALTGAGIKEVFDQALKVVLVKKGLLKTEKPQSTSCCTLM* |
| **>PlasB_04173 (PbMEK)** | MAPPNTRANTRSRQQQPGLTSRRQSGRNNKPGFRRRRPPALCVDENDVDDVLKSQVRVAIRLWNEPRAFVIGDFQIDGKGFTLGPSVKAAGLTSLVDSSRPVSDADVVDGHQTTRVDIQISGINELEVFAVCGVGSTSRVRIVRHKATRQLLALKQVDLDTSAERVQPIIAELRALHECNSPSIVSFHGAFYSNSCASIVMEYMDSGSLKDLATRSLNSSIPEYIISAIAKQILHGLVYLHCSKGIIHRDLKPSNILLNSMGRVKLADFGVSGKVSNVTRNRHTFVGTVSYMSPERIMGENHTVSSDIWSFGITLMECALGYFPFTQTAFRSRSKPAFWDMLDTVSTCPTPRLPGNMFSNEFNDFVSSWYINNVSSVKHDILDVRNLDCVPVPVEAATVRTFTFA* |
| **>PlasB_00238 (PbERK1)** | MSDSTPAVDETVTGSTSRPTLSNAAEPSTGGASPPAATPNWNERHQFSASGTQFLVDKQYTPVKSIGSGAYGIVCSAMDTVTQKKIAIKKISAAFDDLIDAKRILREIKLLSHFKHDNVIRLVDMVNPLTAAQFDDVYMVMECMETDLHKIIYSKNELTDEHCQYFIYQILRAMKYVHSSNVIHRDLKPSNLLLNGNCDLKVCDFGLARGTNDKDDYELTEYVVTRWYRAPEIMCACQDYDRKIDVWSIGCIFGEILGRKPLFPGDNYIHQLNLIFGTLGTPSDADLDWITNAKALQYIKNLKKKPSMPFERVYPKAPPQAIDLLSKMLVFNPANRISVEDALAHPYLKALHNPKDEPICPTTFDFEFEKQANTKVGIQKLMFEEIEKFRPGVVNPIL* |
| **>PlasB_02211 (PbERK2)** | MRTGDDRRRPSQASEAEREAAMRSRPVSTVTPGGTRSFMVLGNRFNVRDRYKLIRVLGFGAFGVVCAADDTWTRTQVAIKKISNAFSNLIHTKRILREVRLMRYFCHDNIIKILDLMRPTGDVADADDIYIVTELMETDLHQIIVSPQKLTIEHVQYFLYQILRGLRCIHSAGVLHRDLKPSNLLLNSNCDLKIADFGLARMASAEDDTNAFMTEYVATRWYRAPEIMLSWKEYSKAIDMWSVGCIFAELLGRRPLFQGRDYMHQLHLITDVLGTPSFADTEYIASPKAKEYIRSLPIKPRIPLQSLYPGAPADALDLLQKMLMFSPEKRISVDEALTHPFLASLHDPTDEPTASGAFDFSFESVDLDVDTLRDLLWNEAEQYEKSDPAGHVDASITPMSY* |
| **>PlasB_05287 (PbSGK)** | MLRPHHDQALPTVTATPGQTFAIDRAGCRSVHRTADDFEAIRRVLQTRYDFREPILSAGSGAAVGMQAWLQKALTSPLTRSSPELRRFLEPDSADSEDDIDADLDDALGNLSVDEDLDKEIKQSLSVPTVPASLDDFQLLKVIGKGAFGKVMLVRSKSDPKQLFALKQLDKSKVRNKGQSEHAMTERNILEYIRHPFIVTMRGAFQTESKLFIVLDYCAGGELFFHLQRMGTFSEELARFYTAQLVLAIEYLHELGIVFRDLKLENVLLDAKGNIQLTDFGLSKEGVADNVSARSFCGTPEYLAPEVLTGTGYGRAADWWSLGTLLFEMLTGLPPFYAKDSKQLFQRILTSRLRLPYHVSPEAGDLLQRLLSRNVESRIGSSDDDAREIKQHCFFAGIDWDLLLAKEVPPPFNPCDGLAPDDTSNFDIDFTNLPVASTDLASGSFVKDVDPDLSNSLSDFPFPLNPASGQR* |
| **>PlasB_03214 (PbCDK)** | MAGDGERPESQQYAGLGRYQKIEKIGEGTYGVVYKARDRVTGESIALKKIRLDSEDEGIPSTAIREISLLKELQHPNVVSLRDVVHHDFKKLYLVFEYLDQDLKKYMDSQTAPLDLMLVKSYMQQLLKGIAFCHSHRTLHRDLKPQNLLIDRQGALKLADFGLARAFGVPVRPYTHEVVTLWYRAPEILLGTKEYSTPVDIWAAGCIFAELVTKQPLFPGDSEIDELFRIFRALGTPNETTWPRVTSMPDYKSTFPKWDPRPLSTCVPGLDRVGLDLLGKMLRYEPGKRISAKQALEHAYFDDLYDLQR* |
| **>PlasB_10022 (PbCyclin1)** | MTRARPSERLAPAVASTPSASSKASTAMPISPLATRPPSLNLIDEFSNMLVNEQKFHAEMSYVDDYRQFSYDMRSRLLGWIVEVLSYVDASEQTLFLAAAIFDRFFRLGGCHKALWALLAGTSIFIASKYYQISAVRVRLITEALHNTYTRQDVYDMEMAVLGRLDFNISLVTAWDFAVFFLSRLPSLPQDGETVIRSMTSYLLELALVDRMHFDMRPSSLAASCIALAVHHHLYTIGADAPLRDQVVTDLERAVSARHCFLVDGMRSLYRLHDGRSPADPVPKKYASDAYHNVSKAQLRCPTRY* |
| **>PlasB_07777 (PbCyclin2)** | MSRRAKRNRDAAADAAGDLSRRHRVQANAVAVGKVLLCRELDDGAGACMTRWIEPDVCRGALLDTTTTMIERMRTRQSTYDTNLNPNYLTGHPCLRPKMRSIVVDWMQQVCQEYGLSRETCHLATNYFDRYVCGQLATPPTRLQLVAVTALLIASKLEDVNVPTVADFAYTCAGSYAAAEILRTELDMVKALGWMLRPHSPSTWARLFLLRGIDQATPGSKPGRDLLRSDRFVRVMEVIDAARLYVKSLQYSPYVLAASVLFHVFDGYADLIGLATGMDRAQCAECMEWIALLDDCIPRVGLHSILDRGYRPPDMHVRQVYNPHALQVCLDDTRHN* |
| **>PlasB_00247 (PbPKA1)** | MSFLFKPKRRRNNSGTSEDADLNLSPETTKIREYLASRENLSLSTFTIGATLGTGTFGRVRLATIAIDGKPKYMALKMLKKTEIIRLKQVEHIKDEKRILSAICHPFIVNFFGSFQDEKRLYMIMEYVIGGELFSQLRRARRFTNDASRFYAVEIVLALEFCHERNIVYRDLKPENILIDKEGHVKITDFGFAKFVEDRTWTLCGTPEYLAPEIIQSKGHGKAVDWWALGVLIFEMCAGYPPFYDENPFGIYQKILDGTIDFPKHFDPNAKDLVRRLLTADRTKRYGCLKNGVDDIKEHRWFKGIDWDAALQRKLTPPFIPTAMSENDTTNFDKYPDSVERGHPPEISAADAALFEAF* |
| **>PlasB_07097 (PbPKA2)** | MEWVEERTRGTPPPLRNSHRQASWGSKSVIYGGRGASSVLDDVFTIDMGSDGTWKEMVINGGFIPPARENHALVVVGDRMIVFGGSEYGRLSNEVYELSLSTCTWKKIETSGKPPSARERHTMTIVDNVAYIFGGQSAECEFLNDLYALDFSTWTWSLLNPPKAPTPRNHHSAVAVDTRIYVFGGKSHNGYFNDVHYYDISTRQWHQPQLQGISPAGRWGHTSIAFDRRIVIFGGWNGTWCFNDLNLLDTVTKAWTRLTTTTSGPSARAYHSASVYGGHSMMMFGGRNGLRRMDDTFTLELKESLGKAGSVEAMQALKNLWKLSDFELRETLGTGSFGRVRFAKHIPSGKFYAIKILKKAEILRLKQVDHILSEKTILQAINHPFIVNMFASFQDKKYLYLAMEYVVGGEFFTHLRRAGRFNNDSSCFYAAQIVLIFQYLHSKDIVYRDLKPENLLLDAKGNIKMTDFGFAKKVEFRTWTLCGTPEYIAPEILLNKGHGKPVDWWALGILIYEMLAGAPPFVDDDPMGIYQKILAGRIEYPAHFNRHAKDLISRLLTPDITNRLGNLKNGVEDIKRHKWFANINWTKLYNRKMLTPFVPKVRGDDDTHNFDHFPDSAEPPADVVLENDPFENF* |
| **>PlasB_01916 (PbGβγ)** | MTTKEKIEKCKKEIEDLKATIRDLRGDSDSADQPLQRVAREQGVSPGTCSIKQRRILKGHFGKIYAMHWSDDSRHLVSASQDGKLIVWNAFSTNKVHAIPLRSSWVMTCAYSPQGTFVACGGLDNLCSVYKLSNKAEGQQRTHCELAQHEGYLSCCRFINEGEIITSSGDSTCILWDIEQKQPKNTFSDHAGDVMSVSIFGPEGKTFVSGSCDATAKLWDTRADGKSAVKTFPGHESDINSVMFFPDGNAFGTGSDDSSCRLFDIRAYRQLNKYSSDKILCGITSVAFSKTGKMLFAGYDDYNCYVWDTLLGSNPSQLSGHENRVSCLGVAADGKALCTGSWDTFLKIWA* |
| **>PlasB_02182 (PbCaM)** | MATPDKQLTDEQIAEFKEAFSLFDKDGDGTITTKELGTVMRSLGQNPTEAELQDMINEVDADGNGTIDFPEFLTMMARKMKDTDSEDEIREAFKVFDKDGNGFISAAELRHVMTNLGEKLTDEEVDEMIREADVDGDGQINYEEFVKMMMAS* |
| **>PlasB_06593 (PbRaf-1)** | MDEASRDLAPPSATMNASEQSSVNDIRKAGHLKKQGGARGGRKNWKRRWVVLKSEYICYFGEKENGTSEDGPCLGVVPLRGAKIVSVEAIADETQLASDTPQSSSAAQLPTTCYQCHKANSSRIHCKSCQNVFCEACAAENQMIGDKATRVCSFCRHRHADEDTIAMAATASNMEIGSVVEVVYREAKNTVFFKKEKPQYLFAIRTKERELWLAAEDAQEKAAWVTAISEVIVSYKKAFNNANSATAGNQAEKEQKQDGKATGSPSQQWEIDYNQITILEKIGDGAFGEVFKGRLWGTDVAVKTIKADQVTESVVDDLKKEVAILSQLRHPNVVLYIGACTKPPNVCIVTEWCDKGSLHDVLHDSSIPLDIQRIVSLSVGIAQGINYLHSLERRIIHRDLKSHNVLVDRNFNVKVADFGLSHVRESLTQQNGTSALNNDTNGRTRANAFTAAAPQQLGAPADFQSMKINSESMGGHYGVFGTPEWMAPEIMEGTAYNQKVDVYSFGIMMSEILTRKLPFRDQYKIKSYMDVVDAVLDDGAMPTLPGWIGLRLKRLIESCLSRNSSARPSFMSIILKLRSMFSASPAELFQTYDLPRLHDMLATPDRHNQALAAKEIASFKFEQEACPAGCDDDIVIREIDMAKRVCARCRYSAAHHQKLDDAVVTQFIGKLTDMLASSSGKVVLPAVEALERLITGGQRKSSTKLRQENLSIIKERNGIERLLGLVSSRSPQLSEAASSCLMLLIEDLPVDDSSYKQLKGTSLHVLSAMMEKDIAQRRDENAQMTEQLRNKVKTFETLQRLVQAAQIQQQPPSSSENVLEDRPMLSSSQRRRPKSPVQVPVNPAATLATAAVTDQWSDQGTVVAAVAAVPAPVITVQKPPDAPARPDPGPRFTSKGTTAVVRPPARETAPDDMPIVALDETPTNNLDTASSSAVPSASVPSTPGPSDPADDDGAGSPRNTAPEAVQQLVRPQTPDVPLPGRFQRISPANGDWVFVHDAQLGEWILKYVIVTPSMICVFRSYDDVEQTRPPEATVNASVPPGKRLKFKTGRKFRQPWCFHLCSQGQKWWFSLRSSQHTEIWSDVIVRPLLPDALREAFGATQQTLLRPGGNSPQLSPRLDEMSADDAVNIEGPNRTQSAQSIAALAPGVASPGTVDQATSAVDQPIPATEQPGSPAVAVEQPAPAVEQPAPVVEQPAPVVEQPAPVVEEPAPVVEQPVPVIEQPEPVAEVASPAVVEAPRTMRTSVPVVPAMEAALDARGTSLVSRGSAEHPVVEFEEADTSTTQLLDIPMLEAEAEPLVMQSPVSMAAHLESATSAFGSTLSLDALAKADQGPPESEAQGSSKAPADAGATKRLSTVAATDTGDKSPPVALPTSSDRPQVINQAPEPTPSGAPATRRDTSPARDVPTQELVSEQTTTSEAAGVPPSTEPRSVEVSPVPLSSEPVAALSSSPNRQLEPASAVARAHGVEPASRDSPVVSPKEVAAGKFVKRYIETTGARSPMLKPNQRLLSLRPGNELSRGNAALRLAAMPEVTSLPPTFVEQFGRRDCWHYSYLIMPDRLSQQWVAKFVVLLGAQPIRLMVYNSHNDPPQKPISEHLLEEASAATSQAVEACLGFPVEYDEKIALVVKEKPAQANTTEPSRPLLFLCASETDRHKWLSLIQRVVALSRV* |
| **>PlasB_09434 (PbRaf-2)** | MSLSLSATDDTAAPDRLSAEPIWEGYLMKQGGARGGFKNWKKRWFRLDAENLCYYKERPRAPTDAKSNDADTSSSIAEPAVLGVLPIRNATIVDLQGADDVAVKAVELPPAWDVGATACHDCKKEFSGSVRRRFCRACGLAHCSPCSPRSVLQSRIGFAEPVRVCNSCFNGITSAGGFKNADGGSRLDDGIIIVQTEQKKSKGFEWRSKKHLFILNSAELQMYLQAETSTVKLEVMTAIRSVVLYHQRKAQRATTGSAVTVAEPESSNNRPHKLDRQMSASSQWNIDFKAIKILHEIGEGSTGMVYKGRLWGTDVAVKTIRNATMADDLQKEVAILSQLRHPNVVLYIGACLEKPNVAVVTEWCDRGSLFDLLYDDSPGCCNLDFQSIITIAIGIAQGMNYLHSLNQKIIHRDLKSHNILIDSHFTPKVADFGLSKVKEPTRLGDNIDPQIVTESLAIGHQKALRAHAAAKKKAAEDSKEAQSEVPGGTAEWMAPEVMEGAPYNQSVDVYSYGIVLSELLSRRLPFRDKYTITGYADVVEAVLDDGATPTIPEWCGAKMQKLVADCLSRNAEARPTFNEIILRLRSFYPLDPADVFQKFDIPRLLSMLDSPDRTEQFRAVRELSDMAVDLTDLCHVCGHTPLKYAQMGEATVVKFVARLTTMLLSPHADIIQHAMLALRAMIDESEAALRPTLFGAIRESGGVQRIALHLKSDKEDMANAAALLLSRIIADPGTVDNPATLMGLSPEVMAELLAIAKQEVSTLKKKIEGNEGSLSRKVELSTWISNRLRQAEFGLSDAQTALASIATPRTIIANVASPSPEGGSFEGKDLPVQLRAEMGRGAQCWGWLGLLDPAGEQWIDVYAVLRNATICIFDSEESRPETIRYIIRIRLGNVSAKFRAGRKRGRHNCLQIALSQELLPVAVDPKTPPIRRVFINVGSHERFAFWVRHINEHLDIPVPLDFAPDETMT* |
| **>PlasB_01538 (PbEif4E)** | MPGPAKRRNLFTTDGSDDESDADPFQTLLSSLKVDQKYEGMLKRRKLEEDGADQSEDDDDDTSVEDGERVLMSTEEIVEFQRKHGDAFEVAEDGEEDDSEQGDDESSDDGVADSNDDGDDNVPSDESEDDEDVANNDNDDSIAADPKDEYSDLDDGKPQGEDPVLPYHTRMFSNESASALQAKAPFANVSGGPPQWRVTMRRLAEDENESVWQLSPSTGDIQKSWPGNVVRGVEPEPSPLQPVVSRYQDLVYTCRKPGDDRATSDLYLRHVVRHVLRTLRTISDNDATLGSDPTFEARDQGFTHPTVLIMCPFRSSAHRIVNRVLELLPSKFTRQVSNIKRFNEEFGPPEPDAEEKDRFNRKPSLYKDVFSGNFDDNFRLGIKLGKRSVKLFADFYASDIIIASPLGLRMIVGGEGDLQSERDYDFLSSIEVAVLDQADVFMMQNWEHLQAVLSCMNLIPNDSSHSDFSRLRSWNLDGLAAHHRQMLIFASHGFPELNALSSKHCLSLAGCVRIRRHEFPGVLEQVVLPIRQQFQRFHHGDTPTDASEARFKWFAEEILPQLKTNLSSHILLFVPSYFDYVRIRNLLRSQKVKVALCSEYTKKGSVARARSRFFHGEQKFLLCTERFHFFRRYCIRGAHTIFFYGLPQEPTFYLDFVNAIEPGSHQTTIALFSRFDALALERVVGSSRAARLLSAHKDLHMFC* |
| **>PlasB_01918 (PbMOB1)** | MKGIFGMGQKTFKPTKSHEKGSKRDEMHLKAQATLGSGDMAMAVKLPKGEDMNEWLAVNTVDFYKYISLLYGTIAEFCTAESCPAMSAGDAFQYLWADGVKIKKPIKCSAPEYVDHLMSWVESQLNDEHIFPLQIGAPFPKNFQTQIIPTLFKRLFRVYAHLYHEHFTKMQALGAEAHLNTCFKHFMFFVREFNLIDKKEQEPLKDLIENLVSQ* |
