# Supplementary material for: Proto-oncogenes in a eukaryotic unicellular organism play essential roles in plasmodial growth in host cells
Source: BMC Genomics. 2018 Dec 6;19:881. doi: 10.1186/s12864-018-5307-4 (PMC6282348; doi:10.1186/s12864-018-5307-4)
Supplement: Supplementary file 6 — Table S4. Alignments of P. brasicae proteins associated with cancer-related signaling pathways with their homologs. (DOCX 105 kb) [file 12864_2018_5307_MOESM6_ESM.docx]

**Additional file 6: Table S4** Alignments of *P. brasicae* proteins associated with cancer-related signaling pathways with their homologs

| **Gene** | **Homolos** | **Alignment with its homologs** |
| --- | --- | --- |
| PlasB_04559 (PbPI3K-1) PlasB_04589 (PbPI3K-2) | gi\|922853879: phosphatidylinositol-4,5-diphosphate 3-kinase, [*Chrysochromulina sp*. CCMP291]  gi\|551597914: phosphatidylinositol-3-kinase [*Emiliania huxleyi* CCMP1516]  gi\|470490961: phosphoinositide 3kinase [*Acanthamoeba castellanii str*. Neff]  gi\|470452549: phosphoinositide 3kinase [*Acanthamoeba castellanii str*. Neff]  gi\|281211934: phosphatidylinositol-4,5-diphosphate 3- kinase [*Polysphondylium pallidum* PN500]  gi\|66815877: phosphatidylinositol-4,5-diphosphate 3- kinase [*Dictyostelium discoideum* AX4]  gi\|531998556: phosphatidylinositol 4,5-bisphosphate 3- kinase [*Microtus ochrogaster*]  gi\|617436555: phosphatidylinositol 4,5-bisphosphate 3- kinase [*Poecilia formosa*]  gi\|754345946: phosphatidylinositol 3-kinase [*Capsaspora owczarzaki* ATCC 30864]  gi\|761903800: phosphatidylinositol 4,5-bisphosphate 3- kinase [*Amphimedon queenslandica*] | gi\|922853879\|gb 1 RLKVLIEQ----------------DPLADLTQDDKQILWTHRHFILSSPIALPKFLQSVSWADHRQVAEMHALLGRWA-PLKPVAALELLDAKFADARIREYAVGCLED-FSDTDLELYV gi\|551597914\|re 1 RLKRIIEQ----------------DPLSPLSLDEKELVWRHREFVSSSPEALSKLLESCKWDDRTAVAEAHALLRKWA-VPSPTQALKLLDAKFADPQVRAYAVERLGE-MSDTALAELV gi\|470490961\|re 1 QLEEIMKKGYELRLCSSATAALVGHPLTALGEDERQLLWECREFCAQFPSLLPRFLQYVKWNDRESVLEAYRMLALWS-PPTPFEALSLLDAKYASPVVRAYAVKRLHE-LDDRALSDFL gi\|470452549\|re 1 KKRLVDDAAVPESIPAKIDRILSQDPLYLLKPDEKMLLWDHRYHLLSKPKALPKFLLSITYQDRYQVQEMHHLLAKWPHFPNPVDALELLDAKFADAAVRQYAVECLDE-LSNEQLGDYL gi\|281211934\|gb 1 FFDSIIRV----------------DPLSDLSREKYQQLWSLRQYAIHVPQILPRLMLSVPWTNPAAVDEIHSLISRWPR-LTPYDALALLDAKNVDYYVRTYAVHCLDA-LSEEELLDIL gi\|66815877\|ref 1 FFEQITAL----------------DPLSDLKQEKYNQLWTLRHYSILFPQVLPRLMLSVPWTQATAVDEAISLLDRWPK-LKPYESLELLDAKHANRKVREFAVTCLED-LSEDELLDIL PlasB_04589 1 RLEGIVSR----------------DALYEFDSEEKEILWMARYNYMNDASVLPKLMRCVDWLSPQQAAEAYHLLYAWAKPEHPLHALALLDVHYADYRVREYAVSILEE-LSDSDLQQVL PlasB_04559 1 NLDILIKT----------------DPLYVLTPKDKEIFWTTRHHLLPYPLALPKFLQSVDWTSVDHRNEAHRLLSNWTPPLNPVSALELLDARYADYKVREYAVNCLRV-LPDDELQLFL gi\|531998556\|re 1 QLRALCTR----------------DPLSEITEQEKDFLWSHRHYCVTIPEILPKLLLSVKWNSRDEVAQMYCLVKDWP-PIKPEQAMELLDCNYPDPMVRSFAVRCLEKYLTDDKLSQYL gi\|625231901\|re 1 QLRALCTR----------------DPLSEITEQEKDFLWSHRHYCVTIPEILPKLLLSVKWNSRDEVAQMYCLVKDWP-PIKPEQAMELLDCNYPDPMVRSFAVRCLEKYLTDDKLSQYL gi\|617436555\|re 1 QLRSLCSR----------------DPLYELSEQEKDFLWRHRHYCLNIPESLPKLLLSVKWNSRDEVSQMYCLLKEWP-LMEPESALELLDCNFPDPIVREFALRCLVQGLTDDKLSQYL gi\|754345946\|re 1 RLQKIVTR----------------DPLARMTKKDKALVWKYRLYLRKDAKALPKFIRSVPWNKYELVAQAHALLEVWE-RPTAEDALELLDSEFADARVRAYGVACLDK-LSDDKVMTYL gi\|761903800\|re 1 ELEKSISS----------------DPLANLDKVEKELLWKFRYQLTNSPEALPKFLQCVDWASLEQVTETHKLLQLWS-PITMEVGLELLDYHFADEKVRTMAVARLSR-LTNDEVLVFL  gi\|922853879\|gb 103 LQLVQVLKYEARHDSALARFLLRRALSSPHQVGHQFFWCLKAGMHAPEVCERFGLLLQEYLRCCG-EHRSELELQCRVESMLVASAELVKTI------RKPERKRVLQAELA-------K gi\|551597914\|re 103 LQLTQVLKYESRHDSALARLLLRRALRCPHQVGHRFFWALKAEMHLPEVSERFGLLLQEYLRCCG-PHREKLLLQSRVEQSLIAIARAVQRE------KKSERIALLRQKLS-------E gi\|470490961\|re 119 LQLTQVLKYEAYHYSPLAEFLLSRALMNVNQIGHAFFWLLKSELHNPTIASRYSLLAEAYLRGCG-AHRQELVKQNKVLNQLAKVAMRVKEAP-----SSSEKKKVLEDGLR-------R gi\|470452549\|re 120 LQLVQVLKYELYHDSALSRFLLRRALRS-KELGHMLYWYLKSEVHVPTIAERYELLLEAYLRGCG-SHRAELKKQEDVHKEMARIAYRIKEIK-----EAEKKKETLQEELKRFGERLEK gi\|281211934\|gb 103 LQLIQVLKSEPFHDSPLSRFLLKRAILS-RTIGHHFFWYLKADLHVTNIAERFGLLLESYLQACG-SHREEMGRQLQVIDSLTAVARKIKPLK------DQDRREVMLKELEKIE----- gi\|66815877\|ref 103 LQLVQVLKYEPFHDSKLSRFLLRKAILN-RNIGHSFFWYLKSDLHDSNLSERFGILLESYLYACG-AHRIELLKQMEVINNLTEVAKKIKPLK------DQDRREFMIKEFESLE----- PlasB_04589 104 LQLVQVLKFELNHDSPLARFLLRRGIKNPYQIGHFLFWHLKAEIHNPDVCERFALLLENYLVNIK-RHARELLSQDYVIKQLQAVAEQVQFQKTKELMADVECKELLRENLGKLNR---- PlasB_04559 104 LQLAQSIKNEPYHDSPLTRFLVERALRNPHSIGHSLFWHLKAELHSPFHCERYAMILEEYLFHSG-WHAAELRKQYEIVQRLQRISGKVVARKHAKDMTDDEIQAEYLAALHKFNEN--Y gi\|531998556\|re 104 IQLVQVLKYEQYLDNLLVRFLLKKALTN-QRIGHFFFWHLKSEMHNKTVSQRFGLLLESYCRACG-MYLKHLNRQVEAMEKLINLTDILKQE------KKDETQKVQMKFLVEQMRQPDF gi\|625231901\|re 104 IQLVQVLKYEQYLDNLLVRFLLKKALTN-QRIGHFFFWHLKSEMHNKTVSQRFGLLLESYCRACG-MYLKHLNRQVEAMEKLINLTDILKQE------KKDETQKVQMKFLVEQMRQPDF gi\|617436555\|re 104 LQLVQVLKYEMYLDNPLARFLIKKALTN-QRIGHFFFWHLKSEMHNKTVSRRFGLLLEAFCRACG-MYLKHLNRQVEAMDKLVNLTDTLKQE------KKDETQKTQMKFLVEHMSRPDY gi\|754345946\|re 103 SQLVQVLKYEPYFDCELARYLLRRALRN-QRIGHFFFWYLRAEMHLPDVAARFALLIEAYCRGCG-SHMSMLARQVNAMDKLEGIANLIKR-------KDLDTREKKLTLLRAEITS--- gi\|761903800\|re 103 LQLVQVLKFEPYHDSALARFLLKRALLS-KRIGHFFFWYLRSEMDSPQFSQRFGILLEAFLKGCGKNIFIELQKQHQAVQDIMSIASGLKKH------MEKHKG-SATDYAKADLSQ---  gi\|922853879\|gb 209 VAFPLKFTLPLSPRFECSGLRIEKCKTMDSKKVPLWLVFSNADPIGADLY----VIFKCGDDLRQDQLTLQILRIMERRWERAGLDLQLSAYRCVATG-----NELGFIEVVLNSTTTAD gi\|551597914\|re 209 TELPPRFQLPLDPRIECTGLVVSKCRCMDSKKVPLWLVFSNADPQGTDVT----VMFKCGDDLRQDALTMQLIRSMERKWEAEDLDLRLSPYGVVATG-----DEVGFIEIVLNSNTTAN gi\|470490961\|re 226 MEFPARFQLPLDPRQEVKGVIVDKCKYMDSKKQPLWLVFENAEKRGAPIR----VIFKAGDDLRQDVLTLQMLRIMDKLWKEEGLDLRLNPYGCVATG-----DELGMIEVVLNASTTAS gi\|470452549\|re 233 TPALTPFQLPLDYKRQAKGVLIHKCKYMDSKKKPLWLVWENADATQPPIY----TIFKAGDDLRQDMLTLQMLRIMDKLWKSEGLDLKLSPYGCVSTG-----HELGMIEVVTQSETTAN gi\|281211934\|gb 210 --WPKRFQITLNPKFESNGLINQRCKYMDSKKLPLRLSFTNIDMGADTTE----VIFKVGDDLRQDMLTLQMIRLMDKLWQKEGLDFKLSPYGCIATG-----DMIGMIEVVLNSETTAK gi\|66815877\|ref 210 --WPKRFHLTLNPRFESNGLIINKSKYMDSKKLPLRLSFTNTDMNADPIE----VIFKAGDDLRQDMLTLQMIRLMDKLWQKEGLDLKLSPYGCISTG-----DMIGMIEVVLNSETTAK PlasB_04589 219 -LFPERWQIPLNPKWQVRALKPQFCKYMSSKKVPLWLCFANSDPDGGDIR----VIFKTGDDLRQDLLTLQLFRIMDRTWLRQGKDMALKPYTCVATGVTRENEGVGMIEVVGDSATISD PlasB_04559 221 LSTNESTQIPLNPKWLVTTLVVEKCRFMSSKMAPLWLVFKNADPLGDNII----IMFKSGDDLRQDILTLQLLKVMDKIWVSEDLDMRLIPYNCVSTGINAQGKGVGLIEIVLNSDTTSG gi\|531998556\|re 216 MDALQGFLSPLNPAHQLGNLRLEECRIMSSAKRPLWLNWENPDIMSELLFQNNEIIFKNGDDLRQDMLTLQIIRIMENIWQNQGLDLRMLPYGCLSIG-----DCVGLIEVVRNSHTIMQ gi\|625231901\|re 216 MDALQGFLSPLNPAHQLGNLRLEECRIMSSAKRPLWLNWENPDIMSELLFQNNEIIFKNGDDLRQDMLTLQIIRIMENIWQNQGLDLRMLPYGCLSIG-----DCVGLIEVVRNSHTIMQ gi\|617436555\|re 216 MESLQGFVSPLNPVHQLGNLRLEECRIMSSAKRPLWLNWENPDIMSELLFTNNEIIFKNGDDLRQDMLTLQIIKIMENIWQNQGLDLRMLPYGCLSIG-----DRVGLIEVVKNSYTIMQ gi\|754345946\|re 211 -TTLPTFQTPLNPTVVVRKLIPEKCSFMDSKKLPLWLVFENDDPDGEDVY----IIFKVGDDLRQDMLTLQMIALMDNLWQSEGLDLRMIPYGCLSTG-----DNTGMIEVVMNSNTVCN gi\|761903800\|re 212 QNLPEYFAPPFDPSLRMGNLQVEKCRVMDSKKTPLWLEFMNMDP-SSTTQKPIKVIAKHGDDLRQDMLTLQMLTIMDSLWQSEGLDLYIIPYGCVATG-----NEMGMIEVVQDAETVAK  gi\|922853879\|gb 320 ITKTYSGGASGAFSKEPMAMYLREHNPTPDAYRRAVDTFLVSLAAYCVATYVMGIGDRHNDNIMLARTGHLFHIDFGHFLGNFKSKFGIRRERAPFVFTPDFAYVLGEK-----GADDYA gi\|551597914\|re 320 ITKEYGGGASGAFAKEPMAHYLREHNKSEREYAAAVETFAHSLAGYCVATYVLGIGDRHNDNVMLSKNGHLFHIDFGHFLGNYKSKFGIKRERAPFVFTPDFARVLGDK-----GHSDYE gi\|470490961\|re 337 VNKEK-GGSKAVLYKDTLTSWLKEKNPSAEAFEAAQENFMLSCAGYCVATYVLGIGDRHNDNIMITQFGHLFHIDFGHFLGNVKSKLGIKRERAPFIFNPQFAHVLGGS-----QGTRYA gi\|470452549\|re 344 ISSAA-AGVKGAFAVDPIANWLAAKNKNGK-YRRAVENFISSCAGYCVATYVLGIGDRHNDNIMLNESGLLFHIDFGHFLGNYKKKLGIKREKAPFVLTPDFAYVMGGR-----DSKDFK gi\|281211934\|gb 319 IQKSAGG-ATAAFKLDPLANWLLQHNKTEQEYQKAVDTFILSCAGYCVATYVLGIGDRHNDNLMCTKLGRLFHIDFGHFLGNYKKKFGFKRERAPFVFTPDFCYVMGGK-----DSAKFL gi\|66815877\|ref 319 IQKSEGGGAASAFKLDPLANWILQHNKSDMEYQKAVDTFILSCAGYCVATYVLGIGDRHNDNLMVTKGGRLFHIDFGHFLGNYKKKFGFKRERAPFVFTPDFCYVMGGK-----ESFKFS PlasB_04589 334 IQIEEGGGALGAFKNKTLVKWLQKHNRGTPEFNAAVDNFMRSCAGYCVATFCLGIGDRHNDNIMLTKDGHLFHIDFGHFLGNFKSKYGFKRERFPFVFTPEMAQVMGGL-----KSHEYK PlasB_04559 337 IQLKYGGGALGALKLDPLHKFIADHNKEPDQYESAKDNFIKSCAGYCVATYVLGIGDRHNGNIMLTKDGRLFHIDFGHFLGNFKSKFGIKRERAAFVLTPEMAFVMGGKN--YKKAKEFR gi\|531998556\|re 331 IQCKGGLKGALQFNSHTLHQWLKDKNKGE-IYDAAIDLFTRSCAGYCVATFILGIGDRHNSNIMVKDDGQLFHIDFGHFLDHKKKKFGYKRERVPFVLTQDFLIVISKGAQEYTKTREFE gi\|625231901\|re 331 IQCKGGLKGALQFNSHTLHQWLKDKNKGE-IYDAAIDLFTRSCAGYCVATFILGIGDRHNSNIMVKDDGQLFHIDFGHFLDHKKKKFGYKRERVPFVLTQDFLIVISKGAQEYTKTREFE gi\|617436555\|re 331 IQCKGGLKGALQFNSNTLHHWIKDKNHGE-AYDRAIDLFTRSCAGYCVATFILGIGDRHNSNIMVKENGQLFHIDFGHFLDHKKKKFGYKRERVPFVLTQDFLIVISKGIQESTKTKEFE gi\|754345946\|re 321 IQKDKGGGAHGAFKDDPLYKWLVEKNPDEKATERAVEMFMLSCAGYCVATYVLGIGDRHNDNIMMNKSGQLFHIDFGHFLGNFKSKFGVKRERVPFVLTPDFVYVMSQ--KEGTKGERFL gi\|761903800\|re 326 IQLHHG-GSFSTLKDEPLYEWLKKKNPNPKHFEQAIERFVYSCAGYCVATYVLGIGDRHNDNIMLTVSGNLFHIDFGHFLGNTKAFLGVNRDRAPFVLTPDFEYVLGK-----RTSENFK  gi\|922853879\|gb 435 RFSDLCGRAYNVIRAHANEFINLFQLMLSTGIPELQRAEDINWLRECMLIGHSEESASAHFANRITVALH-TRTTQLNNAVHILAH- gi\|551597914\|re 435 HFVALCCRAYGILRGHSHEFITLFQLMLSTGIPELQRAEDIFW-------------AAEHFTKLISAALS-SRTTQVNNAVHIIAH- gi\|470490961\|re 451 QYVETCQRAYNIVRKHAHMFINLFMMMLSTGIPELQCEEDILYLCGSLLVGTEEKAAAENFAKLIEEARK-TKSVLVNDIVH----- gi\|470452549\|re 457 TFCDKCCKGYNILRKHSNMFINLFCMMLSTGIPELTSPNDIQYLRTALCLSLTDEEAAEEFQKLIYESLD-TKTTQWNNLAHMIVHR gi\|281211934\|gb 433 QFVNYCCNAYNILRRHARLFMNLFAMMVSTGIPELQSMEDLNYLRESFSLELTDEKAREKFTALIYESLT-TKTTQLNNAIHILAH- gi\|66815877\|ref 434 QFVNYCCTAYNIVRKNAKLFMNLFAMMVSTGIPELQSMEDLNYLKESFSIELSDEKAREKFVALIHESLA-TKTTQLNNFFHHLAH- PlasB_04589 449 EFVALCCSAFNILRKEASLFLNLFSLMIPAGMPELSCTEDIHYLRDQLLLTLSDDAASKAFKKEIKKTIS-DFYRRVDNSIHILVKH PlasB_04559 455 VFTDLSAQAFTILRNHASLFINLFSLMVSAQMPELMFQEDIHYLRDKFFLQDSEKGAVERLRKEIQKSLN-TTYRQFDNLIHNIKHK gi\|531998556\|re 450 RFQEMCYKAYLAIRQHANLFINLFSMMLGSGMPELQSFDDIAYIRKTLALDKTEQEALEYFTKQMNDAHHGGWTTKMDWIFHTIKQ- gi\|625231901\|re 450 RFQEMCYKAYLAIRQHANLFINLFSMMLGSGMPELQSFDDIAYIRKTLALDKTEQEALEYFTKQMNDAHHGGWTTKMDWIFHTIKQ- gi\|617436555\|re 450 RFQEMCYKAYLAIRQHASLFINLFSLMLGCGMPELQSFDDIAYLRKTLALEKSQQEALEYFTKQMNDAHHGGWSTKMDWIFHTIRH- gi\|754345946\|re 439 RFTDLCEKSYLILRRHANLFINLFAMMMSTGIPQLSCSDDINYLRDALRLGSSDEAAAIEFRKKIDEALAKCWSTRLNWTIHGMVH- gi\|761903800\|re 440 KFEEIAVRAYLIIRKNANLFINLFSMMKCTGIPELRSVEDLDYLKSVLVLGKNEEQAADHFRQQIQKCLRLQWSTQLSWLAHNWVHR |
| PlasB_01656 (PbRas) | gi\|209152974: Ras-related C3 botulinum toxin substrate 1 precursor [*Salmo salar*]  gi\|556985398: Ras-related C3 botulinum toxin substrate 1 [*Latimeria chalumnae*]  gi\|830017821: ras-related C3 botulinum toxin substrate 1 [*Microcebus murinus*]  gi\|675384139: Ras-related C3 botulinum toxin substrate 1 [*Stegodyphus mimosarum*]  gi\|303313107: Rho GTPase, putative [*Coccidioides posadasii* C735 delta SOWgp]  gi\|906958668: Ras-like C3 botulinum toxin substrate 1 [*Sphaeroforma arctica* JP610]  gi\|551569378: Rac1, RHO family GTPase [*Emiliania huxleyi* CCMP1516]  gi\|340382883: Ras-related C3 botulinum toxin substrate 1-like [*Amphimedon queenslandica*]  gi\|927413356: Ras small GTPase Rho1 [*Trichoderma virens* Gv29-8] | gi\|209152974\|gb 1 -VVVGDGAVGKTCLLISYTTNAFPGEYIPTVFDNYSANVMVDGKPVNLGLWDTAGQEDYDRLRPLSYPQTDVFLICFSLVSPASFENVRAKWYPEVRHHCPNTPIILVGTKLDLRDDKDT gi\|556985398\|re 1 -VVVGDGAVGKTCLLISYTTNAFPGEYIPTVFDNYSANVMVDGKPVNLGLWDTAGQEDYDRLRPLSYPQTDVFLICFSLVSPASFENVRAKWYPEVRHHCPNTPIILVGTKLDLRDDKET gi\|830017821\|re 1 -VVVGDGAVGKTCLLISYTTNAFPGEYIPTVFDNYSANVMVDGKPVNLGLWDTAGQEDYDRLRPLSYPQTDVFLICFSLVSPASFENVRAKWYPEVRHHCPNTPIILVGTKLDLRDDKST gi\|675384139\|gb 1 -VVVGDGAVGKTCLLISYTTNAFPGEYIPTVFDNYSANVMVDGKPINLGLWDTAGQEDYDRLRPLSYPQTDVFLICFSLVNPASFENVRAKWYPEVSHHCPNTPIILVGTKLDLREDKET gi\|303313107\|re 1 -VVTGDGAVGKTCLLISYTTNAFPGEYIPTVFDNYSASVMVDGKPISLGLWDTAGQEDYDRLRPLSYPQTDVFLICFSIVSPPSFDNVKAKWYPEIEHHAPGVPIILVGTKLDLRDDKAT gi\|906958668\|gb 1 -VVVGDGAVGKTCLLISYTTNAFPGEYIPTVFDNYSANVMVDGKPINLGLWDTAGQEDYDRLRPLSYPQTDVFLTCFSLISPSSFENIKTKWLPEISHHCPNVPFLLVGTKLDLREDQEV gi\|551569378\|re 1 -VIVGDGAVGKTCLLISYTTNAFPEDYIPTVFDNYSANVMVDGKPINLGLWDTAGQEDYDRLRPLSYPQTDVFLVAFSLISRPSYENVKQKWFPELKHHCPNCPFILVGTKLDLRSDGTT gi\|340382883\|re 1 -VVVGDGSVGKTCMLISYTTNSFPGEYVPTIFDNYTANVFVDGRPISLGLWDTAGQDDYDRLRPLSYPDTDVFLICFSLVNPNSFANVADKWWPEIGHHAPGVPKILVGTKLDLRDNMGE gi\|927413356\|re 1 -VIVGDGACGKTCLLIVFSKGAFPEVYVPTVFENYVADVQVDNKHVELALWDTAGQEDYDRLRPLSYPDSHVILICFAIDSPDSLDNVLEKWISEVVHFCSGLPIILVGCKADLRDDPNT PlasB_01656 1 IVVVGDGAVGKTTLLLRYVENRFPETYVPTVFENYYRDVVVEGIAVNMGLWDTAGQEDFDRLRSLSYNDTDLVLIVFSIDAPTSLANVSSKWVPEIQHHCEGVPFLLVGTKSDLRNDEQT  gi\|209152974\|gb 120 IEKLREKKLTPITYPQGLAMAKEIGAVKYLECSALTQRGLKTVFDEAIRAVLC gi\|556985398\|re 120 IEKLKEKKLTPITYPQGLAMAKEIGAVKYLECSALTQRGLKTVFDEAIRAVLC gi\|830017821\|re 120 IEKLKEKKLTPITXSAGLAMAKEIGAVKYLECSALTQRGLKTVFDEAIRAVL- gi\|675384139\|gb 120 IEKLKERKLAPITYPQGLAMAKEIGAVKYLECSALTQKGLKNVFDEAIRAVLC gi\|303313107\|re 120 NENLRAKKMEPVSYEQALAVAKEIKAQKYLECSALTQRNLKSVFDEAIRAVL- gi\|906958668\|gb 120 LAKLREKNLSPITAAEGQALAKDIKAARYLECSALTQKGLKNVFDEAIRVVV- gi\|551569378\|re 120 VAKLRQQNQTPISLEEGQEMAKTIGATKYLECSALTQKGLKNVFDEAIRVVLC gi\|340382883\|re 120 LERLRSRNQKPIAITQGEAMRKKIGAISYKECSALTQAGLKDIFDEAIKVVL- gi\|927413356\|re 120 IEALRATNQKPVSSSDAEAVAKKIGAYKYLECSARTGSGVREVFEHATRAALM PlasB_01656 121 LEKLRARNQKVVELSDANAVGKEIGAQAILECSALTGAGIKEVFDQALKVVLV |
| PlasB_04173 (PbMEK) | XP_008387371.1: PREDICTED: mitogen-activated protein kinase kinase 6 [*Malus domestica*]  AFS18270.1:MKK6.2 [*Brachypodium distachyon*]  XP_007202262.1: mitogen-activated protein kinase kinase 6 isoform X2 [*Prunus persica*]  XP_008242472.1: PREDICTED: mitogen-activated protein kinase kinase 6 isoform X2 [*Prunus mume*]  JAT63362.1: Mitogen-activated protein kinase kinase 2 [*Anthurium amnicola*]  XP_020425046.1: mitogen-activated protein kinase kinase 6 isoform X1 [*Prunus persica*]  ONM38570.1: MEK homolog1 [*Zea mays*] | XP_007202262.1 1 -RLISEE---KEAQTSDSKELNFEIS-LEDLETIKVIGKGSGGVVQLVRHKWVGNLFALKVIQMN-IQEEIRKQIVQELKIN----QAAQCPHVVVCHHSFYHNGAISLVLEYMDRGSLA XP_008242472.1 1 -RLISEE---KEAQTSDSKELNFEIS-LEDLETIKVIGKGSGGVVQLVRHKWVGNLFALKVIQMN-IQEEIRKQIVQELKIN----QAAQCPHVVVCHHSFYHNGAISLVLEYMDRGSLA XP_020425046.1 1 -----------------SKELNFEIS-LEDLETIKVIGKGSGGVVQLVRHKWVGNLFALKVIQMN-IQEEIRKQIVQELKIN----QAAQCPHVVVCHHSFYHNGAISLVLEYMDRGSLA XP_008387371.1 1 ---------------------------LEDLETIKVIGKGSGGVVQLVRHKWVGKLFALKVIQMN-IQEEIRKQIVQELKIN----QAAQCPHVVVCHHSFYHNGAISLVLEYMDRGSLA XP_009595895.1 1 -RLISEE---NESPASETKEIDLQFS-LEDLETIKVIGKGSGGVVQLVRHKWVGTLFALKVIQMT-IQEDIRKQIVQELKIN----QASQCSHVVVCYHSFYHNGAISLVLEYMDRGSLA AFS18270.1 1 -RLISEEENGDEHQSTNMKVEDVQLS-MDDLEMIQVIGKGSGGVVQLVQHKWVGTFYALKGIQMN-IQEAVRKQIVQELKIN----QATQSPHIVSCHQSFYHNGVIYLVLEYMDRGSLA ONM38570.1 1 ----------DEDESTKLKVEDVQLS-MDDLEMIQVIGKGSGGVVQLVRHKWVGTLFALKGIQMN-IQESVRKQIVQELKIN----QATQSPHIVMCHQSFYHNGVIYLVLEYMDRGSLA JAT63362.1 1 -------------------------A-LDDMDMIQVIGKGTSGIVQLVRHKWTEQFFALKAIQLSNILEETRKQIAQELKINYSINHSSQCPYIVAYYQSFYDNGVITIVLEYMDGGSLA GAQ84491.1 1 ---------------------------LADLEVIKVVGVGSSGKVEKSRHRRTNKIFALKSVQLN-MKEEVRKQIVNELKIL----HRSSCPSIVKCYGAFHSDGVIKIVLEYMDGGSLS PlasB_04173 1 VDSSRPVSDADVVDGHQTTRVDIQISGINELEVFAVCGVGSTSRVRIVRHKATRQLLALKQVDLD-TSAERVQPIIAELRAL----HECNSPSIVSFHGAFYSNSCASIVMEYMDSGSLK  XP_007202262.1 111 DVIRQVK--TILEPYLAVVCKQVLQGLVYLHNERHVIHRDIKPSNLLVNHEGEVKITDFG VSASLASSMGQRDTFVGTYNYMSPERISGSTYDYSSDIWSLGLVVLECAIGRFPYMQSED XP_008242472.1 111 DVIRQVK--TILEPYLAVVCKQVLQGLVYLHNERHVIHRDIKPSNLLVNHEGEVKITDFG VSASLASSMGQRDTFVGTYNYMSPERISGSTYDYSSDIWSLGLVVLECAIGRFPYMQSED XP_020425046.1 98 DVIRQVK--TILEPYLAVVCKQVLQGLVYLHNERHVIHRDIKPSNLLVNHEGEVKITDFG VSASLASSMGQRDTFVGTYNYMSPERISGSTYDYSSDIWSLGLVVLECAIGRFPYMQSED XP_008387371.1 89 DVIRQVN--TILEPYLAVVCKQVLQGLVYLHNERHVIHRDIKPSNLLVNHQGQVKITDFG VSASLASSMGQRDTFVGTYNYMSPERISGSTYDYSSDIWSLGLVVLECAIGRFPYMQSED XP_009595895.1 111 DVIRQLK--TILEPYLAVVCKQVLQGLVYLHNERHVIHRDIKPSNLLVNHKGEVKITDFG VSAMLASSMGQRDTFVGTYNYMAPERISGSTYDYKSDIWSLGMVILECAIGRFPYIQSED AFS18270.1 114 DIIKQVK--TILEPYLAVLCKQVLEGLLYLHHERHVIHRDIKPSNLLVNHKGEVKITDFGVSAVLASSIGQRDTFVGTYNYMAPERISGSSYDYKSDVWSLGLVILECAIGRFPYTPSEG ONM38570.1 105 DIVKQVK--TILEPYLAVLCKQVLEGLLYLHHQRHVIHRDIKPSNLLVNRKGEVKITDFGVSAVLASSIGQRDTFVGTYNYMAPERISGSTYDYKSDIWSLGLVILECAIGRFPYIPSEG JAT63362.1 95 DFLKKVK--SIPEPYLAAICKQVLKGLIYLHHEKRIVHRDLKPANLLINHRGEVKITDFGVSATLESSSGQRETFVGTCSYMSPERIMGGRHGPLSDIWSLGLVLLECATGQFPYAHPDG GAQ84491.1 89 DVLRAVN--KIPEPFLGAISKQILLGLQYLHKLR-IKHRDIKPANLLLNRRGDVKIADFGVSSQAGSSMSKCGSWVGTVTYMSPERIEGGQYGVDSDIWSMGLSIVECALGSFPYS---- PlasB_04173 116 DLATRSLNSSIPEYIISAIAKQILHGLVYLHCSKGIIHRDLKPSNILLNSMGRVKLADFG VSGKVSNVTRNRHTFVGTVSYMSPERIMGENHTVSSDIWSFGITLMECALGYFPFTQTAF  XP_007202262.1 229 QQ-SWPSFYELLEAIVESPPPSAPSDQFSPEFCSFVS XP_008242472.1 229 QQ-SWPSFYELLEAIVESPPPSAPSDQFSPEFCSFVS XP_020425046.1 216 QQ-SWPSFYELLEAIVESPPPSAPSDQFSPEFCSFVS XP_008387371.1 207 QQ-SWPSFYELLEAIVESPPPSAPPDQFSPEFCSFVS XP_009595895.1 229 QQ-AWPSFYELLEAIVSSPPPSAPADQFSPEFCSFVS AFS18270.1 232 E--GWLSFYELLEAIVDQPPPGAPADQFSPEFCSFIS ONM38570.1 223 E--GWLSFYELLEAIVDQPPPSAPADQFSPEFCSFIS JAT63362.1 213 QE-AYNSVFELLEAIVLRPPPCAPSDMFSEEFCSFIS GAQ84491.1 202 QA-PNLGFWDLLTFIVHNPAPVLPPDQFSPEFCDFVA PlasB_04173 236 RSRSKPAFWDMLDTVSTCPTPRLPGNMFSNEFNDFVS |
| PlasB_00238 (PbERK1)  PlasB_02211 (PbERK2) | gi\|673024676: CMGC/MAPK protein kinase [*Aphanomyces invadans*]  gi\|813141432: CMGC/MAPK protein kinase [*Saprolegnia parasitica* CBS 223.65]  gi\|325188928: MAPK [*Albugo laibachii* Nc14]  gi\|301103436: MAPK [*Phytophthora infestans* T30-4]  gi\|754344951: ERK [*Capsaspora owczarzaki* ATCC 30864]  gi\|545707556: cyclin-dependent serine/threonine protein kinase [*Galdieria sulphuraria*]  gi\|599089093: MAPK4_2 protein [*Solanum tuberosum*]  gi\|315258227: Mitogen-activated protein kinase 4 [*Nicotiana attenuata*]  gi\|719992197: Mitogen-activated protein kinase 3-like [*Nelumbo nucifera*]  gi\|308808159: Mitogen-activated protein kinase [*Ostreococcus tauri*]  gi\|159484494: Mitogen-activated protein kinase 8 [*Chlamydomonas reinhardtii*]  gi\|545374258: Mitogen-activated protein kinase 6 [*Coccomyxa subellipsoidea* C-169] | gi\|673024676\|re 1 ---THSFVVSGTTFQVDTKYKF-IKPIGHGAYGVVISAQNTETGDKVAIKKVSKAFEDLIDAKRILREIKLLQHFDHENIITIVDLLPPT-SLAHFEDVYIISDLMETDLHRIIYSRQPL gi\|813141432\|re 1 ----HSFVVSGTTFQVDTKYKF-IKPVGHGAYGVVISAQNTETDEKVAIKKVSKAFEDLIDAKRILREIKLLQHFDHENIITIVDLLPPP-SLAHFEDVYIISDLMETDLHRIIYSRQPL gi\|325188928\|em 1 ------IAVSGTTFEIDQKYRF-LKPIGHGAYGIVISAENCETEEKVAIKKVSKAFEDLVDAKRILREIKLLQHFDHENVITVVDLLPPI-SLDFFEDVYIISDLMETDLHRIIYSRQPL gi\|301103436\|re 1 ---TYSFVVAGTNFQIDEKYKF-VKVIGRGAYGVVISAENTETNEKVAVKKISRAFEDLVDAKRILREIKLLQHFDHENVITIVDLLPPP-CLTQFDDVYIIADLMETDLHRIIYSRQPL PlasB_00238 1 WNERHQFSASGTQFLVDKQYTP-VKSIGSGAYGIVCSAMDTVTQKKIAIKKISAAFDDLIDAKRILREIKLLSHFKHDNVIRLVDMVNPL-TAAQFDDVYMVMECMETDLHKIIYSKNEL PlasB_02211 1 PGGTRSFMVLGNRFNVRDRYKL-IRVLGFGAFGVVCAADDTWTRTQVAIKKISNAFSNLIHTKRILREVRLMRYFCHDNIIKILDLMRPTGDVADADDIYIVTELMETDLHQIIVSPQKL gi\|754344951\|re 1 ----------GTRFDCPAKYSL-IKPIG-GAYGVVCSAQNNLLGRRVAIKKIIKAFEHLTDSKRTLREIKLLRHFHHENVLSIEDMLQPT-SYEQFDDVYLVSELLDTDLHQIIGSPQQL gi\|545707556\|re 1 FDTVRSYLVCGSRFDCPERFKL-LRPIGHGAYGIVCSAKDQVTGELAAIKKITKCFDHTTDARRILREVKLLRHFKHENIIGLKQILRPS-SFEAFEDVYLVTELMETDLHQIIVSKQSL gi\|599089093\|gb 1 GGRYMQYNVYGNLFEVSKKYVP-LRPVGRGAYGIVCAALNSETREEIAIKKIGNAFDNRIDAKRTLREIKLLRHMGHDNVIAIKDIIRPP-QTENFNDVYIIYELMDTDLHQIIRSNQQL gi\|315258227\|gb 1 GGRYVQYNVYGNLFEVSKKYVP-LRPVGRGAYGIVCAAMNSETREEVAIKKIGNAFDNRIDAKRTLREIKLLRHMDHDNVIAIKDIIRPP-QTENFNDVYIVYELMDTDLHQIIRSNQQL gi\|719992197\|re 1 DGRFIQYNIFGNLFEITSKYRPPIMPIGRGAYGIVCSVMNSETNEMVAIKKIANAFDNHMDAKRTLREIKLLRHLDHENVIAIKDVIPPP-IPRAFCDVYIATELMDTDLHQIIRSNQEL gi\|308808159\|re 1 -----KFAVCGTVFEVDAKYAP-IKPVGKGAYGVVCSAREVETNRKVAIKKIVNVFENVVDAKRTLREIKLLRHLRHENVIDIIDCVRPE-AMDAFEDVYLMYDLMDTDLYQIIRSSQSL gi\|159484494\|re 1 NREYNQFLVCGSLFECPAKYLP-IKPIGKGAYGVVCSAKNLDNQEKVAIKKIANAFDNVIDAKRTLREIKLLRHLQHENIVQIKDIIPPT-NRDAFKDLYVVYELMDTDLHQIIRSPQAL gi\|545374258\|re 1 ---KQAYSIWGTLFELDERYVP-IKAIGKGAYGVVCSAKNVVTGEKVAIKKIQNAFENLTDARRTLREIKLLRHLKHENVIAVRDILQPV-DKERFNDVYIVYELMDTDLHHIIRSPQQL  gi\|673024676\|re 116 SDDHIQYFLYQILRALKYIHSSNVLHRDLKPSNLLLNSNCDLKVCDFGLARGVEPDEDNME--LTEYVVTRWYRAPEIMLSTKEYTKAIDIWSTGCIFAELLGRKPMFPGDDYIHQLQII gi\|813141432\|re 115 SDDHVQYFLYQILRALKYIHSSNVLHRDLKPSNLLLNSNCDLKVCDFGLARGVEPDEDNME--LTEYVVTRWYRAPEIMLSTKEYTKAIDIWSTGCIFAELLGRKPMFPGDDYIHQLQII gi\|325188928\|em 113 SDDHVQYFLYQILRALKYIHSANVLHRDLKPSNLLLNSNCDLKICDFGLARGVEPEADNME--LTEYVVTRWYRAPEIMLSTKEYTKAIDIWSTGCIFAELMGRKPLFPGDDYIHQLQII gi\|301103436\|re 116 TDDHVQYFLYQILRALKYIHSANVLHRDLKPSNLLLNSNCDLKVCDFGLSRGVTPEEDNME--LTEYVVTRWYRAPEIMLSSREYTKAIDIWSTGCIFAELLGRTPLFPGDDYIHQLQII PlasB_00238 119 TDEHCQYFIYQILRAMKYVHSSNVIHRDLKPSNLLLNGNCDLKVCDFGLARGTN-DKDDYE--LTEYVVTRWYRAPEIMCACQDYDRKIDVWSIGCIFGEILGRKPLFPGDNYIHQLNLI PlasB_02211 120 TIEHVQYFLYQILRGLRCIHSAGVLHRDLKPSNLLLNSNCDLKIADFGLARMASAEDDTNA-FMTEYVATRWYRAPEIMLSWKEYSKAIDMWSVGCIFAELLGRRPLFQGRDYMHQLHLI gi\|754344951\|re 108 TDDHCQYFLYQILRGLKYIHSAGVLHRDLKPSNLLLNGNCDLKICDFGLARVAQAGDASAAGFMTEYVATRWYRAPEIMLSWREYTKAIDMWSVGCIFAELLGRRPLFPGKDFLHQLSLI gi\|545707556\|re 119 TEEHFQYFIYQILRALKYVHSADVLHRDLKPSNVLVNGNCDIKICDFGLARSASFN-ELGGEFMTQYVATRWYRAPEIMLSFRHYDKSVDIWSVGCIFAELLGRRPLFPGKDYMHQLRLI gi\|599089093\|gb 119 TDDHCRYFLYQILRGLKYIHSANVLHRDLKPSNLLLNANCDLKVGDFGLARTT----SETD-FMTEYVVTRWYRAPELLLNCSEYTAAIDIWSVGCILGEMMTRQPLFPGRDYVHQLKLI gi\|315258227\|gb 119 TDDHCRYFLYQILRGLKYIHSANVLHRDLKPSNLLLNANCDLKVGDFGLARTT----SETD-FMTEYVVTRWYRAPELLLNCSEYTAAIDIWSVGCILGEMMTRQPLFPGKDYVHQLRLI gi\|719992197\|re 120 SEEHCQYFLYQILRGMKYIHSANVIHRDLKPSNLLLNANCDLKICDFGLARPT----SENE-FMTEYVVTRWYRAPELLLNSSDYTAAIDVWSVGCIFMELMNRKPLFPGRDHVHQMRLL gi\|308808159\|re 114 TDEHCQYFLYQILRGLKYIHSADVLHRDLKPGNLLLNANCDLKICDFGLARTALVDAEASE-FMTEYVVTRWYRAPELLLSCAEYTSAIDVWSVGCIFAELLGRKTLFPGKDYVHQLNLI gi\|159484494\|re 119 SNDHSQYFLYQLLRGLKYIHSANILHRDLKPSNLLVNANCDLKICDFGLARTS--TSNEKE-FMTEYVVTRWYRAPELLLSCSGYTTAIDVWSVGCIFAELLGRKPLFPGKDYVHQLSLI gi\|545374258\|re 116 TDDHFQFFIYQILRGLKYVHTANVLHRDLKPSNLLLNASCDLRICDFGLARTLARQDRYSN-YMTEYVVTRWYRAPELLLSCFQYTAAIDVWSVGCILAELLYRKPLFPGKDYIDQLKLI  gi\|673024676\|re 234 CDKLGTPSEA-ELHFVTSEKARRFMKSQPNKPKIPLDRLFPNVKPTALDLLDKMLVFDPSQRISVEEALQHPYLESLHNCEDEPKATKHFSFEFEK--ENLTKRRLQELIAEEVCHFHP- gi\|813141432\|re 233 CDKLGTPSEE-ELHFVTSEKARRFMKGQPNKPKIPLDKLFPNAKANALDLLDKMLVFDPTKRISVEEALEHPYLESLHNNDDEPKSTKPFSFDFEK--EQLTKRRLQELIFGEVCHFHPD gi\|325188928\|em 231 CDKLGTPTEE-DLHFVTSEKARRFMKSQPKCPKIPFARVFPATKPTAIDLLEKMLIFDPEKRISVEDALAHPYLESLHNEEDEPVAHEAFSFDFEK--EALTKKRLQELILEEICRFHPD gi\|301103436\|re 234 CDKIGTPCEE-DLHFVVSERAKRFMKNQPMRPGVPFAKLYPKASPEAMDLLQRMLVFDPVKRISVEEALEHPYLASLHNLEDEPVADSCFSFDFEK--EDLTESRLKELIFEEILRIHP- PlasB_00238 236 FGTLGTPSDA-DLDWITNAKALQYIKNLKKKPSMPFERVYPKAPPQAIDLLSKMLVFNPANRISVEDALAHPYLKALHNPKDEPICPTTFDFEFEK--QANTKVGIQKLMFEEIEKFRPG PlasB_02211 239 TDVLGTPSFA-DTEYIASPKAKEYIRSLPIKPRIPLQSLYPGAPADALDLLQKMLMFSPEKRISVDEALTHPFLASLHDPTDEPTASGAFDFSFES--VDLDVDTLRDLLWNEAEQYEKS gi\|754344951\|re 228 TDVLGSPSND-DIAGISNDKARRFVRQLPAKPRIPFQTIYPNANPIALDLLQRFLMFNPDNRISVEEALAHPYLAPLHDPADEPTCHSTFNFDFEN--RPLTKEIIKELTFQEMLAFHPP gi\|545707556\|re 238 IDVVGTPSDQ-DIEYIESEKALRFIRSLPRKNPVAWRKLYPDASNLALDLLGRMLQFDPRKRCSVEDALSHPYLSSLHDPTDEPVCPSKFSFEFDS--PQVTKEQLKTMMWEEI------ gi\|599089093\|gb 234 TELIGSPDDA-SLGFLRSDNARRYVRQLPQYPRQQFAAKFPNSSPGAVDLLEKMLVFDPSRRVTVDEALCHPYLAPLHDINEEPVCPMPFSFDFEQ--PSFTEENIKELIWRESVNFNP- gi\|315258227\|gb 234 TELIGSPDDA-SLGFLRSDNARRYVRQLPQYPRQQFAARFPNSSPGAVDLLEKMLVFDPSRRVTVDQALCHPYLAPLHDINEEPICPKPFSFDFEQ--PSFTEENIKELIWRESVKFNP- gi\|719992197\|re 235 TELLGTPAEA-ELGFVKNEDAKRYIRQLPLHPRQSFAHVFPHVHPAAIDLIERMLTFDPTKRITVEEALAHPYLERLHDIADEPVCPEPFSFEFEK--QALVEEQMKEMIYREALAFNP- gi\|308808159\|re 233 MRVIGTPRDDSELDFINNEKARRYIKSLPVTARCNFRKLFPNASPKAVDLVDKMLVLDPARRITVEDALAHPYLESLHDEVDEPCAETPFTFNFEEDGRYLTGTDIRELIYTEL------ gi\|159484494\|re 236 TKVIGSPSEE-ELGFITSEKAKRYIRSLPRSERVDFGQLWPHVTKTALDLIDKMLVFDPTKRITVEQALEHPYLASLHDVSDEPVCPTPFTFDFDS--EHLTPDVVREVILQDMAEL--- gi\|545374258\|re 235 IKMLGSPCDS-DLVFISSSKARAYIKALPYAQRCPFRVMFPDASHLAIDLMEKMLQFNPERRITVEQALAHPYLAQMHDPASELSAPDTFDFDFEE--QELVEAAVRSKVLEEIQ----- |
| PlasB_05287 (PbSGK) | XP_002768442.1: RAC-beta serine/threonine-protein kinase-A, putative [*Perkinsus marinus* ATCC 50983]  XP_008890014.1 AGC protein kinase [*Phytophthora parasitica* INRA-310]  XP_009843660.1 AGC protein kinase [*Aphanomyces astaci*]  XP_008872442.1 AGC protein kinase [*Aphanomyces invadans*]  XP_012209992.1 AGC protein kinase [*Saprolegnia parasitica* CBS 223.65]  XP_008616359.1 AGC protein kinase [*Saprolegnia diclina* VS20]  XP_012204850.1 AGC protein kinase [*Saprolegnia parasitica* CBS 223.65] | XP_002768442.1 1 ------VPKDKVSLEDFVLIKVIGKGSYGKVMLVRYK-KDNNVYAMKMLRKENVMKRNQVEHTRTERNVLETVSHPFIVNLVYAFQTPKKLYFILEYCPGGELFFHLSRAQRFSENRCRF XP_012209992.1 1 -----------VALDDFHLIKVIGKGSFGKVLLVRKK-DNGVVYAMKVLRKENIIKRNQVEHTRTERHVLGYVRHPFIVGLNYAFQTSEKLYFVLDYCAGGELFFHLGKVQRFKEPRAQF XP_008616359.1 1 -------------LDDFHLIKVIGKGSFGKVLLVRKK-DNGVVYAMKVLRKENIIKRNQVEHTRTERHVLGYVRHPYIVGLNYAFQTSEKLYFVLDYCAGGELFFHLGKVQRFKEPRAQF XP_009843660.1 1 -----------VTLEDFHLVKVIGKGSFGKVILVRKR-DSGIVYAMKVLRKENIIKRNQVEHTRTERHVLGYVRHPFIVGLNYAFQTVDKLYFVLDYCAGGELFFHLGKVQRFKEPRARF XP_008872442.1 1 -----------VTLDDFHLVRVIGKGSFGKVILVRKK-DSGIVYAMKVLRKENIIKRNQVEHTRTERHVLGYVRHPFIVGLNYAFQTVDKLYFVLDYCAGGELFFHLGKVQRFKEPRARF XP_008890014.1 1 -----------VTLEDFVMIKVIGKGSFGKVLLVRKR-DTGLIYAMKVLRKENIIKRNQVEHTRTERHVLGYVRHPFIVGLNYAFQTSEKLYFVLDYCAGGELFFHLGKVQRFPEHRARF XP_012204850.1 21 -----LYNTPKTRLDDFELLKVIGKGSYGKVTLVRKK-DSKKLYAMKTLSKPNVKRRNQVEHTRTERRVLGLTKHPYIVHLHYAFQTTQKLYFVIDYCPGGELFFHLSRMERFSESMACF PlasB_05287 121 IKQSLSVPTVPASLDDFQLLKVIGKGAFGKVMLVRSKSDPKQLFALKQLDKSKVRNKGQSEHAMTERNILEYIRHPFIVTMRGAFQTESKLFIVLDYCAGGELFFHLQRMGTFSEELARF  XP_002768442.1 114 YASEILLAIEYLHKYDIVYRDLKPENVLLDADGHVKLTDFGLSKEGIMDNSS-AKSMCGT XP_012209992.1 109 YAAEITLAIEYVHNLDIIYRDLKPENVLLDENGHIRLTDFGLSKEGIQDDFSGANSFCGT XP_008616359.1 107 YAAEITLAIEYVHNLDIIYRDLKPENVLLDENGHIRLTDFGLSKEGIQDDFSGANSFCGT XP_009843660.1 109 YAAEITLAIEYVHNLDIIYRDLKPENVLLDENGHIRLTDFGLSKEGIQDDFSGANSFCGT XP_008872442.1 109 YAAEITLAIEYVHNLDIIYRDLKPENVLLDENGHIRLTDFGLSKEGIQDDFSGANSFCGT XP_008890014.1 109 YAAEITLAIEYVHNLDVIYRDLKPENVLLDENGHIRLTDFGLSKEGIQDDFSGANSFCGT XP_012204850.1 135 YAAEIVLALDHLHQLGVVYRDLKPENILFDATGHVLLADFGLAKEGITDGAEGTNSMCGT PlasB_05287 241 YTAQLVLAIEYLHELGIVFRDLKLENVLLDAKGNIQLTDFGLSKEGVADNVS-ARSFCGT |
| PlasB_03214 (PbCDK) | gi\|698550246: cell division control protein 2 homolog A [*Nicotiana sylvestris*]  gi\|908212408: cyclin-dependent protein kinase p34cdc2 [*Solanum lycopersicum*]  gi\|1321672: cyclin-dependent kinase [*Antirrhinum majus*]  gi\|17224978: cyclin dependent kinase [*Helianthus annuus*]  gi\|359489125: cell division control protein 2 homolog [*Vitis vinifera*]  gi\|672166647: cell division control protein 2 homolog A [*Phoenix dactylifera*]  gi\|743828567: cell division control protein 2 homolog A isoform X1 [*Elaeis guineensis*]  gi\|695049759: cell division control protein 2 homolog A isoform X1 [*Musa acuminata* subsp. *malaccensis*]  gi\|357114348: cyclin-dependent kinase A-1 [*Brachypodium distachyon*]  gi\|923132459: cell division control protein 2 [*Thecamonas trahens* ATCC 50062] | gi\|698550246\|re 1 ---------------MDQYEKVEKIGEGTYGVVYKARDRVTNETIALKKIRLEQEDEGVPSTAIREISLLKEMQHANIVRLQDVVHSE-KRLYLVFEYLDLDLKKHMDSSPEFSKDPRLV gi\|908212408\|re 1 ---------------MDQYEKVEKIGEGTYGVVYKARDRVTNETIALKKIRLEQEDEGVPSTAIREISLLKEMQHANIVRLQDVVHSE-KRLYLVFEYLDLDLKKHMDSCPEFSKDPRLV gi\|1321672\|emb\| 1 ---------SSHRSLMEQYEKVEKIGEGTYGVVYKARDRVTNETIALKKIRLEQEDEGVPSTAIREISLLKEMQHGNIVRLQDVVHSE-KRLYLVFEYLDLDLKKHMDSCPEFSQDPRLV gi\|17224978\|gb\| 1 ---------------MEQYEKVEKIGEGTYGVVYKARDKVTNETIALKKIRLEQEDEGVPSTAIREISLLKEMQHGNIVRLQDVVHSD-KRLYLVFEYLDLDLKKHMDSCPEFSKDPRLV gi\|359489125\|re 1 ---------------MDQYEKVEKIGEGTYGVVYKARDRVTNETIALKKIRLEQEDEGVPSTAIREISLLKEMQHGNIVRLQDVVHSE-KRLYLVFEYLDLDLKKHMDSSPDFAKDLRLI gi\|672166647\|re 1 ---------------MDQYEKVEKIGEGTYGVVYKARDRLTNEMIALKKIRLEQEDEGVPSTAIREISLLKEMQHNNIVRLQDVVHSE-KRIYLVFEYLDLDLKKHMDSCPEFARDSRLI gi\|743828567\|re 1 ---------------MDQYEKVEKIGEGTYGVVYKARDRLTNETIALKKIRLEQEDEGVPSTAIREISLLKEMQHNNVVRLQDVVHSE-KRIYLVFEYLDLDLKKHMDSCPEFAKDPRLV gi\|695049759\|re 1 ---------------MDQYEKVEKIGEGTYGVVYKARDRLTNETIALKKIRLENEDEGVPSTAIREISLLKEMQHNNIVRLQDVVHSE-KRIYLVFEYLDLDLKKHMDSCPELVKDPRLV gi\|357114348\|re 1 ---------------MEQYEKVEKIGEGTYGVVYKARDKATNETIALKKIRLEQEDEGVPSTAIREISLLKEMQHGNIVKLHDVVHSE-KRIYLVFEYLDLDLKKFMDSCPEFAKNPTLI gi\|923132459\|re 1 -----------------QYMKLEKIGEGTYGVVYKARDLLTNDFIALKKIRLDAEDEGVPSTAIREISLLKELEHPNIVRLHDVVHSD-KKLYLVFEYLDQDLKKYMDSVSGLLK-PALV PlasB_03214 1 MAGDGERPESQQYAGLGRYQKIEKIGEGTYGVVYKARDRVTGESIALKKIRLDSEDEGIPSTAIREISLLKELQHPNVVSLRDVVHHDFKKLYLVFEYLDQDLKKYMDS-QTAPLDLMLV  gi\|698550246\|re 105 KMFLYQILRGIAYCHSHRVLHRDLKPQNLLIDRRTNALKLADFGLARAFGIPVRTFTHEVVTLWYRAPEILLGSRHYSTPVDVWSVGCIFAEMVTQRPLFPGDSEIDELFKIFRVMGTPN gi\|908212408\|re 105 KMFLYQILRGIAYCHSHRVLHRDLKPQNLLIDRRTNALKLADFGLARAFGIPVRTFTHEVVTLWYRAPEILLGSRHYSTPVDVWSVGCIFAEMVNQRPLFPGDSEIDELFKIFRVMGTPN gi\|1321672\|emb\| 111 KMFLYQILRGIAYCHSHRVLHRDLKPQNLLIDRRTNALKLADFGLARAFGIPVRTFTHEVVTLWYRAPEILLGSRHYSTPVDVWSVGCIFAEMVNQRPLFPGDSEIDELFKIFRVMGTPN gi\|17224978\|gb\| 105 KTFLYQILRGIAYCHSHRVLHRDLKPQNLLIDRRTNALKLADFGLARAFGIPVRTFTHEVVTLWYRAPEILLGSRHYSTPVDVWSVGCIFAEMVNQRPLFPGDSEIDELFKIFRIMGTPN gi\|359489125\|re 105 KMFLHQILRGIAYCHSHRVLHRDLKPQNLLIDRRTNALKLADFGLARAFGIPVRTFTHEVVTLWYRAPEILLGSRHYSTPVDVWSVGCIFAEMVNQRPLFPGDSEIDELFKIFRVLGTPN gi\|672166647\|re 105 KTYLYQILRGIAYCHSHRVLHRDLKPQNLLIDRRTNALKLADFGLARAFGIPVRTFTHEVVTLWYRAPEILLGSRHYSTPVDVWSVGCIFAEMVNQKPLFPGDSEIDELFKIFRVLGTPN gi\|743828567\|re 105 KKYLHQILCGIAYCHSHRVLHRDLKPQNLLIDRRTNALKLADFGLARAFGIPVRTFTHEVVTLWYRAPEILLGSRHYSTPVDVWSVGCIFAEMVNQKPLFPGDSEIDELFKIFRVLGTPN gi\|695049759\|re 105 KTYLYQILRGIAYCHSHRVLHRDLKPQNLLIDRQTNVLKLADFGLARAFGIPVRTFTHEVVTLWYRAPEILLGSRHYSTPVDVWSIGCIFAEMVNQRPLFPGDSEIDELFKIFRVLGTPN gi\|357114348\|re 105 KSYLYQILRGVAYCHSHRVLHRDLKPQNLLIDRRTNALKLADFGLARAFGIPVRTFTHEVVTLWYRAPEILLGARQYSTPVDVWSVGCIFAEMVNQKPLFPGDSEIDELFKIFRVLGTPN gi\|923132459\|re 102 KSYLQQLLEGIAFCHSHRVLHRDLKPQNLLIDR-NGVLKLADFGLARAFGIPVRTYTHEVVTLWYRAPEILLGSRHYSCPVDVWSIGCIFAEMASKIPLFPGDSEIDNLFRIFKILGTPN PlasB_03214 120 KSYMQQLLKGIAFCHSHRTLHRDLKPQNLLIDR-QGALKLADFGLARAFGVPVRPYTHEVVTLWYRAPEILLGTKEYSTPVDIWAAGCIFAELVTKQPLFPGDSEIDELFRIFRALGTPN  gi\|698550246\|re 225 EDTWPGVTTLPDFKSAFPKWPSKDLATIVPNLDGAGLDLLDKMLRLDPSKRITARNALEHEYFKDI----- gi\|908212408\|re 225 EDTWPGVTSLPDYKSAFPKWPPKDLAIIVPNVDGAGLDLLGKMLSLDPSKRITARNALEHEYFKDI----- gi\|1321672\|emb\| 231 EETWPGVTSLPDFKSAFPKWPAKELAAVVPNLDASGLDLLDKMLRLDPSKRITARNALQHEYFKDI----- gi\|17224978\|gb\| 225 EETWPGVTSLPDFKSAFPKWSSKDLATVVPNLEKTGLDLLRKMLCLDPSKRITARTALEHEYFKDI----- gi\|359489125\|re 225 EDTWPGVTSLPDFKSAFPKWPPKDLATVVPNLESAGIDLLSKMLCLDPSRRITARSALEHEYFKDI----- gi\|672166647\|re 225 EETWPGVTSLPDFKSAFPKWPPKDLAAMVPNLEPAGIDLLSKMLRLEPSKRITARQALEHEYFKDL----- gi\|743828567\|re 225 EETWPGVTSLPDFKSAFPKWPPKDLATVAPNLEPAGIDLLSKMLRLEPSKRITARKALEHEYFKDL----- gi\|695049759\|re 225 EETWPGVTSLPDFKSAFPKWLPKDLATAVPNLEATGIDLLSKMLRLDPSKRVTARQALEHEYFKDF----- gi\|357114348\|re 225 EQTWPGVSSLPDYKSAFPRWQAEDLATIVPNLEPVGLDLLSKMLRFEPNKRITARQALEHDYFKDM----- gi\|923132459\|re 221 ETIWPGVSALPDFKSSFPKWQPKNLASVATNLGPDGIDLLSQMLEYEPSRRISAKAALSHPYF-------- PlasB_03214 239 ETTWPRVTSMPDYKSTFPKWDPRPLSTCVPGLDRVGLDLLGKMLRYEPGKRISAKQALEHAYFDDLYDLQR |
| PlasB_00247 (PbPKA1) PlasB_07097 (PbPKA2) | gi\|672823213: AGC/PKA protein kinase [*Mortierella verticillata* NRRL 6337]  gi\|511008541: AGC/PKA protein kinase [*Mucor circinelloides* f. *circinelloides* 1006PhL]  gi\|574114641: AGC/PKA protein kinase [*Aphanomyces astaci*]  gi\|906559600: AGC/PKA protein kinase [*Thecamonas trahens* ATCC 50062]  gi\|340503630: protein kinase domain protein [*Ichthyophthirius multifiliis*]  gi\|126117347: protein kinase, cAMP-dependent, catalytic chain [*Cryptosporidium parvum* Iowa II]  gi\|570969359: AGC/PKA protein kinase [*Phytophthora parasitica* P10297]  gi\|262105868: cAMP-dependent protein kinase catalytic subunit, putative [*Phytophthora infestans* T30-4]  gi\|325186835: cAMPdependent protein kinase catalytic subunit putat [*Albugo laibachii* Nc14]  gi\|530743445: AGC/PKA protein kinase [*Saprolegnia* *diclina* VS20] | gi\|672823213\|gb 1 -----LIYPVASPCYDARPRYGLSDFELLETLGTGTFGRVYLTKFRHDHS--FYAMKVLKKTEVVRLKQVEHINSEKQILSQVHFPFIVNLFTTFQDERNLYMLLEYVIGGELFSHLRKA gi\|511008541\|gb 1 -----HFYHTRPPAYVEKPSFGLKDFELQDTLGTGTFGRVYLSKFVPSSK--YYAMKVLKKSEVVRLKQVEHLLSEKQILSSVRFPFIVDLFCTFQDESNLYMLLEYVVGGELFTHLRRA gi\|574114641\|gb 1 ---------------MMKVEY--DSFEILATLGTGTFGRVRLVKHKENNT--YYALKILKKCEIIRLQQLHHIKCEVEILSKIDHPFIVNFLGSFQDEKRLYLVLEYVPGGELFSYLRRQ gi\|906559600\|gb 1 -----MPNIAGLSLEDNMFELNIAEFDLISTIGTGTFGRVYLARHMTTSK--YYAMKVLKKVEVVRLKQVEHITSEKNILSVIHHPFIVNLFGATQDAKNLYMLLEYVIGGEVFTHLRRA PlasB_00247 1 ------------EYLASRENLSLSTFTIGATLGTGTFGRVRLATIAIDGKPKYMALKMLKKTEIIRLKQVEHIKDEKRILSAICHPFIVNFFGSFQDEKRLYMIMEYVIGGELFSQLRRA gi\|340503630\|gb 1 -----------------LNQIKLSDFEIMQTLGTGSFGRVRLAKQKSTGE--YVALKMLKKAEILRLKQVDHIISENTILSNINHPFLIKMLGFCQDDRFLYFVLEYIQGGELFTYLRNK gi\|126117347\|gb 1 -----------------QKKYSIDDFQLIRTLGTGSFGRVFLSKHKEDNS--IYAIKRLKKSVVIRQKQVDHITNEKAILSRIKHPFLVRMFGTFKDDRYLYIMMEFVIGGEFFTYLRRC PlasB_07097 1 LKESLGKAGSVEAMQALKNLWKLSDFELRETLGTGSFGRVRFAKHIPSGK--FYAIKILKKAEILRLKQVDHILSEKTILQAINHPFIVNMFASFQDKKYLYLAMEYVVGGEFFTHLRRA gi\|570969359\|gb 1 ----------------FAAPLDGSQFDMGVTLGTGSFGRVRFATHKATNT--YWAIKILKKAEIIRLQQVEHMLSEKSILLCLDHPFIVNLAGTFQDTKYLYMVLEYVIGGEFFTHLRKA gi\|262105868\|gb 1 ----------------FAAPLDGSQFDMGVTLGTGSFGRVRFATHKATNT--YWAIKILKKAEIIRLQQVEHMLSEKSILLCLDHPFIVNLAGTFQDTKYLYMVLEYVIGGEFFTHLRKA gi\|325186835\|em 1 ----------------LAAPLDASQFEMGVTLGTGSFGRVRFATHKSTST--YWAIKILKKAEIIRLQQVEHMLSEKSILLCLDHPFVVNLAGTYQDPKYLYMVLEYIVGGEFFTHLRKA gi\|530743445\|gb 1 ----------------LPGPLDIAHFEFGVTLGTGSFGRVRFATHKATGS--FWAIKILKKAEIIRLQQVEHMISEKTILMCLDHPFIVNLAGTFQDAKCLYMVLEYVIGGEFFTHLRKA  gi\|672823213\|gb 114 GRFTNDMTRFYAAEIVLAIEYLHSRDIIYRDLKPENLLLDSTGHVKITDFGFAKRVEDRTWTLCGTPEYLAPEIIQSKGHGKAVDWWALGILIFEMLAGYPPFFDDNPFGIYEKILAGRI gi\|511008541\|gb 114 GRFTNDMTRFYASEIVLAIEYLHSKDIIYRDLKPENLLIDHQGHIKITDFGFAKKVVDRTWTLCGTPEYLAPEIIQSKGHGKAVDWWALGILIFEMLAGYPPFFDDNSFGIYEKILMGKV gi\|574114641\|gb 102 GKLTDDASRYYASQLVLAIAYLHGLHIIYRDLKPENLLITHDGSIKITDFGFAKRVEDKTWTLCGTPEYLAPEIIQSKGHGKSVDWWAIGVLIYEMLAGYPPFYDENPFGIYQKILTGKV gi\|906559600\|gb 114 GKFPPDVTKFYTAEIVLALEYMHSLDIIYRDLKPENLLLDSNGHIKITDFGFAKKVEDRTWTLCGTPEYLAPEIIQSKGHGKAVDWWALGILVYEMLCGYPPFFSDNPFSIYEAILAGKI PlasB_00247 109 RRFTNDASRFYAVEIVLALEFCHERNIVYRDLKPENILIDKEGHVKITDFGFAKFVEDRTWTLCGTPEYLAPEIIQSKGHGKAVDWWALGVLIFEMCAGYPPFYDENPFGIYQKILDGTI gi\|340503630\|gb 102 GKLENNESLFYGAQVVSMFEYLHGINIVYRDLKPENILIGANGYLKLTDFGFAKYCDSRTYTLCGTPEYLAPEILLNKGHGKPVDWWCLGILIYEMLAGIDPFNDEDPMAIYQKILKGKV gi\|126117347\|gb 102 RHFDNETSRFYAAQVVLMFEYLHGKNIIYRDLKPENILIDKDGYLKLTDFGFAKAIEYRTFTLCGTPEYIAPEVLLNKGHGKPVDWWTLGILIYEMVVGFPPFYDDEPMGIYQKILAGKI PlasB_07097 119 GRFNNDSSCFYAAQIVLIFQYLHSKDIVYRDLKPENLLLDAKGNIKMTDFGFAKKVEFRTWTLCGTPEYIAPEILLNKGHGKPVDWWALGILIYEMLAGAPPFVDDDPMGIYQKILAGRI gi\|570969359\|gb 103 GRFDNNTAKFYAAQVVSIFEYMHSQDFIYRDLKPENLLLDNEGYIKITDFGFAKRVAFKTYTLCGTPEYIAPEVLLNKGHGKGVDWWTLGILLYEMLAGQPPFCDDDPMGIYQQILSGKL gi\|262105868\|gb 103 GRFDNNTAKFYAAQVVSIFEYMHSQDFIYRDLKPENLLLDNEGYIKITDFGFAKRVAFKTYTLCGTPEYIAPEVLLNKGHGKGVDWWTLGILLYEMLAGQPPFCDDDPMGIYQQILSGKL gi\|325186835\|em 103 GRFDNNTTKFYASQVVSIFEYLHAQDFVYRDLKPENLLLDAEGYLKLTDFGFAKRVAFKTYTLCGTPEYIAPEVLLNKGHGKGVDWWTLGILMYEMLAGQPPFCDEDPMGIYQQILSGRV gi\|530743445\|gb 103 GRFDFNTTKFYATQVVSIFEYLHSQDFIYRDLKPENLLLDQEGFIKITDFGFAKRVAFKTYTLCGTPEYIAPEVLLNKGHGKGVDWWTMGILIYEMLAGQPPFCDDDPMGIYQQILSGKI  gi\|672823213\|gb 234 YFPPHIDPVAKDLIKKLLTADRTKRLGNLKDGSDDIKNHKWFRGVDWKGLLDKTVTAPIVPPYVHPGDTGNFEKYPEV-VDDGAGQLG--DPYRSLFMNF- gi\|511008541\|gb 234 QFTAHFDPLAKDLLKRLLVSDRTKRLGNLKGGSEDVKRHKWFRGVDWVGLLDKNVRAPIVPHYPHPGDTSNFEKYPEQ-FDTDHHNSNGEDPFKDLFVDFS gi\|574114641\|gb 222 EYPRYIDSKAKDLIKKLLTHDRTKRLGCLRNGAEDVKKHKYFANVDWNSVISKGDTPPYLPPIHGPGDHGNFDQYPES-TEDTAVVLTGDDKAAFDAFNLF gi\|906559600\|gb 234 AFPSHLDSAAKDLIRNLLNADLTKRIGCLRNGPADIKRHKWYKSIDWDALLAKQIPAPIIPVTTSPGDTSNFDQYEEE-PDD-PSDPNGEDPFAELFAGF- PlasB_00247 229 DFPKHFDPNAKDLVRRLLTADRTKRYGCLKNGVDDIKEHRWFKGIDWDAALQRKLTPPFIPTAMSENDTTNFDKYPDS-VER-GHPPEISAADAALFEAF- gi\|340503630\|gb 222 KFPKNFDKNAKSLVKHLLVADLTKRYGNLKGGVSDIKTHRWFGDIDWDALNQQKIQAIYKPIIKGKNDTQNYGSYPDS-IELPKAIQSQDDPFINW----- gi\|126117347\|gb 222 FFPKYFDKNCKSLVKRLLTPDLTKRYGNLKGGVSDIKLHKWFYNYDFNSLISRKVDPPYIPKVNSYDDSSNFEEYPDS-HEQPTTVTGNADPFVDW----- PlasB_07097 239 EYPAHFNRHAKDLISRLLTPDITNRLGNLKNGVEDIKRHKWFANINWTKLYNRKMLTPFVPKVRGDDDTHNFDHFPDS-AEPPADVVLENDPFENF----- gi\|570969359\|gb 223 NFPRFFDRNAKGLIKRMLTADLTKRYGCLKNGVEDIKKHKFFTGINWEDLLARKGAAPIIPRVGTANDTSNFDPYPDSMEDAIVPVYNGKDPFAEF----- gi\|262105868\|gb 223 NFPRFFDRNAKGLIKRMLTADLTKRYGCLKNGVEDIKKHKFFTGINWEDLLARKGAAPIIPRVGTANDTSNFDPYPDSMEDAIVPVYNGKDPFAEF----- gi\|325186835\|em 223 SFPRFFDRNAKAIIKRLLTADLTKRYGSLKNGVDDIKKHKYFSGVNWTDLLARKSKAPIIPRFDTPNDTSNFDPYPDSADDVIVPVYNGKDPFVDF----- gi\|530743445\|gb 223 NFPRYFDRNAKALIKRLLTADLTKRYGCLKNGVEDIKKHKFFSGVDWDAMLARKGTAPIIPKVVTPNDTSNFDPYPDSNEEAPVPVYNGKDPFAEF----- |
| PlasB_01916 (PbGβγ) | gi\|119174510: guanine nucleotide-binding protein subunit beta [*Coccidioides immitis* RS]  gi\|48237421: small G-beta protein GPB [*Paracoccidioides brasiliensis*]  gi\|145254573: guanine nucleotide-binding protein subunit beta [*Aspergillus niger* CBS 513.88]  gi\|849275022: guanine nucleotide-binding protein subunit beta [*Neosartorya udagawae*]  gi\|584411925: guanine nucleotide-binding protein subunit beta [*Penicillium roqueforti* FM164]  gi\|262348234: heterotrimeric G-protein beta subunit [*Monascus ruber*]  gi\|671147388: guanine nucleotide-binding protein subunit beta [*Cyphellophora europaea* CBS 101466]  gi\|751835513: guanine nucleotide binding protein (G protein), beta 1 [*Rhizoctonia solani* AG-1 IB]  gi\|913842989: guanine nucleotide-binding protein subunit beta [*Puccinia sorghi*] | gi\|119174510\|re 1 ----MDSE-QLQAKITAARREAEGLKDRIKRRK--DELADTTLRQVAQNNTDPLPRIGMRPRRNLKGHLAKIYAMHWSTDRRHLVSASQDGKLIIWDAYTTNKVHAIPLRSSWVMTCAYA gi\|48237421\|gb\| 1 MAADLSGE-QMQAKITAARREAEGLKDRIKRRK--DELADTSLRQVAQNNTDPLPRIGMRPRRNLKGHLAKIYAMHWSTDRRHLVSASQDGKLIIWDAYTTNKVHAIPLRSSWVMTCAYA gi\|145254573\|re 1 -MADMSGE-QMQAKITAARREAEGLKDKIKRRK--DELADTTLRQVAQNQTETLPRIGMKPRRTLKGHLAKIYAMHWSTDRRHLVSASQDGKLIIWDAYTTNKVHAIPLRSSWVMTCAYA gi\|849275022\|db 1 MAADMSGE-QMQAKITAARREAEGLKDKIKRRK--DELADTTLRQVAQNQTDTLPRIGMKPRRTLKGHLAKIYAMHWSTDRRHLVSASQDGKLIIWDAYTTNKVHAIPLRSSWVMTCAYA gi\|584411925\|em 1 -MADMSGE-QMQAKITAARREAEGLKDKIKRRK--DELADTSLRQVAQNQTDALPRIGMKPRRTLKGHLAKIYAMHWSTDRRHLVSASQDGKLIIWDAYTTNKVHAIPLRSSWVMTCAYA gi\|262348234\|gb 1 MAADMTGE-QLQAKITAARREAEGLKDKIRRRK--EELADTTLRQVAQSQTDPLPRIMMKPRRTLKGHLAKIYAMHWSTDRRHLVSASQDGKLIIWDAYTTNKVHAIPLRSSWVMTCAYA gi\|671147388\|re 1 MSADMTSE-QMQAKITAARREAEGLKDRIKRKK--DDLADTSLRQVAQNNTEPLPRIGMKPRRNLKGHLAKIYAMHWSTDRRHLVSASQDGKLIIWDAYTTNKVHAIPLRSSWVMTCAYA gi\|751835513\|em 1 ----MSNQGDIQERMAAARREAEQLKEKIRARR--DASADTTLRAMTE-DLESLPRVVMRPRRTLKGHLAKIYAMHWAADKRHLVSASQDGKLIVWDAYTTNKVHAIPLRSSWVMTCAYS gi\|913842989\|gb 1 ----MST--DIQEKIAAARREAETLKEKIRAKR--DQLADTTLRAMAA-EIDPLPRIPIKPRRTLKGHLAKIYAMHWAQDQRHLVSASQDGKLIVWDAYTTNKVHAIPLRSSWVMTCAYA PlasB_01916 1 ----MTTK----EKIEKCKKEIEDLKATIRDLRGDSDSADQPLQRVAREQGVSPGTCSIKQRRILKGHFGKIYAMHWSDDSRHLVSASQDGKLIVWNAFSTNKVHAIPLRSSWVMTCAYS  gi\|119174510\|re 114 PSGNYVACGGLDNICSIYNLSSREG-PTRVARELSGHSGYLSCCRFINDRRIITSSGDMTCMLWDIESGTKVTEFADHLGDVMSISINPTNNNIFVSGACDAFAKLWDTRV-DK-AVQTF gi\|48237421\|gb\| 118 PSGNYVACGGLDNICSIYNLSSREG-PTRVARELSGHTGYLSCCRFVNDRRIITSSGDMTCMLWDIESGSKVTEFADHLGDVMSISINPTNNNVFVSGACDAFAKLWDIRA-GK-SVQTF gi\|145254573\|re 117 PSGNYVACGGLDNICSIYNLSSREG-PTRVARELSGHSGYLSCCRFINDRRIITSSGDMTCMLWDIESGSKVTEFADHLGDVMSISINPTNQNVFVSGACDAFAKLWDIRT-GK-AVQTF gi\|849275022\|db 118 PSGNYVACGGLDNICSIYNLSSREG-PTRVARELSGHSGYLSCCRFINDRRIITSSGDMTCMLWDIESGSKVTEFADHLGDVMSISINPTNQNIFVSGACDAFAKLWDIRT-GK-AVQTF gi\|584411925\|em 117 PSGNYVACGGLDNICSIYNLSSREG-PTRVARELSGHSGYLSCCRFINDRRIITSSGDMTCMLWDIESGTKVTEFADHLGDVMSISINPTNQNIFVSGACDAFAKLWDIRT-GK-SVQTF gi\|262348234\|gb 118 PSGNYVACGGLDNICSIYNLSSREG-PTRVARELSGHSGYLSCCRFINDRRIITSSGDMTCMLWDIESGSKVTEFADHLGDVMSISINPTNQNVFVSGACDTFAKLWDIRT-GK-AVQTF gi\|671147388\|re 118 PSGNFVACGGLDNICSIYNLSTREG-PTRVARELSGHSGYLSCCRFVNDRKIITSSGDMTCMMWDIETGSKVTEFADHLGDVMSISINPTNANVFVSGACDAFAKLWDVRT-GK-AVQTF gi\|751835513\|em 114 PSGNFVACGGLDNICSIYNLRNKEGGGNKSARELSAHSGYLSCCRFINDRQIVTSSGDMTCMLWDIEAGARVMEFNDHTGDVMSLSLGP-NQNVFVSGACDATAKLWDIRS-GK-ATQTF gi\|913842989\|gb 112 PSGNFVACGGLDNICSIYNLRSKEG-TVRVARELSAHTGYLSCCRFLNDRQILTSSGDMSCMLWDIEAGVRVVEFNDHSGDVMSLSLGP-NLNTFVSGACDATAKVWDVRT-GK-VVQSF PlasB_01916 113 PQGTFVACGGLDNLCSVYKLSNKAEGQQRTHCELAQHEGYLSCCRFINEGEIITSSGDSTCILWDIEQKQPKNTFSDHAGDVMSVSIFGPEGKTFVSGSCDATAKLWDTRADGKSAVKTF  gi\|119174510\|re 231 SGHESDINAIQFFPDGNAFGTGSDDTSCRLFDIRADRELNIYQSDQVLCGITSVAFSVSGRLLFAGYDDFECKVWDVLRGDKVGSLSGHENRVSCLGVSNDGISLCTGSWDSLLKIWAW gi\|48237421\|gb\| 235 SGHESDINAIQFFPDGNAFGTGSDDTTCRLFDIRADRELNTYQSDQVLCGITSVAFSVSGRLLFAGYDDYECKVWDVLRGEKVGSLSGHENRVSCLGVSNDGISLCTGSWDSLLKVWAW gi\|145254573\|re 234 AGHESDINAIQFFPDGNAFGTGSDDTSCRLFDIRADRELNTYQSDQILCGITSVAFSVSGRLLFAGYDDFECKVWDVLRGDKVGSLSGHENRVSCLGVSNDGISLCTGSWDSLLKVWAW gi\|849275022\|db 235 AGHESDINAIQFFPDGNAFGTGSDDTSCRLFDIRADRELNIYQSDQILCGITSVAFSVSGRLLFAGYDDFECKVWDVLRGDKVGSLSGHENRVSCLGVSNDGISLCTGSWDSLLKVWAW gi\|584411925\|em 234 AGHESDINAVQFFPDGNAFGTGSDDTSCRLFDIRADRELNTYQSDQVLCGITSVAFSVSGRLLFAGYDDFECKVWDVLRGDKVGSLSGHENRVSCLGVSNDGISLCTGSWDSLLKVWAW gi\|262348234\|gb 235 NGHESDINAVQFFPDGNAFGTGSDDATCRLFDIRADRELNIYQSDQVVCGITSVAFSVSGRLLFAGYDDFECKVWDVLRGDKVGSLSGHENRVSCLGVSNDGISLCTGSWDSLLKVWAW gi\|671147388\|re 235 AGHESDINAIQFFPDGNAFGTGSDDASCRLFDIRADRELMSYQNEQVLCGITSVAFSVSGRLLFAGYDDFECKVWDTLRGDKVGSLSGHENRVSCLGVSNDGISLCTGSWDSLLKIWAW gi\|751835513\|em 231 TGHESDINAVQFFPNGDAFATGSDDASCRLFDIRADRELNSFTHDNILCGITSVAFSISGRVLFGGYDDWTCNVWDTLKGERVGVLTGHENRVSCLGVSADGMALCTGSWDSTLKVWA- gi\|913842989\|gb 228 VGHESDINTVCFFPNGDAFATGSDDASCRLFDLRADRELNQYTHDNVLCGITSVAFSASGRILFAGYDDFNCNVWDTLKGERVGVLAGHENRVSCLGVSSDGMALCTGSWDSMLKVWA- PlasB_01916 233 PGHESDINSVMFFPDGNAFGTGSDDSSCRLFDIRAYRQLNKYSSDKILCGITSVAFSKTGKMLFAGYDDYNCYVWDTLLGSNPSQLSGHENRVSCLGVAADGKALCTGSWDTFLKIWA- |
| PlasB_02182 (PbCaM) | gi\|575479244\|ref\|XP_006678916.1\|: Calmodulin [*Batrachochytrium dendrobatidis* JAM81]  gi\|351715366\|gb\|EHB18285.1\|: Calmodulin [*Heterocephalus glaber*]  gi\|521028958\|gb\|EPQ10746.1\|: Calmodulin [*Myotis brandtii]*  gi\|46517823\|gb\|AAQ20043.1\|: Calmodulin [*Pinctada fucata*]  gi\|907097539\|gb\|KND03011.1\|: Calmodulin [*Spizellomyces punctatus* DAOM BR117]  gi\|779176942\|ref\|NP_001292553.1\|: Calmodulin isoform 1 [*Homo sapiens*]  gi\|602652254\|ref\|XP_007432500.1\|: PREDICTED: calmodulin isoform X1 [*Python bivittatus*]  gi\|946658228\|ref\|XP_014430289.1\|: PREDICTED: Calmodulin-A isoform X2 [*Pelodiscus sinensis*]  gi\|528761907\|gb\|EPY81566.1\|: Calmodulin-like protein [*Camelus ferus*]  gi\|545366875\|ref\|XP_005648308.1\|: CaM [*Coccomyxa subellipsoidea* C-169] | gi\|575479244\|re 15 ------AAVCDIDADQLTEEQIAEFKEAFSLFDKDGDGTITTKELGTVMRSLGQNPTEAELQDMINEVDADGNGTIDFPEFLTMMARKMKDTDSEEEIKEAFKVFDKDGNGFISAAELRH gi\|907097539\|gb 1 ------------MADQLTEEQIAEFKEAFSLFDKDGDGTITTKELGTVMRSLGQNPTEAELQDMINEVDADGNGTIDFPEFLTMMARKMKDTDSEEEIKEAFKVFDKDGNGFISAAELRH PlasB_02182 1 ---------MATPDKQLTDEQIAEFKEAFSLFDKDGDGTITTKELGTVMRSLGQNPTEAELQDMINEVDADGNGTIDFPEFLTMMARKMKDTDSEDEIREAFKVFDKDGNGFISAAELRH gi\|351715366\|gb 38 SVTHYAEDLTSPPADQLTEEQIAEFKEAFSLFDKDGDGTITTKELGTVMRSLGQNPTEAELQDMINEVDADGNGTIDFPEFLTMMARKMKDTDSEEEIREAFRVFDKDGNGYISAAELRH gi\|521028958\|gb 36 -----QDGVPSAAADQLTEEQIAEFKEAFSLFDKDGDGTITTKELGTVMRSLGQNPTEAELQDMINEVDADGNGTIDFPEFLTMMARKMKDTDSEEEIREAFRVFDKDGNGYISAAELRH gi\|545366875\|re 1 ----------MTEAAQLTDEQVAEFKEAFALFDKDGDGTITTKELGTVMRSLGQNPTEAELQDMINEVDADGNGTIDFPEFLNLMARKMKDSDSEEELREAFKVFDKDGNGFISAAELRH gi\|46517823\|gb\| 1 ------------MADQLTEEQIAEFKEAFSLFDKDGDGTITTKELGTVMRSLGQNPTEAELQDMINEVDADGNGTIDFPEFLTMMARKMKDTDSEEEIREAFRVFDKDGNGFISAAELRH gi\|528761907\|gb 61 --RVLRRGETVSPADQLTEEQIAEFKEAFSLFDKDGDGTITTKELGTVMRSLGQNPTEAELQDMINEVDADGNGTIDFPEFLTMMARKMKDTDSEEEIREAFRVFDKDGNGYISAAELRH gi\|779176942\|re 37 LYIASHFAQQPCKADQLTEEQIAEFKEAFSLFDKDGDGTITTKELGTVMRSLGQNPTEAELQDMINEVDADGNGTIDFPEFLTMMARKMKDTDSEEEIREAFRVFDKDGNGYISAAELRH gi\|946658228\|re 6 ------------KADQLTEEQIAEFKEAFSLFDKDGDGTITTKELGTVMRSLGQNPTEAELQDMINEVDADGNGTIDFPEFLTMMARKMKDTDSEEEIREAFRVFDKDGNGYISAAELRH gi\|602652254\|re 28 ------------QADQLTEEQIAEFKEAFSLFDKDGDGTITTKELGTVMRSLGQNPTEAELQDMINEVDADGNGTIDFPEFLTMMARKMKDTDSEEEIREAFRVFDKDGNGYISAAELRH  gi\|575479244\|re 129 VMTNLGEKLTDEEVDEMIREADVDGDGQINYEEFVKMMMSK gi\|907097539\|gb 109 VMTNLGEKLTDEEVDEMIREADVDGDGQINYEEFVKMMMSK PlasB_02182 112 VMTNLGEKLTDEEVDEMIREADVDGDGQINYEEFVKMMMAS gi\|351715366\|gb 158 VMTNLGEKLTDEEVDEMIREADIDGDGQVNYEEFVQMMTAK gi\|521028958\|gb 151 VMTNLGEKLTDEEVDEMIREADIDGDGQVNYEEFVQMMTAK gi\|545366875\|re 111 VMTNLGEKLTDEEVDEMIREADVDGDGQVNYEEFVKMMMAK gi\|46517823\|gb\| 109 VMTNLGEKLTDEEVDEMIREADIDGDGQVNYEEFVKMMMSK gi\|528761907\|gb 179 VMTNLGEKLTDEEVDEMIREADIDGDGQVNYEEFVQMMTAK gi\|779176942\|re 157 VMTNLGEKLTDEEVDEMIREADIDGDGQVNYEEFVQMMTAK gi\|946658228\|re 114 VMTNLGEKLTDEEVDEMIREADIDGDGQVNYEEFVQMMTAK gi\|602652254\|re 136 VMTNLGEKLTDEEVDEMIREADIDGDGQVNYEEFVQMMTAK |
| PlasB_10022 (PbCyclin) | gi\|3608420: cyclin A [*Dreissena polymorpha*]  gi\|922511727: cyclin-A1 [*Brassica oleracea* var. *oleracea*]  gi\|545710129: cyclin A [*Galdieria sulphuraria*]  gi\|449444835: G2/mitotic-specific cyclin S13-7 [*Cucumis sativus*]  gi\|514821156: cyclin-A3-1-like [*Setaria italica*]  gi\|802010: cyclin A [*Helobdella robusta*]  gi\|571448501: mitotic cyclin a2-type isoform X1 [*Glycine max*]  gi\|646723041: G2/mitotic-specific cyclin-A [*Zootermopsis nevadensis*]  gi\|908429288: G2/mitotic-specific cyclin-A-like [*Biomphalaria glabrata*]  gi\|502132267: cyclin-A2-1-like [*Cicer arietinum*] | gi\|3608420\|gb\|A 1 GYMK--------------------RQQDITSSMRSILVDWLVEVAEEYKLHRETLFLAVNYIDRFLSKISVLRGKLQLVGAASMFLAAKYEEIYPP--DVTEFAYITDDTYDKKQILRME PlasB_10022 1 NLIDEFSNMLVNEQKFHAEMSYVDDYRQFSYDMRSRLLGWIVEVLSYVDASEQTLFLAAAIFDRFFRLGGCHKALWALLAGTSIFIASKYYQISAVR--VRLITEALHNTYTRQDVYDME gi\|922511727\|re 1 --------------------------------MRTILIDWLVEVAEEYRLLPETLYLAVNCLDRYLSGNVITKQNLQLLGVSCMMIASKYEEVCVP--QVESFCYITDNTYSRNELLEME gi\|545710129\|re 1 SYVDDMK----------------ATQSEISPNMRAILMDWLVEVAEEYKLSNETLHLACNYIDRFLSRCSVSKKNLQLLGVVCLLVASKYEEKYPP--HVDEFVYITDNTYTKEEVLSME gi\|449444835\|re 1 EYIDDMYKFYKLAEGESIVSDYMGTQPDLNAKMRSILIDWLIEVHRKFELMPETLYLAVNIVDRFLSLKTVPRKELQLVGISSMLIACKYEEIWAP--EVNDFVSISANTYQREQILVME gi\|514821156\|re 1 --------------------------KAISPKMRAVLVDWLVEVADEFKLQAETLYLAVSYVDRFLTMNVVTRDKLQLLGVTALLLAAKYEEIESSKMKVNRYTDITDNTYTKQQVVKME gi\|802010\|gb\|AA 1 NYME--------------------RQSDINHSMRSILVDWLVEVADEYKLKRETFFLAVNYIDRFLSMMSVIRCRLQLLGAAAMFIAAKYEEIYPP--DVAEFVYITDDTYTMKQVLQME gi\|571448501\|re 1 NYMDK-------------------LQKDINPSMRGILVDWLVEVSEEYKLVPDTLYLTVNLIDRYLSTRLIQKQKLQLLGVTCMLIASKYEEMCAP--RVEEFCFITDNTYTKEEVLKME gi\|646723041\|gb 1 GYMR--------------------KQPDITYAMRSILVDWLVEVAEEYRLQTETLYLAVSYIDRFLSFMSVVRAKLQLVGTAAMFIAAKYEEIYPP--DVREFVYITDDTYTKKQVLRME gi\|908429288\|re 1 TYMR--------------------KQTDVSTSMRNILVDWLVEVGEEYKLQRETLFLAISYIDRFLSLVGVHRPKLQLVGAASMFIAAKYEEIYPP--DVAEFVYITDDTYTRSQVLKME gi\|502132267\|re 1 NYMDK-------------------LQQDISPNMRGILVDWLVEVSEEYKLVPDTLYLTVNLIDRFLSTKLIQKHRLQLLGVTCMFIASKYEEICAP--RVEEFCFITDNTYTKEEVVRME  gi\|3608420\|gb\|A 99 HLILKVLAFDVAIPTTNWFCESFLKSID----AEEKL-----------KSLTMFLSELTLIEMDSYLKYVPSITASACIC PlasB_10022 119 MAVLGRLDFNISLVTAWDFAVFFLSRLP---SLPQDGE-------TVIRSMTSYLLELALVDRM-HFDMRPSSLAASCIA gi\|922511727\|re 87 SSVLNYLKFELTTPTAKCFLRRFVRAAK----GKKEVT------SLLFESLASYLSELSLLDYA-MLRYAPSLVAASAVF gi\|545710129\|re 103 MLVMKVLKFSFTAASSYQFASIFGSWGN----LNEVV-----------KSISFFLCDLSLVDFS-LSKYLPSDIATAAVC gi\|449444835\|re 119 KVILGRLEWLLTVPTPYVFLVRYVKASE----PSDDE----------MENMVFFLAELGLMNYQISISYSPSTIASAAVY gi\|514821156\|re 95 ADLLKSLNFEIGGPTVTTFLRRFIASCRGGNCTSSEK----------LESMCSYLAELSLLDYDC-ISYLPSVLAAACLF gi\|802010\|gb\|AA 99 QAILKTLNFLVAAPTPNYFCCDLLDRLG----AYKKGPNGVCDDGGRWNSLAMYMLETTTVFGDCFLKFSPSMISTSCVA gi\|571448501\|re 100 REVLNLVHFQLSVPTIKTFLRRFIQAAQ----SSYKAP------YVELEFLANYLAELALVECS-FFQFLPSLIAASAVF gi\|646723041\|gb 99 HLILKVLAFDLSVPTTLSFITSFSVSND----LSEMT-----------MFLAMYLGELSLLEADPYLQYVPSELAAACIA gi\|908429288\|re 99 SVILKLLNFKVAVPTINWFCERFLDLLE----MTEKA-----------KSLTHFLTELSLIEVEEFLDFRPSVIAASAIV gi\|502132267\|re 100 SEVLNLLRFQLCVPTTKTFLRRFIQAAQ----SSYKVP------RIELEFLANYLAELTLVEYN-YLQFLPSLVAASAVF |
| PlasB_03329 (PbACTB_G1) | XP_004350653.1/ actin [*Cavenderia fasciculata*]  AAQ55798.1/ actin [*Vannella ebro*]  XP_004337269.1/ actin-1, putative [*Acanthamoeba castellanii* str. Neff]  XP_004336648.1/Actin1 [*Acanthamoeba castellanii* str. Neff]  XP_004335674.1/ Actin1 [*Acanthamoeba castellanii* str. Neff]  JAU98306.1/ Actin, plasmodial isoform, partial [*Noccaea caerulescens*]  AFL56351.1/ beta-actin [*Tachypleus tridentatus*]  CCG28027.1/ actin II [*Sepia officinalis*]  XP_016607082.1/ actin-A3b, cytoplasmic [*Spizellomyces punctatus* DAOM BR117] | XP_004350653.1 1 MSD-DVQALVIDNGSGMCKAGFAGDDAPRAVFPSIVGRPRHTGVMVGMGQKDSYVGDEAQSKRGILTLKYPIEHGIVTNWDDMEKIWHHTFYNELRVAPEEHPVLLTEAPLNPKANREKM AAQ55798.1 1 -D-EVQALVVDNGSGMCKAGFAGDDAPRAVFPSIVGRPRHTGVMVGMGQKDSYVGDEAQSKRGILTLKYPIEHGIVTNWDDMEKIWHHTFYNELRVAPEEHPVLLTEAPLNPKANREKM XP_004337269.1 1 MGD-EVQALVIDNGSGMCKAGFAGDDAPRAVFPSIVGRPRHTGVMVGMGQKDSYVGDEAQSKRGILTLKYPIEHGIVTNWDDMEKIWHHTFYNELRVAPEEHPVLLTEAPLNPKANREKM XP_004336648.1 1 MGD-EVQALVIDNGSGMCKAGFAGDDAPRAVFPSIVGRPRHTGVMVGMGQKDSYVGDEAQSKRGILTLKYPIEHGIVTNWDDMEKIWHHTFYNELRVAPEEHPVLLTEAPLNPKANREKM XP_004335674.1 1 MGD-EVQALVIDNGSGMCKAGFAGDDAPRAVFPSIVGRPRHTGVMVGMGQKDSYVGDEAQSKRGILTLKYPIEHGIVTNWDDMEKIWHHTFYNELRVAPEEHPVLLTEAPLNPKANREKM JAU98306.1 1 MSDGDVQALVVDNGSGMCKAGFAGDDAPRAVFPSIVGRPRHKGVMVGMGQKDAYVGDEAQSKRGILTLKYPIEHGIVTNWDDMEKIWHHTFYNELRVAPEEHPVLLTEAPLNPKANREKM AFL56351.1 1 --D-EVAALVVDNGSGMCKAGFAGDDAPRAVFPSIVGRPRHQGVMVGMGQKDSYVGDEAQSKRGTLTLKYPIEHGIVTNWDDMEKIWHHTFYNELRVAPEEHPVLLTEAPLNPKANREKM CCG28027.1 1 MDD-EVAALVIDNGSGMCKAGFAGDDAPRAVFPSIVGRPRHQGVMVGMGQKDSYVGDEAQSKRGILTLKYPIEHGIVTNWDDMEKIWHHTFYNELRVAPEEHPVLLTEAPLNPKANREKM XP_016607082.1 1 MSD-DVAALVIDNGSGMCKAGFAGDDAPRAVFPSIVGRPRHQGVMVGMGQKDSYVGDEAQSKRGILTLKYPIEHGIVTNWDDMEKIWHHTFYNELRVAPEEHPVLLTEAPLNPKANREKM PlasB_03329 1 MSD-EVQALVVDNGSGMVKAGFAGDDAPRAVFPSIVGRPRHVGIMVGATQKDAYVGDEAQAKRGILTLKYPIEHGIVTNWDDMEKIWHHTFYNELRVAPEEHPVMLTEAPLNPKANREKM   XP_004350653.1 120 TQIMFETFNTPAMYVAIQAVLSLYASGRTTGIVMDSGDGVSHTVPIYEGYALPHAILRLDLAGRDLTDYLMKILTERGYSFTTTAEREIVRDIKEKLAYVALDFESEMQTAATSSQLEKS AAQ55798.1 118 TQIMFETFNTPAMYVAIQAVLSLYASGRTTGIVLDSGDGVSHTVPIYEGYALPHAILRLDLAGRDLTDYMMKILTERGYSFTTTAEREIVRDIKEKLAYVALDFEQEMQTAASSSALEKS XP_004337269.1 120 TQIMFETFNTPAMYVAIQAVLSLYASGRTTGIVMDSGDGVTHTVPIYEGYALPHAILRLDLAGRDLTDYLMKILTERGYSFTTTAEREIVRDIKEKLCYVALDFEQEMATAASSSSLEKS XP_004336648.1 120 TQIMFETFNTPAMYVAIQAVLSLYASGRTTGIVMDSGDGVTHTVPIYEGYALPHAILRLDLAGRDLTDYLMKILTERGYSFTTTAEREIVRDIKEKLCYVALDFEQEMATAAASSSLEKS XP_004335674.1 120 TQIMFETFNTPAMYVAIQAVLSLYASGRTTGIVLDSGDGVTHTVPIYEGYALPHAILRLDLAGRDLTDYLMKILTERGYSFTTTAEREIVRDIKEKLCYVALDFEQEMHTAASSSALEKS JAU98306.1 121 TQIMFETFNTPAMYVAIQAVLSLYASGRTTGIVLDSGDGVSHTVPIYEGYALPHAIMRLDLAGRDLTDYLMKILTERGYSFTTTAEREIVRDIKEKLCYVALDFEQEMSTAASSSALEKS AFL56351.1 118 TQIMFETFNTPAMYVAIQAVLSLYASGRTTGIVLDSGDGVSHTVPIYEGYALPHAILRLDLAGRDLTDYLMKILTERGYSFTTTAEREIVRDIKEKLCYVALDFDQEMATAASSSSLEKS CCG28027.1 120 TQIMFETFNTPAMYVAIQAVLSLYASGRTTGIVMDSGDGVTHTVPIYEGYALPHAILRLDLAGRDLTDYLMKILTERGYSFTTTAEREIVRDIKEKLCYVALDFDQEMQTAASSSSLEKS XP_016607082.1 120 TQIMFETFNTPAFYVAIQAVLSLYASGRTTGIVLDSGDGVSHTVPIYEGYALPHAILRLDLAGRDLTDYLMKILTERGYSFTTTAEREIVRDIKEKLCYVALDFEQEMQTAAQSSALEKS PlasB_03329 120 TQIMFETFNTPAMYVAIQAVLSLYASGRTTGIVLDAGDGVSHTVPIYEGYALPHAIQRLDIAGRDITDYLMKILTERGYSFTTTAEREIVRDIKEKLAYVAQDFDTEVSNSEQSSEVERA  XP_004350653.1 240 YELPDGQVITIGNERFRCPEALFQPSFLGMESAGIHETTYNSIMKCDVDIRKDLYGNVVLSGGTTMFPGIADRMNKELTALAPSTMKIKIIAPPERKYSVWIGGSILASLSTFQQMWISKEEYDESGPSIVHRKCF AAQ55798.1 238 YELPDGQVITIGNERFRCPEALFQPSFLGMESAGVHETTYNSIMKCDVDIRKDLYGNVVLSGGSTMFPGIAERMNKELVALAPSTMKIKVIAPPERKYSVWIGGSILASLSTFQQMWISKEEYDESGPSIVHRKCF XP_004337269.1 240 YELPDGQVITIGNERFRCPETLFQPSFLGMEAAGIHETTYNSIMKCDVDIRKDLYGNVVLSGGTTMFPGIADRMQKELTALAPSTMKIKIIAPPERKYSVWIGGSILASLSTFQQMWISKEEYDESGPSIVHRKCF XP_004336648.1 240 YELPDGQVITIGNERFRCPETLFQPSFLGMEAAGIHETTYNSIMKCDVDIRKDLYGNVVLSGGTTMFPGIADRMQKELTALAPSTMKIKIIAPPERKYSVWIGGSILASLSTFQQMWISKEEYDESGPSIVHRKCF XP_004335674.1 240 YELPDGQVITIGNERFRAPEALFQPSFLGMESAGIHETTYNSIMKCDVDIRKDLYGNVVLSGGTTMFPGIADRMQKELTALAPSTMKIKIIAPPERKYSVWIGGSILASLSTFQQMWISKEEYDESGPSIVHRKCF JAU98306.1 241 YELPDGQVITIGNERFRCPEALFQPSFLGMESAGIHETTFNSIMKCDVDIRKDLYGNVVLSGGTTMFPGIGDRMQKELTALAPSTMRIKIIAPPERKYSVWIGGSILASLSTFQQMWISKEEYDESGPAIVHRKCF AFL56351.1 238 YELPDGQVITIGNERFRCPEALFQPSFLGMESSGIHETTYNSIMKCDIDIRKDLYANIVLSGGTTMYPGIADRMQKEITALAPSTMKIKIIAPPERKYSVWIGGSILASLSTFQQMWISKQEYDESGPSIVHRKCF CCG28027.1 240 YELPDGQVITIGNERFRAPEAMFQPSFLGMESAGIHETTYNSIMKCDVDIRKDLYANTVLSGGSTMFPGIADRMQKEISALAPATMKIKIIAPPERKYSVWIGGSILASLSTFQQMWISKQEYDESGPSIVHRKCF XP_016607082.1 240 YELPDGQVITIGNERFRAPEALFQPSFLGLEAAGIHETTYNSIMKCDVDIRKDLYGNIVLSGGTTMYPGIADRMQREITALAPSSMKIKIVAPPERKYSVWIGGSILASLSTFQQMWISKQEYDESGPSIVHRKCF PlasB_03329 240 YELPDGQVITVGNERFRAPEVLFQPSMIGNESEGIHQLAYQSIMKCDVDIRKDLYENIVMSGGSTMYDGLATRLQKEVAALAPSSMRVKVVAPPERKYSVWIGGSILSSLSTFQQMWVSKEEYDEAGPSIVHRKCF |
| PlasB_01918 (PbMOB1) | KYR02650.1: Mps1 binder-like protein [*Dictyostelium lacteum*]  XP_004356628.1: Mob4B protein isoform 3, putative [*Acanthamoeba castellanii* str. Neff]  XP_647813.1: Mps1 binder-like protein [*Dictyostelium discoideum* AX4]  NP_001072572.1: MOB kinase activator 1B [*Xenopus tropicalis*]  NP_001085265.1: MOB kinase activator 1B L homeolog [*Xenopus laevis*]  XP_020435327.1: Mps1 binder-like protein [*Polysphondylium pallidum* PN500]  XP_004364593.2: mps one binder kinase activator-like 1 [*Capsaspora owczarzaki* ATCC 30864]  XP_004361170.1: Mps1 binder-like protein [*Dictyostelium fasciculatum*]  XP_011092604.1: MOB kinase activator-like 1A [*Sesamum indicum*] | XP_004356628.1 1 SIFGLG-KKSQTFKPHKNIPEGTKQYQLKKYAEATLGSGNLKLAVTLPEGEDINEWLAVNTVDFFNQINMLYGTITEFCTPQECPVMSAGPKYEYMWADGVNVKKAIKCSAPEYVDFLMT XP_004364593.2 1 MSWIFT-KGSKTFKPKRNIPEGTKQYQLKKYAEATLGSGNLRLAVTLPEGEDLNEWVAVNTVDFFNQINMLYGTITEFCTAEECPVMSAGPKYEYHWADGQNVKKPIKCSAPEYIDFLMT XP_020435327.1 1 --NIFN-KKSQTFKPKKNIQEGSKQYQLKQYAEATLGSGNLRLAVSLPAGEDLNEWLAVNTVDFFNQINMLYGTITEFCTPSECPVMSAGNKYEYHWADGTTVKKAIKVSAPEYVDYLMT XP_004361170.1 1 ---MFS-KKSQTFKPKKNIQEGSKQYQLKQYAEATLGSGNLRLAVSLPAGEDLNEWLAVNTVDFFNQINMLYGTITEFCTTSECPVMSAGPKYEYHWADGTTVKKAIKVSAPEYVDYLMT XP_647813.1 1 --NIFG-KKSQTFKPKKNIQEGSKQYHLKQYAEATLGSGNLKSAVSLPTGEDINEWLAVNTTDFFNQINMLYGTITEFCTGTDCPVMSAGPKYEYHWADGTTVKKAIKVSAPEYVDFLMT NP_001072572.1 1 MSFLFGSRSSKTFKPKKSLPEGSHQYELLKHAEATLGSGNLRMAVMLPEGEDLNEWVAVNTVDFFNQINMLYGTITDFCTEESCPVMSAGPKYEYHWADGTNIKKPIKCSAPKYIDYLMT NP_001085265.1 1 MSFLFGNRSSKTFKPKKSLPEGSHQYELLKHAEATLGSGNLRMAVMLPEGEDLNEWVAVNTVDFFNQINMLYGTITDFCTEESCPVMSAGPKYEYHWADGTNIKKPIKCSAPKYIDYLMT KYR02650.1 1 MFKIFS-DKNKTFKPKKGFNKGTKRHDLHKHAKATLGSGNLRLAVSLPEREDLNEWLAVNTVDFFNQINLLYGSITEFCTAKSCEVMSAGPKYEYLWADGESVKKPIKVSAPEYVDYLMT XP_011092604.1 1 SLFGLG-RNQRTFRPKKSAPSGSKGAQLRKHIDATLGSGNLREAVRLPPGEDINEWLAVNTVDFFNQVNLLYGTLTEFCTPENCPTMSAGPKYEYRWADGVQIKKPIEVSAPKYVEYLMD PlasB_01918 1 MKGIFG-MGQKTFKPTKSHEKGSKRDEMHLKAQATLGSGDMAMAVKLPKGEDMNEWLAVNTVDFYKYISLLYGTIAEFCTAESCPAMSAGDAFQYLWADGVKIKKPIKCSAPEYVDHLMS  XP_004356628.1 120 WVQEQLDDEEIFPSKVGVPFPKSFVQ-IVGNIFKRLFRVYAHIYHSHFPKIVSLGEEAHLNTSFKHFIFFVQEFSLINKKELQPLAELISSLTNK XP_004364593.2 120 WVQGQLDDETIFPSKIGVPFPKSFQA-TAKNILKRLFRVYAHIYHSHFNKIVSLGEEAHLNTSFKHFIFFVQEFNLIEKKELAPLAELISQLTNK XP_020435327.1 118 WVQSQLDDETIFPSKIGVMFPKNFQS-IVKTIFKRLFRVYAHIYHSHFQKIVNLGEEAHLNTSLKHFIFFIQEFNLVDKKELGPLAELIDTLI-- XP_004361170.1 117 WVQSQLDDESIFPSKIGVLFPKNFQS-IVKTIFKRLFRVYAHIYHSHFQKIVNLGEEAHLNTSLKHFIFFIQEFNLVDKKELGPLSELIETLI-- XP_647813.1 118 WVQSQLDDENIFPSKIGVPFPKNFQS-IVKTIFKRLFRVYAHIYHSHFQKIVSLGEEAHLNTSLKHFIYFIQEFNLVDKKELGPLNELIESLM-- NP_001072572.1 121 WVQDQLDDETLFPSKIGVPFPKNFMS-VAKTILKRLFRVYAHIYHQHFDSVIQLQEEAHLNTSFKHFIFFVQEFNLIDRREQAPLQELIEKLTSK NP_001085265.1 121 WVQDQLDDETLFPSKIGVPFPKNFMS-VAKTILKRLFRVYAHIYHQHFDSVIQLQEEAHLNTSFKHFIFFVQEFNLIDRREQAPLQELIEKLTSK KYR02650.1 120 WVQNILDDENIFPSRVDVQFPKNFQS-IVKNIFKRLFRVYGHIYYSHFLKIVSLGEEAHLNTCFKHFYFFIVEFNLVDKKEMIPLQDLIDNLT-- XP_011092604.1 120 WIETQLDDESIFPQRLGAPFPSNFRE-VVKTIFKRLFRVYAHIYHSHFQKIVSLKEEAHLNTCFKHFILFTCEFSLIDKKELAPLQELIESIV-- PlasB_01918 120 WVESQLNDEHIFPLQIGAPFPKNFQTQIIPTLFKRLFRVYAHLYHEHFTKMQALGAEAHLNTCFKHFMFFVREFNLIDKKEQEPLKDLIENLVSQ |
| Note: Datasets of selected proteins were assembled by using BLASTP with query sequences of key components associated with cancer-related signaling pathways of *P. brasicae*. Protein sequence alignments were performed by using the ClustalW algorithm with additional manual adjustments. Numbers on the left indicate the positions of the amino acid residues. Identical residues are shaded in black, and similar residues are shaded in gray. The information of NCBI database accession number of homologs was list at below. | | |
